# Supplementary material for: State of the Art and New Trends from the Second International StemNet Meeting
Source: Int J Mol Sci. 2024 Feb 13;25(4):2221. doi: 10.3390/ijms25042221 (PMC10889812; doi:10.3390/ijms25042221)
Supplement: Supplementary file 1 [file ijms-25-02221-s001.zip › ijms-2867762-supplementary.pdf]

Conference Report

State of the Art and New Trends from the Second International StemNet Meeting

Ivana Ferrero [1], Filippo Piccinini [2,3], Pasquale Marrazzo [3], Manuela Monti [2], Caterina Pipino [4], Alessia Santa Giovanna Banche Niclot [5], Camilla Francesca Proto [1], Enrico Ragni [6], Ralf Hass [7], Giulia Maria Stella [8,9], Priscilla Berni [10], Ana Ivanovska [11] and Katia Mareschi [1,5,*]

1 Stem Cell Transplantation and Cellular Therapy Laboratory, Paediatric Onco-Haematology Division, Regina Margherita Childrens' Hospital, City of Health and Science of Turin, 10126 Turin, Italy; iferrero@cittadellasalute.to.it (I.F.); cproto@cittadellasalute.to.it (C.F.P.)
2 IRCCS Istituto Romagnolo per lo Studio dei Tumori (IRST) ''Dino Amadori", 47014 Meldola, Italy; filippo.piccinini@irst.emr.it (F.P.); manuela.monti@irst.emr.it (M.M.)
3 Department of Medical and Surgical Sciences (DIMEC), University of Bologna, 40126 Bologna, Italy; pasquale.marrazzo2@unibo.it
4 StemTeCh Group, Center for Advanced Studies and Technology-CAST, Department of Medical, Oral and Biotechnological Sciences, University G. D'Annunzio Chieti-Pescara, 66100 Chieti, Italy; caterina.pipino@unich.it
5 Department of Public Health and Paediatrics, University of Turin, 10126 Turin, Italy; alessiagiovannasanta.bancheniclot@unito.it
6 Laboratorio di Biotecnologie Applicate all'Ortopedia, IRCCS Istituto Ortopedico Galeazzi, Via Cristina Belgioioso 173, 20157 Milano, Italy; enrico.ragni@grupposandonato.it
7 Biochemistry and Tumour Biology Laboratory, Department of Obstetrics and Gynecology, Hannover Medical School, D-30625 Hannover, Germany; hass.ralf@mh-hannover.de
8 Department of Internal Medicine and Medical Therapeutics, University of Pavia Medical School, 27000 Pavia, Italy; giuliamaria.stella@unipv.it
9 Unit of Respiratory Diseases, Cardiothoracic and Vascular Department, IRCCS San Matteo Hospital Foundation, 27100 Pavia, Italy
10 Department of Veterinary Sciences, University of Parma, 43121 Parma, Italy; priscilla.berni@unipr.it
11 Regenerative Medicine Institute (REMEDI), University of Galway, H91 TK33 Galway, Ireland; ana.ivanovska@universityofgalway.ie
* Correspondence: katia.mareschi@unito.it

SUPPLEMENTARY MATERIALS

ORAL PRESENTATIONS

O-01 BIOMEDICAL COMMUNICATION IN ITALY: CRITICAL ASPECTS

Daniele Banfi[1]
[1] Scientific journalist, Umberto Veronesi Foundation, newspapers within the Gedi Group (Repubblica, La Stampa)

The Covid-19 pandemic has laid bare all the problems related to health journalism. The infodemic, namely the circulation of an excessive amount of decontextualized information that makes it difficult to navigate a topic, is nothing more than the consequence of lacking investments in quality information. The seminar will analyse, with concrete examples, the history of health journalism in our country, providing points for reflection on how to improve scientific communication - also from the perspective of researchers and university institutions.

O-02 BIOMEDICAL COMMUNICATION IN ITALY: CRITICAL ASPECTS

Agnese Collino[1]
[1] Umberto Veronesi Foundation and BergamoScienza (Milan)

Under the umbrella term "communicator," various figures come together, united by a common denominator: the dedication to conveying science to the public. The goals, methods, and languages of those who do so through a school educational workshop, an Instagram or TikTok channel, or a scientific festival are very different from each other, just as their challenges and limits vary. We will discuss what it means to communicate science—especially health—today, to better understand the different players in this diverse world and how even those involved in research can be part of it.

O-03 Biomedical Communication in Italy: Good and Bad Practice

Alessandro Iapino[1]
[1] Press Office Manager and Editorial Coordination Head, Bambino Gesù Hospital

The dramatic experience of the pandemic has shown us how the theme of science and health communication can be crucial not only for people's lives but also for civil life and the resilience of democratic institutions. Like all crises, it has glaringly revealed the strengths and weaknesses of the "system" in which we live, even in terms of communication. From this perspective, it can be a great opportunity to look at biomedical communication with new, more attentive, and conscious eyes, starting from our concrete experience. We should strive to recognize and correct our mistakes, our bad practices, and also acknowledge what works or has worked.

The pandemic has taught us (though we knew it before) that we cannot avoid communicating science. It is not a choice we have. We cannot presume that communicating science is not important or crucial, pandemic or no pandemic. We cannot be naive in thinking that we can withdraw from communication in today's society – there is no outside. Every subject communicates, even unintentionally. Silence is also a choice of communication, sometimes the best one. The difference lies in how we communicate: effectively or poorly.

The pandemic has taught us (though we knew it before) that communicating science is never "just" about communicating science. Communication, even scientific communication, is never just about the content but always about the context. And the context is never neutral or necessarily "peaceful." Scientific communication is always "political."

The pandemic has taught us (though we knew it before) that even when we communicate science, we are not only conveying something – what we know – but always someone. We communicate who we are within a context and a relationship. The credibility of who we are conditions, for those who listen, the credibility of what we say. Communication, even scientific communication, is the sharing of common sense and belonging.

The pandemic has taught us (though we knew it before) that communication is always a relationship, and the relationship is always between different parties. We do not communicate only among doctors, not only among scientists. Communication always requires a significant effort of translation, upon which the understanding of the message depends. There is no communication without translation. There is no scientific communication without simplification.

The pandemic has taught us (though we knew it before) that communicating science is not advertising who we are or what we do, but primarily serving and listening to the concrete needs of our interlocutors, where they express themselves. The main space for expressing these needs is the digital sphere, the environment of the network in which we are all immersed. However, the pandemic has shown the gap in the digital presence and effectiveness of our scientific, biomedical, and institutional communication. Think about the case of the IMMUNI APP.

The pandemic has taught us (though we knew it before) that even when we communicate science, the medium, the channel, the platform conditions the message (as McLuhan said, the medium is the message). Web, social media, and television are not neutral environments. The medium conditions who we are, what we say, and how we say it. Communication, even scientific communication, requires specific skills, languages, awareness, organisation, and strategy. Being good scientists or proficient doctors is not enough to be good communicators.

STEMNET NEXT GENERATION SESSION

O-04 FMR1 silencing and stability during human FXS brain development: from an early stage of pluripotency to 3D cortical organoids

Cecilia Laterza[1], Elisa Cesare[1], Hannah Stuart[2], Martina D'Ercole[1], Onelia Gagliano[1], Silvia Angiolillo[1], Roberta Polli[3], Alessandra Murgia[3], Nicola Elvassore[1].
[1] Department of Industrial Engineering, University of Padova, 6/a Via Gradenigo, Padova 35131, Italy; Veneto Institute of Molecular Medicine, 2 Via Orus, Padova 35131, Italy.
[2] The Francis Crick Institute, 1 Midland Road, London NW1 1AT, UK; Research Institute of Molecular Pathology (IMP), Vienna BioCenter (VBC), Campus-Vienna-BioCenter 1, 1030 Vienna, Austria.
[3] Laboratory of Molecular Genetics of Neurodevelopment, Department of Woman and Child Health, University of Padova, 35128 Padova, Italy; Fondazione Istituto di Ricerca Pediatrica, Città della Speranza, 35128 Padova, Italy.

Fragile X Syndrome (FXS) is an X-linked neurodevelopmental disorder and the main form of inherited intellectual disability. It is caused by the expansion of a CGG sequence to more than 200 repeats in the promoter of the fragile X mental retardation 1 gene (FMR1) that determines its silencing through methylation during embryonic development. However, the exact timing and cellular compartment at which this instability occurs during early human embryonic development are still unknown, due to the inaccessibility of the human tissues during this time frame.
A human model able to mimic the early stage of neural development is instrumental to answering these open questions associated with the complex alterations of the FMR1 gene.
We generated a unique in vitro model of human cortical development based on naïve induced pluripotent stem cells (iPSCs). A human model based on naïve iPSCs has the potential to reproduce the neural development from an epigenetic ground state of early pluripotency, which is characterised by a broad unmethylated genome, to a late neural differentiation in brain organoids, which recapitulates the lineage-specific genetic and epigenetic regulation and pathogenetic modifications of FMR1 locus.
We followed the neural differentiation of FXS cortical organoids for up to 4 months and we observed either that: i) hypermethylation of FMR1 locus occurred very rapidly during neural differentiation and both FMR1 mRNA and protein were downregulated; ii) FMR1 locus methylated only partially (in longer alleles) and the unmethylated alleles underwent shortening falling in the premutation category; iii) FMR1 locus did not methylate leading to an increased expression of FMR1 mRNA more than 5 folds compared to the healthy control associated to the presence of intranuclear inclusions, typical of permutations.
The results of our work could enlighten key targetable mechanisms of FXS pathogenesis, clarifying which cell type is more sensitive to FMR1 silencing and helping the development of suitable treatments for FXS patients.

O-05 Contraceptive drug, Nestorone, enhances stem cell-mediated remodelling of the stroke brain by dampening inflammation and rescuing mitochondria

Jea-Young Lee[1*], **Vanessa Castelli** [1,2*], Narender Kumar [2], Regine Sitruk-Ware[3], Cesario Borlongana
*Co-first Authors
[1] Center of Excellence for Aging and Brain Repair, Department of Neurosurgery and Brain Repair, University of South Florida College of Medicine, 12901 Bruce B. Downs Blvd, Tampa, FL, 33612, USA
[2] Department of Life, Health and Environmental Sciences, University of L'Aquila, 67100, Italy
[3] Population Council, Center for Biomedical Research, 1230 York Avenue, New York, NY, 10065, USA

Ischemic stroke remains a significant unmet need causing massive mortality and morbidity due to few treatment options with limited therapeutic window. The progestin Nestorone® (progesterone acetate) displays high affinity for the progesterone receptor in exerting its potent birth control and hormone replacement therapy. Accumulating evidence implicates a new utility of Nestorone in affording neuroprotection in a variety of central nervous system diseases, including stroke. However, the mechanism of action mediating Nestorone's neuroprotection in stroke remains unknown. Here, we showed that stand-alone treatments of Nestorone or human amniotic fluid-derived stem cells (hAFSc), but more pronounced with their

combined treatment, led to significant improvements in behavioural function and reductions in infarction and peri-infarct cell loss in adult rats with ischemic stroke. We detected significantly lower levels of pro-inflammatory signals (OX6 and IBA1) coupled with enhanced levels of stem cell proliferation (Ki67) and differentiation (DCX and MAP2) in both brain and spleen of stroke rats that received stand-alone or combined treatments of Nestorone and hAFSc. In concert, the in vitro oxygen-glucose deprivation stroke model revealed that neural stem cells treated with Nestorone exhibited increased stem cell proliferation and differentiation that was accompanied by rescue of the mitochondrial respiratory activity characterized by reduced mitochondrial reactive oxygen species, increased ATP, elevated mitochondrial deacetylase Sirtuin 3 (SIRT3), and a normalized ratio of acetyl-superoxide dismutase 2 (Ac-SOD2)/SOD2, suggesting the key role of mitochondrial metabolism and oxidative protection in Nestorone's therapeutic effects in stroke.

O-06 Effect of the Human Amniotic Membrane on the Umbilical Vein Endothelial Cells of Gestational Diabetic Mothers: New Insight on Inflammation and Angiogenesis

Caterina Pipino[1*†], Ángel Bernabé-García[2†], Ilaria Cappellacci[1], Javier Stelling-Férez[2,3], Pamela Di Tomo[1], Manuela Santalucia[1], Carlos Navalón[2], Assunta Pandolfi1[‡] and Francisco José Nicolás[2*‡]
[1] Center for Advanced Studies and Technology-CAST (ex CeSI-MeT), Department of Medical, Oral and Biotechnological Sciences, University G. D'Annunzio Chieti-Pescara, StemTeCh Group, Chieti, Italy
[2] Regeneration, Molecular Oncology and TGFß, IMIB-Arrixaca, Hospital Clínico Universitario Virgen de La Arrixaca, Murcia, Spain
[3] Department of Nutrition and Food Technology, UCAM, Murcia, Spain

*Correspondence: Caterina Pipino caterina.pipino@unich.it; Francisco José Nicolás franciscoj.nicolas2@carm.es
[†] These authors have contributed equally to this work and share first authorship
[‡] These authors have contributed equally to this work and share last authorship

One of the most relevant diabetes complications is impaired wound healing, mainly characterized by reduced peripheral blood flow and diminished neovascularization together with increased inflammation and oxidative stress. Unfortunately, effective therapies are currently lacking. Recently, the amniotic membrane (AM) has shown promising results in wound management. Here, the potential role of AM on endothelial cells isolated from the umbilical cord vein of gestational diabetes-affected women (GD-HUVECs), has been investigated. Indeed, GD-HUVECs in vivo exposed to chronic hyperglycemia during pregnancy compared to control cells (C-HUVECs) have shown molecular modifications of cellular homeostasis ultimately impacting oxidative and nitro-oxidative stress, inflammatory phenotype, nitric oxide (NO) synthesis, and bioavailability, thus representing a useful model for studying the mechanisms potentially supporting the role of AM in chronic non-healing wounds. In this study, the anti-inflammatory properties of AM have been assessed using a monocyte endothelium interaction assay in cells pre-stimulated with tumor necrosis factor-α (TNF-α) and through vascular adhesion molecule expression and membrane exposure, together with the AM impact on the nuclear factor kappa-light-chain-enhancer of activated B cell (NF-kB) pathway and NO bioavailability. Moreover, GD-HUVEC migration and tube formation ability were evaluated in the presence of AM. The results showed that AM significantly reduced TNF-α-stimulated monocyte–endothelium interaction and the membrane exposure of the endothelial vascular and intracellular adhesion molecules (VCAM-1 and ICAM-1, respectively) in both C- and GD-HUVECs. Strikingly, AM treatment significantly improved vessel formation in GD-HUVECs and cell migration in both C- and GD-HUVECs. These collective results suggest that AM positively affects various critical pathways in inflammation and angiogenesis, thus providing further validation for ongoing clinical trials in diabetic foot ulcers.

O-07 Super-resolution genetics in haploid stem cells: decoding Hedgehog signaling in development and cancer

Giulio Di Minin[1]

[1] Institute of Molecular Health Sciences, Department of Biology, Swiss Federal Institute of Technology ETH Hönggerberg, Zurich, Switzerland

Advances in the generation of haploid embryonic stem cells (ESCs), capable of self-renewal and differentiation, have laid the groundwork for numerous biomedical applications in developmental biology and reproductive medicine. Haploid cells possess one copy of each gene, facilitating the generation of loss-of-function mutations in a single step and allowing the development of efficient functional genomic strategies. Our group was the first to derive mammalian haploid embryonic stem cells and profoundly contributed to explore their developmental potential. In these years, the methodology reached a maturity that allows us to challenge complex phenotypes with a sensitivity unreachable with any other screening approach. Using this cellular system, we explored the role of the Hedgehog (HH) pathway during the development and diseases. Hedgehog (HH) signaling is important for embryonic patterning and stem cell differentiation. The G-protein coupled receptor Smoothened (SMO) is the key HH signal transducer modulating both transcription-dependent and independent responses. We show that SMO protects naive mouse ESCs from dissociation-induced cell death. We exploited this SMO dependency to perform a genetic screen in haploid ESCs where we identify the Golgi proteins TMED2 and TMED10 as factors for SMO regulation. Super-resolution microscopy shows that SMO is normally retained in the ER and Golgi compartments and we demonstrate that TMED2 binds to SMO preventing localization to the plasma membrane. We demonstrate the physiologic relevance of this interaction in neural differentiation, where TMED2 functions to repress HH signal strength. Identification of TMED2 as a binder and upstream regulator of SMO opens the way for unraveling the events in the ER-Golgi leading to HH signaling activation.

CELL FREE THERAPIES SESSION

O-08 Camouflaging synthetic nanomaterials with MSC extracellular vesicle membreanes

Paolo Bergese[1]
[1] Department of Molecular and Translational Medicine, Università degli Studi di Brescia, Italy
IRIB - Institute for Research and Biomedical Innovation of CNR, Palermo Italy
CSGI - Italian Center for Colloid and Interface Science, Firenze, Italy

MSC-derived extracellular vesicles (MSCEVs) can reduce inflammation, promote healing, and improve organ function, thereby providing a potential “cell-free” therapy. EV innate circulation and targeting performances take origin in the unique cell-tailored composition and structure of their membrane, which is to date inaccessible to synthetic mimics. The talk will (friendly) introduce the synthetic-biogenic (hybrid) nanotechnology we developed to recapitulate such key natural biomimetic functions to synthetic nanomaterials and nanodevices, which is based on dressing them with a wetsuit made of MSCEV (and other extracellular vesicle) membrane fabric.
For further details and updates https://www.bowproject.eu/.

O-09 The role of glucocorticoids in the regulation of cardiomyocyte regenerative plasticity

Gabriele D’Uva [1,2]
[1] Department of Medical and Surgical Sciences, University of Bologna, Bologna, Italy
[2] IRCCS Azienda Ospedaliero-Universitaria di Bologna, Bologna, Italy

Severe cardiac injuries, like those resulting from myocardial infarction, can lead to substantial cardiomyocyte loss and the formation of fibrotic scar tissue in mammals. This, combined with the adult mammalian heart's inability to naturally regenerate, often culminates in heart failure. Hence, developing therapeutic strategies for cardiac regeneration is clinically important.
Some lower vertebrates, such as zebrafish and amphibians, possess an innate ability to regenerate their hearts throughout their lives. Recent studies have also revealed the remarkable regenerative potential of mammals

during prenatal and early postnatal development. Notably, dedifferentiation and proliferation of existing cardiomyocytes, rather than stem cell differentiation is the primary cellular mechanisms through which lower vertebrates and neonatal mammals spontaneously regenerate injured hearts.

However, the regenerative capacity of the mammalian heart significantly diminishes shortly after birth, as most cardiomyocytes undergo further maturation and exit the cell cycle. Although a gradual rate of cardiomyocyte renewal has been observed in adult mammals, including rodents and humans, it remains insufficient to support regeneration. Recent research has elucidated how adaptations from intrauterine to extrauterine life, starting at birth and continuing during the early postnatal period, contribute to the loss of cardiac regenerative potential in mammals. These findings have paved the way for innovative regenerative approaches aimed at reactivating the endogenous cardiac regenerative capacity through the dedifferentiation and proliferation of existing cardiomyocytes.

Through comprehensive analysis of the early postnatal developmental timeframe, we have recently identified glucocorticoids as pivotal players in cardiac regenerative plasticity. Endogenous glucocorticoids activate the glucocorticoid receptor (GR), promoting cardiomyocyte maturation while concurrently reducing cardiomyocyte proliferation. Importantly, genetic deletion of GR or its pharmacological inhibition following myocardial infarction in juvenile and adult mice facilitates cardiomyocyte cell cycle re-entry and division. This process ultimately fosters cardiac muscle regeneration while diminishing scar formation. Thus, GR activation limits heart regeneration, offering potential avenues for therapeutic interventions targeting this receptor.

O-10 Mesenchymal stromal cell secretome for regenerative medicine: modulation of soluble factors and miRNAs in extracellular vesicles under different culture conditions for joint pathologies

Enrico Ragni[1], Alessandra Colombini[1], Michela Maria Taiana[1], Paola De Luca[1], Simona Piccolo[1], Giulio Grieco[1], Vincenzo Raffo[1], Laura de Girolamo[1]

[1] IRCCS Ospedale Galeazzi - Sant'Ambrogio, Laboratorio di Biotecnologie Applicate all'Ortopedia, Via Cristina Belgioioso 173, 20157 Milano, Italy

OBJECTIVE

In regenerative medicine approaches related to orthopaedic conditions, mesenchymal stromal cells (MSCs) have shown positive outcomes due to the secretion of therapeutic factors, both free and conveyed within extracellular vesicles (EVs). MSC-derived factors can be modulated by both culture and environmental conditions but a complete fingerprint is still missing in the context of orthopaedic applications. Thus, the aim of this work is to characterize adipose-derived MSC (ASCs)-secreted factors and EV-miRNAs and their modulation after high levels of IFNγ preconditioning or low levels of inflammation, mimicking the synovial fluid of patients with osteoarthritis (OA). Furthermore, the ability of ASC-EVs to penetrate cartilage explants is evaluated.

MATERIALS AND METHODS

ASCs were isolated from four donors and cultured with and without IFNγ (1 ng/ml) or TNFα (5 pg/ml) + IL1β (10 pg/ml) + IFNγ (40 pg/ml) mimicking OA synovial fluid concentration. First, 200 secreted factors were analysed by ELISA. Second, 754 miRNAs were studied by high-throughput qRT-PCR screening in ultracentrifuge-purified EVs. Bioinformatics tools were used to predict the modulatory effect of the identified molecules on pathological cartilage and synovial macrophages. Immunofluorescence and time-lapse coherent anti-Stokes Raman scattering, second harmonic generation and two-photon excited fluorescence were used to follow and quantify the incorporation of fluorescent EVs into cartilage explants.

RESULTS

More than 50 cytokines/chemokines and more than 200 EV-miRNAs could be identified. The majority of molecules are involved in the remodelling of the extracellular matrix and in the homeostasis of inflammatory cells including macrophages, promoting their shift towards an M2 phenotype. Inflammatory priming and synovial fluid-like conditions were able to modulate the secretome ability to stimulate healing and reduce

inflammation. Finally, the penetration of EVs in cartilage explants was monitored as a rapid process, starting within minutes of administration and reaching a depth of 30-40 μm after 5 hours and the plateau at 16 hours.

CONCLUSIONS

The secretome obtained from ASCs can be considered as the main vehicle through which the mesenchymal stromal cells transfer their regenerative and anti-inflammatory potential and the preconditioning in an inflammatory environment can further modulate their therapeutic capacities for future applications in the field of joint pathologies such as OA.

TIPS AND TRICKS OF RESEARCH VALORIZATION SESSION

O-11- From research to enterprise: Materias as a model

L. Nicolais[1], C. Meglio[1], A. Cammarano[1], A. Cummaro[1]
[1] M.E. Mercurio Materias s.r.l. – Naples

The road from a discovery stemming from basic research to a commercial product or process is lengthy and is often fraught with significant challenges. In many cases, both innovators and investors frequently refer to a "funding gap" or a "Death Valley" that exists between the conception of novel ideas and the successful commercialization of these innovations.
One significant challenge in this journey is the fact that researchers are not necessary innovators, and as such, they may lack the expertise needed to transform research findings into viable commercial products or processes.
To promote an entrepreneurial culture, facilitate the dissemination of scientific outcomes, and provide critical support to scientists as they traverse the path toward commercializing their research results, Horizon Europe has developed a European Innovation Council. This approach aims to position Europe as a leader in technology transfer and in the generation of groundbreaking innovations that create new markets.
Despite the persistent innovation and competitiveness seen in Italy's research community, the full industrial potential of Italian scientific research remains unrealized. This deficiency can be traced back to inadequate research investments, a fragile venture capital landscape, and a lack of proficiency in efficient technology transfer.
The Materias business model was developed to overcome this deficiency. It aims to bridge the academic research and industrial firms in the field of advanced materials and to promote new business opportunities by exploiting research results.

O-12 - SCIENTIFICA IS THE VENTURE CAPITAL FOR SCIENCE

Lucilla Mazzeo (Rome)

Scientifica Venture Capital is an investment holding company that selects high-tech projects and startups.

It was founded in November 2021 and has already funded 8 start-ups, with an investment goal of 40 startups in the first 4 years.
It invests in advanced manufacturing, advanced materials, artificial intelligence and quantum technologies in the pre-seed, seed, and early-stage phases.
Scientifica Venture Capital, with offices in Rome and London, provides its portfolio start-ups with a dual asset: financial support and access to laboratories and necessary equipment to develop prototypes.
Research and pre-industrial validation activities can be conducted either within the Scientifica Lab in L'Aquila - with over 1400 square metres of space for chemistry, biochemistry, electronics and 3D printing - or in the laboratories of the Scientifica Lab Approved network, which connects excellent research facilities distributed throughout Italy.
These laboratories generate high levels of knowledge that startups can incorporate as technological innovations in their products or services, enhancing their competitiveness in

terms of business.
The simultaneous ability to support innovative companies in the tech transfer process and market positioning makes Scientifica an exceptionally innovative Venture Capital firm, capable of promoting Italian research excellence on the international level and emphasizing its importance as an essential driver of economic competitiveness.

THE TECHNOLOGY TRANSFER MODEL IS BASED ON 3 PILLARS:
1.Investments
2.Facilities
3.People

Investments

Scientifica Venture Capital funds innovative ideas and start-ups with investment tickets ranging from €50,000 to €500,000. The investment is made only after a positive outcome of the due diligence process conducted on the project under examination.
The investment financial instruments used by Scientifica Venture Capital include:

- Capital Increase
- Convertible Note

Facilities

Scientifica Lab Approved
This is an extensive network of top-tier laboratories distributed throughout Italy, facilities that Scientifica Venture Capital has interconnected to create a "widespread" laboratory system.
Thanks to the Lab Approved network, start-ups that receive funding are ensured proximity to laboratories equipped with cutting-edge machinery.

People

The team at Scientifica Venture Capital consists of professionals with multi-sectoral expertise who work synergistically to transform the start-up into a successful company.
There are the 3 main areas:

- Investment (area)
- R&D (area)
- Marketing and Communication (area)

O13- What you need to know for an efficient technology transfer

Manuela Monti
IRCCS Istituto Romagnolo per lo Studio dei Tumori (IRST) "Dino Amadori", Meldola, Italy

Advanced Therapies (ATMP) represent the new frontier for the treatment of several rare genetic and onco-hematological pathologies, as well as options for the treatment of chronic-degenerative pathologies that are currently orphaned by therapies. Over the last decade, Italy's contribution to this sector has been significant: of the 17 advanced therapies authorized so far in the European Union, 4 are the result of Italian academic research. On the way to reaching clinical practice, some crucial aspects must be taken into consideration such as pre-clinical research, clinical research, GMP production, therapy feasibility and sustainability. The aim of basic research must therefore not only be aimed to the scientific publication, but also to the development of new therapies. To this scope, even academic studies should be designed from the very early stage according to regulatory authorities requirements, to avoid incurring delays and to reduce the risk of failure as much as possible. Furthermore, quality of data from clinical trials must be of high standards. It is therefore crucial to put in place effective strategies between academic institutions and other stakeholders, with the aim to speed up and optimize the regulatory framework and to identify the major critical issues linked to the development of ATMP.

O-14 Ralf Hass[1] - Potential use of MSC for translational clinical treatments

Ralf Hass[1]

[1] Biochemistry and Tumor Biology Laboratory, Department of Obstetrics and Gynecology, Hannover Medical School, 30625 Hannover, Germany

Human mesenchymal stroma-/stem-like cells (MSC) represent a heterogeneous population of multipotent progenitor cells that are activated at damaged tissue sites, initiate repair processes, and regulate tissue homeostasis. A variety of different properties characterize MSC including (plastic) adherence, expression of distinct surface marker proteins and differentiation potential at least along osteogenic, adipogenic, and chondrogenic lineages.
MSC can interact with adjacent damaged or neoplastic cells by direct and/or indirect mechanisms. Direct interactions include the exchange of cellular parts or even complete cell fusion e.g. leading to new types of cancer cells in case of close MSC:cancer cell interactions followed by a merger. During indirect interactions, MSC can affect even distant target cells via release of soluble factors or extracellular vesicles (EVs) such as exosomes.
Beside the use of MSC as a transplantable cell system, MSC-mediated release of EVs as cell-free vesicular products including microvesicles or exosomes are gaining increased attention for potential clinical applications. Exosomes represent small double membraned particles with approximately 20 to 200 nm in diameter containing a distinct set of tetraspanins (CD9, CD63, and CD81) as marker proteins and carry a variety of regulatory RNAs such as microRNAs (miRs).
In damaged tissues MSC-derived exosomes can induce repair activities, promote regenerative potential, and can also serve as highly effective senotherapeutics.
As a diagnostic tool, exosomes can unravel disease-specific patterns of biomolecules such as the early appearance and detection of certain proteins or miRs during tumor development. Moreover, due to the emerging tropism of MSC towards tumors, MSC-derived exosomes can be used in a tumor-therapeutic approach as a vehicle to selectively deliver anti-tumor cargo to cancer cells. In particular, taxol-loaded MSC-derived exosomes can target cancer cells in a variety of different tumor entities including lung, breast, ovarian, colon and neuronal cancers both, in vitro and in vivo. Thus, MSC-derived exosomes containing anti-tumor cargo may provide a promising tool for a targeted tumor-therapeutic approach with biocompatible characteristics of a distinct miR pattern.
However, there is still a need to standardize various parameters including biosafety, pharmacovigilance, pharmacokinetic, and biodistribution among others for reproducible clinical applications of MSC-derived exosomes.

O-15 Phase I clinical trial on pleural mesothelioma using neoadjuvant local administration of paclitaxel loaded mesenchymal stromal cells (PACLIMES trial): rationale and design

Giulia M. Stella[1], Patrizia Comoli[1], Daniela Lisini[2], Augusto Pessina[3], Catherine Klersy[1], Paolo Pedrazzoli[1], Angelo G. Corsico[1]
[1] University & IRCCS Fondazione Policlinico S.Matteo, 27100 Pavia, Italy
[2] Cell Factory Unit, IRCCS Ist. C. Besta, 20133 Milano, Italy
[3] University of Milan , 20122 Milan, Italy

Pleural mesothelioma (PM) is a rare and aggressive neoplasm that originates from the pleural mesothelium and whose onset is mainly linked to exposure to asbestos. To date, PM cannot be attacked with truly effective therapies and the average patient survival is about 12-15 months from diagnosis. There is an urgent need for more effective therapeutic approaches. The rationale of this study is based on the use of mesenchymal stromal cells (MSCs) as a vehicle for chemotherapy drugs to be injected directly into the pathological site, as the pleural cavity. The latter, despite the physiological draining capacity of the membrane layer, represents an ideal space for the local treatment in case of cancer. The study involves the use of a conventional chemotherapeutic drug, Paclitaxel (PTX) which is widely used in the treatment of different types of solid tumors, among which PM although some limitations related to pharmacokinetic aspects. The use of PTX-loaded MSCs to treat PM should provide several potential advantages over the systemically administered drug as reduced toxicity and increased concentration of active drug in the tumor surrounding context. The primary objective of the study

is to evaluate safety and toxicity of local administration of Paclimes in chemonaive patients, candidates to pleurectomy. The secondary objective is to find the effective Paclimes dose for subsequent phase II studies and to observe and record the antitumor activity. The route of drug administration is endopleural. In the case of patients affected by PM with concomitant pleural effusion, endopleural drainage will be performed for fluid evacuation. Subsequently, a volume of 3-5 ml containing 100-135x106 PTX-carrying MSCs will be infused through the tube. It will be temporarily clamped, and the patient will be asked to change his decubitus periodically. This procedure will be repeated in the following 2 days. At the end of the third day, talcum will be infused. In the case of patients with suspected PM with pleural effusion, medical thoracoscopy will be performed, biopsies performed and at the end of the procedure a drainage tube will be left in the cable. The times for pathological confirmation will not exceed 5 days. Paclimes will then be infused as described. In any case, endopleural drainage will be removed after 2/3 days. Then the patients will follow the conventional therapeutic program and will be directed to pleurectomy within 3-4 weeks and subsequent standard chemotherapy.

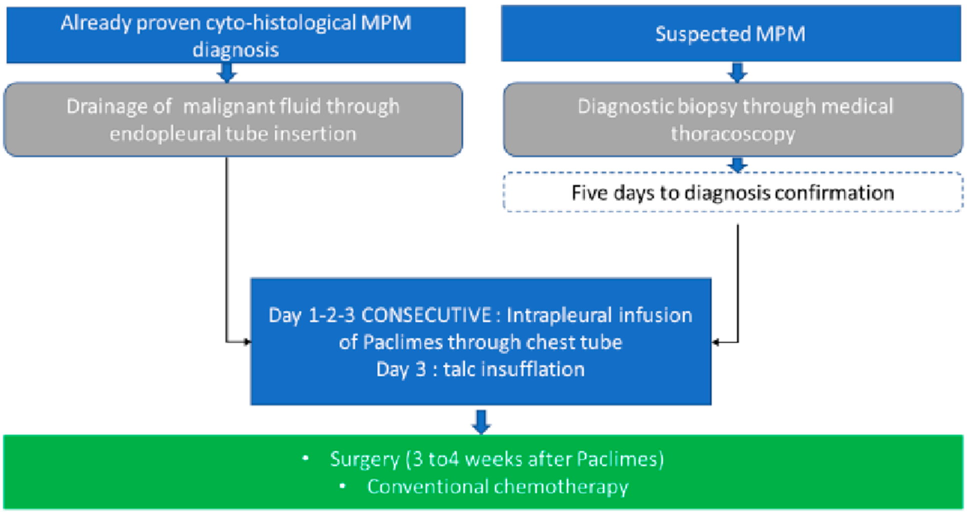

O-16 (YIA) Harnessing mesenchymal stem cells-Mediated Tumor Microenvironment Perturbation for Enhanced Chemiosensitivity: proposing a preclinical study as a step towards clinical implementation of a promising hypothesis

Benedetta Ferrara[1], Smeralda Rapisarda[1], Paolo Camisa[1], Antonio Citro[1], Chiara Gnasso[2], Anna Paola Andolfo[3], Denise Drago[3], Andrea Annoni[4], Lorenzo Piemonti[1]
[1] Diabetes Research Institute, IRCCS San Raffaele, Milano
[2] Experimental Imaging Centre, IRCCS San Raffaele, Milano
[3] Proteomics and Metabolomics Facility, IRCCS San Raffaele, Milano
[4] Immune Core, San Raffaele Telethon Institute for Gene Therapy, IRCCS San Raffaele, Milano

OBJECTIVE

Pancreatic ductal adenocarcinoma (PDAC) is notorious for its high resistance to conventional therapies, primarily due to the limited penetration of drugs caused by the dense fibrotic stroma. Overcoming this physical barrier is essential to achieve effective therapeutic outcomes. Mesenchymal stem cells (MSCs) have emerged as a promising drug-delivery tool, leveraging their ability to incorporate drugs and selectively target tumors. The objective of this project was to investigate the effects of MSC-based therapy using nab-paclitaxel (n-PTX) in preclinical models of metastatic PDAC.

MATERIALS AND METHODS

Liver metastases were induced by injecting the K8484 cell line into the portal vein of C57BL/6N male mice. Firstly, tumor bearing mice underwent intravenous (i.v.) or intraportal injection of syngeneic bone marrow luciferase-transduced MSCs (LUC+MSCs) to study their biodistribution by in vivo imaging system (IVIS). A further evaluation was performed by investigating GFP+MSCs distribution in liver parenchyma using confocal microscopy. MSCs were then loaded with n-PTX (500 nM) and administered to mice with established tumors by a second intraportal injection at day 20 after tumor induction. The metastatic burden was monitored using MRI imaging, and toxicity was assessed through biochemical analysis.

RESULTS

Results obtained on MSCs biodistribution revealed a high and prolonged cell accumulation in the liver after intraportal injection, while i.v. injected MSCs accumulated in the lung, making intraportal injection preferable. MSCs demonstrated to distribute homogeneously in the liver parenchyma with higher accumulation towards blood vessels. N-PTX-loaded MSCs reported a significantly higher reduction of the metastatic burden in comparison to control mice (treated with not-loaded MSCs or saline). Mice treated with free n-PTX displayed a similar reduction curve but this regimen impaired animal survival because of intrinsic toxicity. Biochemical analysis did not report signs of evident and prolonged toxicity after n-PTX-loaded MSCs infusion.

CONCLUSIONS

The development of a strategy involving MSCs as carriers of n-PTX to treat metastatic PDAC is promising. This system allowed to lower the drug dose thus reducing n-PTX toxicity and specifically targeting the tumor site reporting an effective reduction of the metastatic burden. By defining efficacy and toxicity of this approach, this study provides valuable insights for potential translation into clinical trials.

O-17 - (YIA) Preparation and characterization of extracellular vesicles from adipose tissue-derived mesenchymal stromal cells loaded with paclitaxel for clinical use in the treatment of mesothelioma patients

Eleonora Spampinato[1], Angela Marcianti[1], Sara Nava[1], Simona Frigerio[1], Simona Pogliani[1], Giulia Maria Stella[2], Guido Maronati[3], Paola Gagni[4], Federico Cazzaniga[5], Angelo Guido Corsico[2], Daniela Lisini[1]

[1] Cell Therapy Production Unit, Scientific Direction, IRCCS Neurologic Institute C. Besta Foundation, 20133 Milan

[2] Department of Medical Sciences and Infective Diseases, Unit of Respiratory Diseases, IRCCS Policlinico San Matteo Foundation and University of Pavia Medical School, 27100, Pavia

[3] Synlab CAM Polidiagnostico, 20900 Monza

[4] Scitec CNR, 20133 Milan

[5] Division of Neurology 5 and Neuropathology, IRCCS Neurologic Institut C. Besta Foundation, 20133 Milan

OBJECTIVE

Extracellular vesicles from Mesenchymal Stromal Cells (MSC-EV) loaded with Paclitaxel can represent a new promising approach for the treatment of oncological diseases, as malignant pleural mesothelioma, but a standardized Good Manufacturing Practice (GMP)-compliant preparation and conservation procedure is lacking. This issue is crucial to use the product as drug. The aim of this project was to set up a standardized protocol for the preparation of MSC-EV loaded with PTX (EV-PTX) from adipose tissue lipoaspirates of healthy donors, investigating also the possibility of their conservation.

MATERIALS AND METHODS

MSC obtained from 11 donors were expanded under GMP conditions for a maximum of 3 passages. To prepare EV-PTX, PTX (10ug/ml) was added to the medium for 20 hours before supernatant collection. Supernatant ultracentrifugation was used to isolate EV/EV-PTX. Products preserved at -80°C in both 0.9%NaCl and 0.9%NaCl supplemented with 1% dimethylsulfoxide (DMSO) were analyzed to evaluate: identity (flow cytometry), concentration/size/morphology (Nanoparticle Tracking Analysis, NTA/Transmission Electron Microscopy, TEM) and protein content (microBCA).

RESULTS

EV preserved in 0.9%NaCl and 0.9%NaCl/1%DMSO were similar for concentration (1,12E+10±2,95E+09 vs 1,43E+10±7.78E+09 particles/ml), size distribution (171,46±32,93 vs 156,70±18,49 nm) and protein content (28,38±9,14 vs 33,38±7,65 ng/ml). All data are reported as mean±SD. Flow cytometry analysis showed high expression of EV markers CD9, CD81 and CD63 and MSC markers CD29, CD44, CD146 and CD105 in both conservation mode. However EV integrity/morphology seems to be better preserved using 0.9%NaCl/1%DMSO, as shown by TEM and NTA istograms. Indeed EV-PTX were conserved in 0.9%NaCl/1%DMSO.

EV-PTX showed a size distribution of 206,31±9,96 nm (mean±SD), higher than that of unloaded EV, but a similar concentration (1.09E+10±5,13E+09 particle/ml) and protein content (23,43±6,80ng/ml). As EV, EV-PTX displayed high expression of both EV and MSC markers.

Analysis of the drug content, anti-tumor efficiency and the scalability of manufacture method involving a bioreactor are in progress.

CONCLUSIONS

These data show that our EV-PTX preparation protocol is successful and permit to maintain product integrity/identity. Conservation in 0.9%NaCl/1%DMSO seems to be better than NaCl alone. These results indicate a possible future clinical use of EV-PTX as anti-tumor drug to improve treatment of mesothelioma patients

O-18 Activated Mesenchymal Stromal Cell Therapy for Management of Drug Resistant Infections

Steven Dow, DVM, PhD.
Director, Immunotherapy Research Laboratory, Department of Clinical Sciences, College of Veterinary Medicine and Biomedical Sciences, Colorado State University

As an adjunct to antimicrobial therapy for management of drug resistant infections, we have developed a cell therapy approach to help eliminate highly drug-resistant bacteria, while also stimulating wound healing. When mesenchymal stromal cells derived from bone marrow or adipose tissues undergo an activation process after stimulation with certain Toll-like receptor agonists, they begin to migrate more efficiently to wounds, to secrete large amounts of antimicrobial peptides, and to release cytokines that activate the innate immune system, particularly monocytes and neutrophils. When co-administered with antibiotic therapy, activated cell therapy can clear very resistant and long-standing infections. In this presentation we will discuss the stromal cell activation process, the mechanism of action based on rodent infection models, and results of clinical studies in dogs with chronic wound infections and horses with septic arthritis. The clinical applications of the activated cell therapy technology will be discussed, including situations where the approach is most and least likely to work, cell sourcing, antibiotic selection, and the potential for trans-species activated cell therapy.

O-19- CALIN Veterinary Regenerative Network: Scientific and Clinical Interface for Standardization of Cell Therapy in Animals

Ana Ivanovska[1], Frank Barry[1]
[1] Regenerative Medicine Institute (REMEDI), University of Galway, Ireland

The Celtic Advanced Life Science Innovation Network (CALIN) was established to sustain university-industry research and assist enterprises in Ireland and Wales throughout the research and development process.

In line with this, there is a real need to foster translational collaborations to tackle current challenges existing in the area of animal cell therapies. To address this necessity, the CALIN Veterinary Regenerative Network was established as a multidisciplinary platform of human and veterinary stem cell scientists in collaboration with veterinary orthopaedic surgeons interested in adopting cell therapies for osteoarthritis in their clinical practices.

Based on the One Health – One Medicine approach, current standards for human stem cell isolation and characterisation were applied for the generation of cell banks of equine umbilical cord-blood and canine adipose-derived mesenchymal stem cells (MSCs). Thorough review of the published literature identified lack of conclusive evidence based on the high variability in current clinical trials. Survey conducted within the veterinary community highlighted cost, availability and efficacy as main factors limiting the wide-spread use of animal cell therapies. For educational purposes 8 webinars were organised for the global veterinary community addressing a variety of relevant disease topics delivered by collaborators from veterinary schools in Europe and USA.

In summary, interdisciplinary collaborations are fundamental for the further development of the cell therapies in human and veterinary medicine. Clinically oriented laboratory-based findings should be used as a starting point and further tested in gold-standard clinical trials to generate data on safety, efficacy and long-term effects needed for a wider application in the clinical practice.

O-20 (YIA) MESENCHYMAL STROMAL CELLS DERIVED FROM CANINE ADIPOSE TISSUE: EVALUATION OF THE SHIPPING VEHICLES USED FOR CLINICAL ADMINISTRATION

Valentina Andreoli[1], **Priscilla Berni[1],** Virna Conti[1], Roberto Ramoni[1], Giuseppina Basini[1] and Stefano Grolli[1]
[1] Department of Veterinary Sciences, University of Parma, 43100, Parma, Italy

OBJECTIVE

Mesenchymal Stromal Cells (MSCs) based therapies are rapidly gaining interest in veterinary medicine. The use of cellular therapy represents a new challenge for practitioners and requires precise coordination between the facility in charge of the preparation/shipment of the cells and the clinic. The choice of a safe method and vehicle for MSCs delivery is a crucial aspect and is still controversial. Cryopreservation is supposed to be the best method. However, potentially toxic cryoprotectants and xenobiotic products make the direct administration of cells impracticable. Alternatively, the cells may be resuspended in a ready-to-use vehicle and shipped to the clinic. The present study aims to evaluate the short- (two, four hours) and long-term (twenty-four hours) biological effects of some of the most common vehicles used for the clinical application of canine adipose tissue-derived MSCs (Ad-MSCs).

MATERIALS AND METHODS

Ad-MSCs were stored at low (1x106/ml) and high (4x106/ml) densities for two, four, and twenty-four hours in different vehicles, such as the releasate derived from autologous Platelet Poor Plasma (PPP) and Platelet Rich Plasma (PRP), the physiologic saline and the ringer lactate solution (RLS). Cells were compared regarding mortality, metabolic activity, and replicative capacity. Furthermore, after twenty-four hours of storage, the antioxidant activity and expression analysis of a panel of genes involved in MSCs' biological features were investigated.

RESULTS

Regarding the short-term period, the mortality rate was not affected by the different environments of storage, always remaining below 10%, while a lower metabolic activity was observed in cells stored in physiological saline. The long-term evaluation was performed choosing RLS and PRP as tested solutions. No differences were found at low density, while a higher mortality rate and lower metabolic activity were observed for RLS at high density. Finally, the antioxidant activity test didn't show any difference, while for the gene expression analysis, significant changes were observed only for STC-1 (higher in RLS) and COX-2 (lower in PRP).

CONCLUSIONS

The results showed that all the different vehicles preserved cell viability and replication following the short-term storage, while the long-term storage is influenced by the cell concentration and type of vehicle. In detail, nutrient-rich vehicles (PPP and PRP) seem to be better suited to preserve cell vitality in these conditions.

O-21 Regenerative ophthalmology

Diego Ponzin and Stefano Ferrari[1]-
[1] Fondazione Banca degli Occhi del Veneto, Venice

INTRODUCTION
Regenerative medicine is aimed at improving / restoring the function of organs and tissues damaged by diseases, traumas, ageing, by slowing down the progression, or reverting the course of the degeneration. It covers the fields of organ and tissue transplantation, cell therapy, and gene therapy and, with the exception of organ transplantation, represents a possible therapeutic option for eye diseases as well. We may refer to this particular field as regenerative ophthalmology.
Over the last decades, significant clinical and scientific advancements have led to new approaches to severe cornea, ocular surface and retinal diseases.

Regenerative ophthalmology
Diseases affecting the cornea and the ocular surface are a major cause of blindness worldwide, together with cataract, glaucoma and age-related macular degeneration. While penetrating keratoplasty (PK) remains the most common procedure for cornea transplantation, improvements in microsurgical techniques and new devices have led to increasing numbers of selective (lamellar) keratoplasty procedures: the anterior lamellar keratoplasty, replacing the anterior stroma, and the posterior lamellar keratoplasty, also known as endothelial keratoplasty (EK), which involves the replacement of deep stromal and endothelial layers. A major advantage of endothelial over penetrating keratoplasty is that it is a closed eye procedure, thus minimising the risks of complications.
This is having an impact on the eye banks, as the corneal tissue preparation in the laboratory reduces the manipulation of the donor tissue at the time of surgery. In addition, it might broaden the donor cornea pool by enabling tissues that cannot be used for PK to be used for anterior or endothelial transplantation.
Patients with limbal stem cell deficiency (LSCD) are poor candidates for conventional keratoplasty. In pathologies or injuries leading to LSCD, the success of conventional corneal transplantation is hindered by the deficiency of stem cells, which allows for conjunctival cell ingrowth, neovascularization and inflammation, resulting in corneal graft failure.
The amniotic membrane (AM) derives from the innermost layer of the placenta and is composed of a basement membrane and an avascular stromal matrix. AM transplantation reduces scars during ocular surface reconstruction, sectorial LSCD and glaucoma surgery. Besides the lack of immunogenicity, AM facilitates epithelialization in persistent corneal epithelial defects, suppresses the expression of inflammatory cytokines, sequesters inflammatory cells and contains various forms of protease inhibitors. Finally, the AM stromal matrix is known to suppress TGF signaling, proliferation and myo-fibroblastic differentiation of corneal, limbal and conjunctival fibroblasts. Other newer applications of AM are its use:
- as scaffold for propagation and transplantation of limbal stem cells onto the eyes with LSCD;
- to expand conjunctival cells for the regeneration of the conjunctival epithelium;
- as homogenate to be applied as an eye drop in severe dry eye;
- as scaffold for spontaneous differentiation and propagation, in feeder-free conditions, of human embryonic stem cell line-derived Retinal Pigment Epithelium, and subsequent implantation in the subretinal space of eye with age-related macular degeneration.

The most important breakthrough in managing ocular surface failure is related to the identification of the location and function of limbal stem cells. In 1989, Kenyon and Tseng pioneered the clinical application of the limbal stem cell theory by transplanting grafts of bulbar conjunctiva and limbus harvested from the normal fellow eye to manage cases with unilateral LSCD. In cases of bilateral LSCD, limbal allografts were performed using tissue from a living relative, or deceased, donors. Whether autograft or allograft, outgrowth of epithelial cells from transplanted sectors of limbal tissue onto the affected eye resulted in repopulation of the corneal surface with corneal epithelial cells. In the last 20 years all these pioneering treatments have been further refined, and also the techniques for ex vivo expansion of autologous limbal stem cells have been investigated. Some studies used the explant culture, in which harvested limbal tissue (autologous or allogeneic) is placed directly onto amniotic membrane (used as a surrogate environmental stem cell niche) and the limbal epithelial

cells migrate out of the biopsy and proliferate to form an epithelial sheet. Alternatively, limbal stem cells are isolated from the biopsy using an enzymatic treatment and seeded onto an amniotic membrane or a feeder-layer of lethally-irradiated murine 3T3-J2 fibroblasts. To our knowledge, only limbal stem cells cultured onto 3T3-J2 feeder-layers and transferred to the diseased eye using fibrin as carrier were shown to maintain undifferentiated phenotypes and ability to restore the stem cell population.

Patients with bilateral total LSCD require transplantation of donor-derived limbal stem cells, with the prospect of life-long immunosuppressive treatments and the risks associated. Therefore, alternative / additional cell sources are necessary. The conjunctival epithelium may also be damaged by a variety of ocular disorders, such as chemical burns and Steven-Johnson syndrome. The use of bioengineered conjunctival equivalents would therefore be an alternative for disorders involving the conjunctiva, and it would avoid harvesting conjunctival autografts and causing iatrogenic injury to the remaining ocular surface. Better protocols to isolate, characterise and expand human conjunctival goblet cells should be identified, thereby opening new perspectives for the treatment of disorders caused by conjunctival goblet cell deficiency.

Eye diseases causing dysfunction or a low density of human corneal endothelial cells (HCECs) require transplantation of full- or partial-thickness corneas containing a healthy endothelium. A desirable alternative would be to cultivate HCECs in vitro thus regenerating a sheet that could be grafted onto patients affected by a diseased corneal endothelium. The main obstacle is that HCECs do not normally replicate in vivo and are arrested in the G1-phase of the cell cycle. Despite this, several studies have evaluated protocols to isolate and expand HCECs in vitro starting from donor corneas. In the future, it might be possible to expand HCECs into autologous human endothelium monolayers from biopsy samples. Potential future strategies might be based on the genetic modification of HCECs, for endothelial disorders such as Fuchs endothelial corneal dystrophy and endothelial decompensation in bullous keratopathy and graft failure.

Age-related macular degeneration is the leading cause of blindness among the elderly in Western countries, and can be classified into non-exudative and exudative form. Both types result in photoreceptor degeneration, due to the abnormal alteration of the underlying Bruch’s membrane and retinal pigment epithelium, whose function is irreversibly damaged. In the last decade, cell therapy products designed for retinal pigment epithelium replacement have been shown to rescue photoreceptors and prevent visual loss in preclinical models of macular degeneration. Previous subretinal transplantation with fetal or adult RPE cells has demonstrated limited long-term success.

CONCLUSIONS

Many challenges are lying ahead in the field of regenerative ophthalmology that could further change ophthalmic care.

Stem cell-based therapies have been classified as medicinal products by the European Union (EU) and are therefore regulated according to the manufacture of biological medicinal products for human use. Groups providing cellular therapies within the EU will have to comply with EU laws, which require that grafts are only produced by accredited tissue banks under the defined conditions of good manufacturing practice (GMP).

Many of the studies conducted so far, and describing innovative products or applications, are just preliminary. Results will therefore have to be confirmed and validated before any of them is used in a clinical setting.

O-22 The HIPGEN phase III study - placental derived stromal cells for muscle regeneration

Tobias Winkler[1], MD, PhD, representing the HIPGEN consortium
[1] Center for Musculoskeletal Surgery, BIH Center for Regenerative Therapies, Julius Wolff Institute
Charité - Universitaetsmedizin Berlin

INTRODUCTION

Despite modern hip arthroplasty techniques, which would allow total weight bearing, mobility is difficult to achieve in hip fracture patients. The second hit of the surgical procedure after the trauma and the additional muscle injury during joint exposition in the frail patients are among the reasons. To date, there is no therapy

to address muscle injuries and surgery related stress. We transferred a therapy with mesenchymal like stromal cells from preclinical experiments to the patient.

METHODS

We established an animal model with a clinically relevant muscle trauma and tested autologous MSC transplantation. Following this, we tested the efficacy of an allogeneic approach using human placenta-derived mesenchymal-like adherent stromal cells (PLX-PAD). We then translated this into clinics by using iatrogenic muscle damage after total hip arthroplasty (THA) as a model and conducted a randomized, double blind, controlled phase I/II study. 20 patients undergoing THA via lateral approach were included and were transplantated with 300x106 (300M), 150x106 (150M) PLX-PAD cells or placebo into the gluteus medius muscle (GM).

RESULTS

Our preclinical experiments demonstrated increased force generation after autologous and allogeneic MSC therapy versus placebo. Phase I/II study patients were followed for 2 years and no relevant AEs were observed. The change of GM strength after 6 months showed a significant increase in the 150M group ($p=0.0067$) compared to placebo, which was accompanied by an increase in muscle volume ($p = 0.004$). Change of strength and volume in the 300M group showed a similar pattern as in the 150M group but not statistically significant. Histology showed faster healing after PLX-PAD. Biomarker studies showed a reduction of postoperative immunological stress due to cell therapy. Based on these results we designed the HIPGEN phase III study. We there treated hip fracture arthroplasty patients (n=240) with an IM injection of 150M PLX cells. Our consortium received funding from the EU Horizon 2020 program (Grant No 779293) and has recently finalized enrolment patients in 20 sites in Germany, England, Denmark, Israel and the US. Partners are among others the universities of Charité, Cattolica del Sacro Cuore Rome, Oxford and Pluri Biotech and the International Osteoporosis Foundation. Patients were followed for function, biomechanics, quality of life, and immunological biomarker studies. We also look into the mechanisms of action by a broad spectrum of in vitro experiments on the effect of PLX cells on muscle cells of healthy donors and HIPGEN patients.

CONCLUSION

Our data showed consistent positive results of MSC therapy for skeletal muscle regeneration in different preclinical application modes and finally in patients, where we currently finalized the first phase III study using a cell therapy in hip fracture patients. This treatment could be an innovative new therapy for patients with traumatic or iatrogenic muscle injuries.

O-23- REGENERATIVE MEDICINE IN ORTHOPAEDIC SURGERY: STATE OF THE ART, CLINICAL EXPERIENCE AND RESEARCH FOR THE FUTURE

Nicholas Crippa Orlandi [1], Nicola Mondanelli[1], Stefano Giannotti[1]
[1] Department of Orthopaedics and Traumatology, University of Siena, Siena (SI)

OBJECTIVE

Orthopaedic surgery can benefit the most from the use of Platelet Rich Plasma and Bone Marrow Concentrate. These are essential biological supplements for a superior-quality orthopaedic surgery. An overview drawn from the experience of the authors' Orthopaedic Clinic is illustrated to highlight the benefits of applying this technology.

MATERIALS AND METHODS

Drawing on the extensive experience gained within the authors' Operating Unit, three areas of application and an experimental study have been selected to prove the benefits deriving from the use of autologous MSC from bone marrow and PRP. The application for bone repair as in the case of cysts and tumour-like lesions, non-union following trauma, bone loss and osteonecrosis, the application for cartilage repair as in the case of

osteochondral lesions and the application for soft tissue repair as in the case of ligaments and tendons reconstruction. The experimental study aims to develop new tissue engineering strategies to be applied in orthopaedic surgery. In the first part of the work, the focus was on the optimization of the isolation and expansion protocol of the hMSCs, taken from the bone marrow and deposited in the Biobank present inside the laboratory which is part of the Network Telethon (TNGB), evaluating the replacement of the FBS (foetal bovine serum) with autologous PRP. Then the focus shifted to the characterization of cells seeded on two types of scaffolds: bovine bone matrix (SmartBone) and lyophilized acellular dermis. The final goal of the work is to develop strategies to increase the osteogenic properties of the support by adding growth factors that enhance cell differentiation on the scaffold.

RESULTS

Our technique of applying stem cells and PRP to cases of complex orthopaedic surgery has shown excellent results both in clinical functional objective and subjective evaluations and in radiographic evaluations. The experimental study, currently underway, is providing excellent results. The data suggest PRP as a valid alternative to FBS, it supports the expansion of mesenchymal stem cells without compromising their capacity since cells loaded on SmartBone differentiate into osteoblasts and produce collagen.

CONCLUSIONS

In our opinion, these biological supplements are and will become more and more a valid tool to provide the surgeon with important help in cases of great complexity and therefore to obtain the tailored care that every patient needs and deserves.

O-24 Biology-driven organ on chips and 3D tissue models to improve disease understanding and accelerate personalized therapies development

S. Scaglione[1,2], E. Palama[2], M. Aiello[1,2]
[1] National Researcher Council (CNR)
[2] React4life S.p.A.

OBJECTIVE

Human disease modeling for both fundamental research and drug testing purposes has traditionally relied on 2D cell cultures under static conditions or in vivo xenografts and genetically engineered animal models. However, the challenges of predictability, reliability, and complete immune compatibility have persisted. In response, novel 3D fully humanized in vitro cancer tissue models have emerged, leveraging cutting-edge technologies such as microphysiological systems (MPS) and 3D cell-laden hydrogels. Notably, a novel Multi-In Vitro Organ (MIVO) MPS platform has been recently employed to cultivate clinically relevant 3D cancer tissues under physiologically relevant conditions, facilitating investigations of immune-tumor cells interplay and the efficacy of anticancer treatments.

MATERIALS AND METHODS

Biologically relevant alginate-based cancer tissues, up to 5 mm in size, were optimized in terms of stiffness and cell density to support the long term cells viability, clusters formation and cytoskeleton reorganization, migration and spreading. Within the MIVO chamber, ovarian, breast and neuroblastoma cell-laden hydrogels were cultured while either traditional drugs or immune cells (Natural Killer cells, NK) where placed in circulation, simulating blood capillary flow in the MPS. The dynamic cell culture conditions were employed to assess tumor cell viability, cytotoxic efficacy of the treatment and immune cells infiltration. A comparison of the cell’s behavior with in vivo data (either animal models or clinical data) was then carried out to successfully validate the model.

RESULTS

A human 3D ovarian model was successfully developed and treated with cisplatin, with similar tumor regression observed in the MIVO platform and in mice, while in contrast static culture displayed unpredictable

chemoresistance due to unreliable drug diffusion within the 3D matrix. Furthermore, a human 3D neuroblastoma cancer model with a relevant immunophenotype was optimized to facilitate a complex tumor/immune cell coculture, serving as a model for immune-oncology screening. Importantly, under dynamic culture conditions, tumor-specific NK cell extravasation was observed, with NK cells infiltrating the 3D tumor and inducing apoptosis in cancer cells.

CONCLUSIONS
This study demonstrates the successful generation of a relevant human disease model using MPS systems. It can serve as an efficient drug screening platform and offers insights into the immune/tumor cells crosstalk

O-25 (YIA) Hybrid spheroids as a model of osteosarcoma

Martyna Malgorzata Rydzyk[1], Micaela Pannella[2], Concettina Cappadone[1], Giovanna Farruggia[1], Emil Malucelli[1], Cristina Pellegrino[1], Francesca Rossi[1], Stefano Iotti[1], Toni Ibrahim[2] and Enrico Lucarelli[2]
[1] Pharmaceutical Biochemistry Lab, Department of Pharmacy and Biotechnology, Alma Mater Studiorum University of Bologna, Via San Donato 19/2 40127 Bologna, Italy.
[2] Osteoncology, Bone and Soft Tissue Sarcomas and Innovative Therapies Unit, IRCCS Istituto Ortopedico Rizzoli, Bologna, Italy

OBJECTIVE
Osteosarcoma (OS) is the most common primary malignant bone tumour. The 5-year survival rate of patients treated with current therapy is 70% when the disease is localized and less than 20% when metastatic. Therefore, new drugs, such as tyrosine inhibitors, must be tested to improve patient's outcome. So far, most of the in-vitro pre-clinical studies have been conducted on a monolayer of OS cells. These models are limited because do not consider the tumour microenvironment (TME) in terms of cell composition and 3D structure.
The aim of this work is to develop 3D OS models including different tumor-associated cells (TAC) such as mesenchymal stromal cells, fibroblasts and endothelial cells co-cultured with cancer cells. Mimicking OS TME, we will obtain robust results representing the in vivo tumor behaviour.

MATERIALS AND METHODS
We built spheroids containing OS cells (MG63 and Saos-2) alone or co-cultured with TAC. Spheroids were made by self-assembly in 96-well ultra-low attachment. Spheroids growth was monitored for 7 days by PrestoBlue assay. Spheroids were fixed and embedded in OCT for further histological characterisation.

RESULTS
Results showed that MG63 monocultures developed a compact spheroid with a regular shape, while Saos-2 cells were unable to form a compact spheroid. When mesenchymal stromal cells, fibroblast and endothelial cells were added to cancer cells, the spheroids became more compact and viable, as stromal cells can furnish a physiological scaffold to tumor tissue. Confirming the validity of the model, the structure of the spheroids remains compact and viable over time.

CONCLUSIONS
TAC contributes to OS expansion and exert an impact on invasiveness, thus supporting the idea that these cells promote a tumorigenic effect. The developed spheroids reproduce better the complexity of TME in OS tumors, can enable a more accurate model for high-throughput screening anticancer drugs. Further studies will be conducted using patient's derived primary cells.

O-26 (YIA) Perinatal Stem Cell Spheroids in Type 1 Diabetes Therapy: Structural Insights and Immunomodulatory capacity

Francesca Paris[1], Valeria Pizzuti[1], Pasquale Marrazzo[1], Aurora Longhin[1], Laura Bonsi[1] and Francesco Alviano[2]
[1] Department of Medical and Surgical Sciences, University of Bologna, Italy

[2] Department of Biomedical and Neuromotor Science, University of Bologna, Italy

OBJECTIVE
Type 1 diabetes mellitus (T1DM) is a complex metabolic disorder characterized by the immune-mediated depletion of insulin-producing cells. Currently, the only effective treatment involves the daily administration of exogenous insulin. To address the underlying immunological trigger of T1DM remains a challenge, leading to a significant focus on advancing stem cell therapy. The growing interest in employing three-dimensional (3D) cell cultures to more accurately emulate in vivo conditions can be a strategy to enhance the immunomodulation and differentiation potential of cell-based treatments. The aim of this study is to establish a reliable 3D stem cell-based model that can be investigated to evaluate its immunomodulatory capacity, a potential cell therapy application in T1DM.

MATERIALS AND METHODS
To pursue this aim, we created a co-culture spheroid made of amniotic epithelial cells (AECs) and Wharton's jelly mesenchymal stromal cells (WJ-MSCs), assembling cells in a 1:1 ratio. The resulting spheroid was analyzed for viability, extracellular matrix production, and hypoxic state. Furthermore, we evaluated the immunomodulatory ability of spheroids by co-culturing them with activated PBMCs or T cells. After 72 hours of co-culture PBMCs or T-cells were recovered and analyzed with flow cytometry.

RESULTS
Our findings suggest that co-culture spheroids are stable during long-term culture while maintaining viability, producing a consistent extracellular matrix, as observed by immunofluorescence staining. The cellular model modulated activated PBMCs and T-cells by reducing their proliferation and activation, without affecting the viability of immune cells. Additionally, co-culture spheroids promote a tolerogenic response by enhancing regulatory T-cell proliferation compared to control PBMCs.

CONCLUSIONS
The obtained co-culture model holds the potential as an encouraging cellular therapy solution for T1DM and regenerative medicine. The assessment of the whole immunomodulatory and differentiative capacities of such a model requires further elucidation, necessitating an increase in the evaluation of the immune cell population involved in T1DM research.

O-27- iPSC as model systems for neurodevelopmental and neurodegenerative diseases.

Claudia Compagnucci[1]
[1] Molecular Genetics and Functional Genomics, Bambino Gesù Children's Hospital, IRCCS, Rome, Italy. Electronic address: claudia.compagnucci@opbg.net.

Many neurodegenerative and neurodevelopmental diseases can be studied in vivo using different animal models (i.e., mouse, rat, drosophila, zebrafish), but the human physiology is unique and sometimes the animal model does not recapitulate the human phenotype, as is the case for Riboflavin transporter deficiency (RTD). To understand the biological mechanisms altered in RTD, we decided to use induced pluripotent stem cells (iPSCs) as an in vitro human disease model of this and other neurological disorders. iPSCs are pluripotent stem cells derived from adult somatic cells (i.e., skin fibroblasts) which are genetically reprogrammed to an embryonic stem (ES) cell-like state through forced expression of genes important for maintaining the pluripotency. iPSCs can be successfully differentiated into somatic cells, i.e. neurons and astrocytes. Thus, this in vitro model permits to recapitulate and reproduce the onset pathology in a patient-specific way and it can be highly informative in understanding the patho-mechanisms of human neurodevelopmental and neurodegenerative disorders and it can be used as a proof of concept system for efficacious treatments on human patients

O-28 Differentiation of human induced pluripotent stem cells into hyalocytes through ascorbic acid treatment

Elena Laura Mazzoldi[1], Rosalba Monica Ferraro[1], Gabriele Benini[1], Piergiuseppe Sacristani[2], Daniele Lussignoli[2], Francesco Semeraro[2], Silvia Clara Giliani[1]
[1] Angelo Nocivelli Institute for Molecular Medicine, Department of Molecular and Translational Medicine, University of Brescia, Viale Europa 11, 25123 Brescia, Italy
[2] Department of Ophthalmology, University of Brescia, Spedali Civili, Piazzale Spedali Civili 1, 25123 Brescia, Italy

OBJECTIVE
Hyalocytes are a tiny population of macrophage-like cells which reside in the vitreous cortex of the eye. Even though hyalocytes have been known for 150 years, they have been poorly investigated. Recently, their involvement in both physiological processes and pathological conditions of the vitreoretinal interface has been suggested. However, essentially hyalocytes from slaughtered animals have been cultured, while studies on cultured human hyalocytes are few, since their isolation requires an invasive surgery. To this reason, the aim of this study is to differentiate hyalocytes from human induced pluripotent stem cells (iPSCs) to have an unlimited source of cells in a non-invasive way.

MATERIALS AND METHODS
iPSCs, routinely cultured on Matrigel-coated multiwell plates, were firstly differentiated into hematopoietic stem cells (HSCs) through a commercial kit, and then into macrophages by macrophage colony-stimulating factor (M-CSF) treatment. Macrophages were then left untreated (NT) or treated with ascorbic acid alone or in combination with basic fibroblast growth factor (bFGF) and/or transforming growth factor beta 1 (TGFβ1). As positive control, macrophages have also been cultured in the presence of a pool of vitreous bodies from vitrectomies. Cells have been analyzed from a morphological point of view, and for gene and protein expression by qRT-PCR, western blot, immunofluorescence, and flow cytometry at day 7, 14, and 21 of culture.

RESULTS
Similar to vitreous-treated cells, macrophages treated with ascorbic acid alone or in combination with bFGF presented a more elongated shape as compared to NT or cells treated with TGFβ1. Moreover, the same treatments induced S100A4, S100A10, S100B, and CX3CR1 gene expression downregulation, while upregulated COL6A1 and HLA-DRA. At protein level, S100B, CD14, and CD49d resulted downregulated with all the treatments, especially at longer timepoints.

CONCLUSIONS
Hyalocytes can be differentiated by treating iPSC-derived macrophages with ascorbic acid alone for 21 days.

O-29 MULTIPLE SYSTEM ATROPHY IPSC-DERIVED DOPAMINERGIC NEURONS DISPLAY STRUCTURAL AND FUNCTIONAL DIFFERENCES FROM CONTROL.

Giulia Sofia Marcotto[1], Laura Cavalleri[1], Jonida Bitraj[1], Giulia Pe[1], Maria Teresa Tedesco[2], Alessandro Maccione[2], Chiara Rosa Battaglia[2], Giovanna Piovani[1], Maria Teresa Pellecchia[3], Paolo Barone[3], Verdon Taylor[4], Emilio Merlo Pich[5], Ginetta Collo[1]
[1], University of Brescia, Department of Molecular and Translational Medicine, Brescia, Italy.
[2], 3Brain AG, Wädenswil, Switzerland.
[3], University of Salerno, Department of Medicine, Surgery and Dentistry "Scuola Medica Salernitana", Salerno, Italy
[4], University of Basel, Department of Biomedicine, Basel, Switzerland.
[5], Gelf Health, Milano, Italy

Objective: Multiple system atrophy with predominant parkinsonism (MSA-P) is a sporadic, adult-onset, fatal neurodegenerative disease with a severe clinical course differentially affecting several neuronal pathways including the mesencephalic district of the nigrostriatal system.
In this work, we applied the induced pluripotent stem cells (iPSCs) technology to generate a model of MSA in order to characterize its morphological and functional features.
Materials and Methods: iPSC lines were generated by reprogramming peripheral blood mononuclear cells from MSA-P and healthy donors who signed the Informed Consent. Selected clones were studied for their pluripotency, trilineage differentiation potential, and karyotype. iPSCs were subsequently differentiated in floorplate (FP)-derived midbrain dopaminergic (DA) neurons using a well-established protocol based on the combined temporal administration of small molecules and growth factors. At day 30, tyrosine hydroxylase (TH)-positive neurons were visualized by immunofluorescence, confirming the presence of the DA phenotype. For morphological studies DA neurons were fixed at day 40 of differentiation and immunostained with an anti-TH antibody and evaluated by computer-assisted morphometry examining three morphological aspects: (i) the maximal length of dendrites, (ii) the number of primary dendrites and (iii) the soma area. Functional features were investigated using microelectrode array systems (MEAs) (3Brain AG) at days 35 and 45 of differentiation.
Results: A comparative morphological study of patient and controls DA neurons showed a significant reduction of dendritic length and soma area in patient DA neurons compared with four different controls. MEAs recordings showed a higher percentage of spikes in a burst in the patient's neuronal cultures than in the control indicating less firing stability and the predisposition to increase the number of spikes within a burst, which is typical of immature or dysfunctional cell membranes.
Conclusions: These preliminary data reveal a different morphological and functional phenotype of neurons in MSA-P iPSC-derived mesencephalic cultures.

POSTER COMMICATIONS

In alphabetical order

Alessia Ambrosino - Irisin plays a role in shaping the development of mesenchymal stromal cells, encouraging a predisposition toward promoting beige fat formation.

Alessia Ambrosino[1], Afshin Samiminemati[1], Girolamo Di Maio[1], Giovanni Di Bernardo[1]
[1] Department of Experimental Medicine, Biotechnology and Molecular Biology Section, University of Campania "Luigi Vanvitelli", Naples, Italy

OBJECTIVE

In light of the growing global obesity crisis, the importance of pharmaceutical interventions has become paramount. Recognizing the worldwide severity of obesity highlights the critical significance of physical activity in its effective prevention and management. It is well-known that muscle exercises stimulate the production of several molecules called myokines, which play a crucial role in preserving metabolic homeostasis. Among these myokines is Irisin, a 12 kDa protein secreted by skeletal muscles in both mice and humans. Mesenchymal Stromal Cells (MSCs) are found within white adipose tissue (WAT) and possess the capacity to differentiate into a range of cell types, including adipocytes, osteocytes, and chondrocytes. In our study, we aim to assess Irisin's influence on the stemness properties of MSCs.

MATERIALS AND METHODS

We treated the MSCs with Irisin for 7 days. Subsequently, we evaluated the biological and stemness properties of the MSCs using several assays.

RESULTS

Irisin treatment did not modify the percentage of cycling cells, and their distribution in the G1, S, G2/M populations. Following Irisin treatment, we detected an increase of P16/CDKN2A and BCL2, a decrease of P21/CDKN1A and no modification of RB2/P130 and P27/CDKN1B. This suggests no apoptosis or senescence onset, confirmed by Annexin V and beta-galactosidase assays. Any modifications in the phenotype of MSCs may also impact the stemness potential of their stem cell subpopulation. We noted a significant reduction in the expression of key transcription factors responsible for maintaining stem cell lineage potency and self-renewal. These findings were substantiated by immunocytochemistry and colony forming unit (CFU) assays. Irisin treatment led to a decrease in the expression of genes associated with osteocyte and chondrocyte differentiation. Conversely, the differentiation of adipocytes displayed a distinct pattern of gene expression. Specifically, there was an upregulation in the expression of C/EBPα and C/EBPβN genes, accompanied by a downregulation of PPARγ. Notably, the differentiation of white adipocytes exhibited a shift toward a "browning" phenotype, as evidenced by an increased number of cells expressing UCP1.

CONCLUSIONS

Our study suggests Irisin as a promising obesity therapy, but caution is needed due to stem cell and differentiation concerns. Further animal research will help assess Irisin's safety and effectiveness for obesity treatment.

(YIA) Secretome isolated from Mesenchymal Stromal Cells loaded with Paclitaxel have cytotoxic effect on osteosarcoma cell lines.

Alessia Giovanna Santa Banche Niclot[1], Elena Marini[1], Ivana Ferrero[2], Camilla Francesca Proto[1], Francesco Barbero[3], Ivana Fenoglio[3], Alessandro Barge[4], Valentina Coccè[5], Francesca Paino[5], Katia Mareschi[1,2] and Franca Fagioli [1,2]

1 Department of Public Health and Paediatrics, The University of Turin, Piazza Polonia 94, 10126 Torino, Italy;

2 Stem Cell Transplantation and Cellular Therapy Laboratory, Paediatric Onco-Haematology Division, Regina Margherita Children's Hospital, City of Health and Science of Turin, 10126 Torino, Italy;

3 Department of Chemistry, University of Turin, Via Pietro Giuria 7, 10125 Turin, Italy;

4 Department of Drug Science and Technology, University of Turin, Via Pietro Giuria 9, 10125 Turin, Italy;

5 CRC StaMeTec, Department of Biomedical, Surgical and Dental Sciences, University of Milan, 20122 Milan, Italy.

OBJECTIVE

Osteosarcoma (OS) is a rare tumor that constitutes a very aggressive disease for children and adolescents and still represents an important challenge for the clinicians. Our aim was to develop a new therapeutic approach based on secretome (SECR) isolated from Bone Marrow (BM) Mesenchymal Stromal Cells (MSCs) loaded with Paclitaxel (PTX) to produce Extra Vesicles (EVs) as drug carriers for OS treatment. We performed pre-clinical studies to test the cytotoxic effect of PTX-MSC-SECR on 3 OS cell lines (SJSA, MG63 and HOS).

MATERIALS AND METHODS

We produced 3 batches of SECR from 3 BM-MSC samples treated for 24 h with 15 µg/ml of PTX or with standard medium. The SECR batches (pure and diluted 1:2 and 1:4) were tested for their cytotoxic effect after 5 days of exposure on the 3 OS cell lines by MTT Assay. The same samples were analyzed for EVs size distribution, particles concentration and Zeta potential by Nanoparticle Tracking Analysis (NTA) and for the PTX presence by ultra-high performance liquid chromatography-tandem mass spectrometry (UPLC-MS/MS).

RESULTS

All OS cell lines showed a statistically significant viability decrease after treatment with each SECR-PTX batches in a dose-response manner and the cell viability did not decrease after treatment with SECR isolated from MSCs without treatment with PTX. The NTA analyses showed the presence of nanoparticles with a mean size comparable to EVs. The HPLC analyses detected the presence of PTX in minimal doses in all PTX-SECR batches.

CONCLUSION

We demonstrated that the secretome isolated from the PTX-loaded MSCs had a strong cytotoxic effect on OS cell lines which allows us to propose it as an ideal candidate for drug delivery as an innovative treatment of pediatric osteosarcoma.

(YIA) 3D PRINTED SILK FIBROIN BIOINK FOR CONTROLLED RELEASE OF MESENCHYMAL STEM CELL EXTRACELLULAR VESICLES IN TENDON REGENERATION

Elia Bari[1], Giulia Maria Di Gravina[2], Franca Scocozza[3], Sara Perteghella[4,5], Benedetta Frongia[3], Sara Tengattini[4], Lorena Segale[1], Lorella Giovannelli[1], Maria Luisa Torre[1,5] and Michele Conti
[1] University of Piemonte Orientale, Department of Pharmaceutical Sciences
[2] University of Pavia, Department of Electrical, Computer and Biomedical Engineering
[3] University of Pavia, Department of Civil Engineering and Architecture
[4] University of Pavia, Department of Drug Sciences
[5] PharmaExceed S.r.l.

OBJECTIVE

This study aims to obtain a bioink made of sodium alginate (SA) and silk fibroin (SF) and enriched with lyosecretome – a freeze-dried formulation of mesenchymal stem cell-secretome containing extracellular vesicles (EVs) – for 3D bioprinting applications in tendon regeneration.

MATERIALS AND METHODS

The preparation process of SF was optimized by adjusting the degumming time (needed for its extraction from the *Bombyx mori* cocoon) to achieve a printable protein solution. Blends of SA and SF degummed at different times (i.e., 1, 2, and 4 h) were characterized in terms of printability and shape fidelity. The tensile and compressive mechanical tests were conducted to reveal how the SF component could influence the mechanical performance of the material. Then, the SA-SF hydrogels were loaded with lyosecretome obtained from AD-MSCs to investigate the release of EVs over time. Finally, porous parallelepiped-shaped scaffolds (10×10×3 mm) were prepared by co-printing poly-caprolactone with SA-SF hydrogel containing lyosecretome (2×10^6 cell equivalents), and their colonization by tenocytes was investigated over time (5, 14 and 21 days) by MTT. Scaffolds without SF and/or lyosecretome were used as a control.

RESULTS

SF degummed for 1, 2, and 4 h was printable without any clotting of the needle. A good shape fidelity in the pores was observed, while the material's viscous and potentially collapsible nature was highlighted. SF degummed for 1, 2, and 4 h was even still able to change its conformation from Silk I (a mix of a random coil, α-helix domains, and β-turn structures) to Silk II (abundant β-sheet structures) when treated with 20% w/v KCl, thus achieving good mechanical properties. Indeed, the mechanical characterization of SA-SF hydrogels revealed that their compressive moduli were three times higher than that of the hydrogel formed with only SA. Conversely, no influence on tensile response was detected. The addition of SF significantly modified the release of EVs with respect to the baseline of SA-only hydrogel, which was considered a control, depending on the degumming time: the release of EVs was slowed down when 2 and 4 h degummed SF were used. Finally, tenocyte proliferation increased over time and was significantly higher when SF and lyosecretome were combined.

CONCLUSIONS

These results lay the foundation for developing SA-SF bioinks with modulable mechanical and EV-release properties and their application in 3D scaffold printing for tendon regeneration.

(YIA) HEPATIC BIOMIMETIC COMPONENT FOR 3D-PRINTABLE HYBRID HYDROGELS: A NEW PRODUCT FOR LIVER REGENERATION

Elia Bari[1§], Giulia Maria Di Gravina[2,3§], Stefania Croce[4,5], Franca Scocozza[3], Lorenzo Cobianchi[4,5], Ferdinando Auricchio[3], Maria Antonietta Avanzini[5], Maria Luisa Torre[1,6] and Michele Conti[3]
[1] University of Piemonte Orientale, Department of Pharmaceutical Sciences
[2] University of Pavia, Department of Electrical, Computer and Biomedical Engineering
[3] University of Pavia, Department of Civil Engineering and Architecture
[4] University of Pavia, Department of Clinical, Surgical, Diagnostic & Pediatric Sciences
[5] IRCCS Policlinico S. Matteo
[6] PharmaExceed S.r.l.
§ equally contributed

OBJECTIVE
This study aims to obtain lyodECM – a hepatic lyophilized decellularized extracellular matrix – in the form of an "off-the-shelf" and free soluble powder that will be integrated as a biomimetic component into bioinks for reliable hepatic 3D bio-printed models.

MATERIALS AND METHODS
The decellularized hepatic tissue was suspended in 0.1 M HCl with a final concentration equal to 2% w/v and digested with pepsin for 24 h using a magnetic stirrer set at 37 °C; two different amounts of pepsin were assessed (10% and 20% w/w) with respect to the tissue weight to be digested. The achieved solution was subsequently lyophilized, obtaining lyodECM, which was tested for cytocompatibility by MTT on human (h) and porcine (p) mesenchymal stem cells (MSCs) and immunogenicity on human peripheral blood mononuclear cells (hPBMC). The suitability of lyodECM to create a microenvironment in which enzymes, i.e., elastase, can function at their maximum activity, with a certain tolerance to inhibitory mechanisms, was also assessed. Finally, the lyodECM powder (3.75 mg/ml) was incorporated in a blend of alginate (8% w/v) and gelatine (4% w/v); the window of printability and construct mechanical stability were assessed.

RESULTS
Both the concentrations of pepsin tested successfully digested the tissue into a solution; however, the 20% w/w concentration was selected due to its faster digestion rate. After lyophilization, lyodECM was obtained as a white, water-soluble powder. In the experiments with pMSCs, a dose-dependent decrease in cell metabolic activity after 3 and 7 days of treatment with lyodECM was observed. Up to a concentration of 3.75 mg/ml, cell metabolic activity remained above 70% after 7 days. For hMSCs, we observed an increase in cell metabolic activity up to 1.9 mg/ml after 3 days, followed by a decrease, with cytotoxic effects observed at 15 mg/ml; after 7 days, lyodECM was cytotoxic only at 15 mg/mL. LyodECM demonstrated non-immunogenic properties, as it did not stimulate the proliferation of hPBMCs, and at concentrations above 2.5 mg/ml, it created a microenvironment where elastase can function at its maximum activity, with tolerance to inhibitory mechanisms. Lastly, lyodECM was incorporated in the ALG/GEL hydrogel at the concentration of 3.75 mg/ml and successfully used for 3D printing.

CONCLUSIONS
These results lay the foundation for developing reliable hepatic 3D bio-printed models.

(YIA) ENHANCING DRUG DELIVERY BY A CARRIER-IN-CARRIER PLATFORM: SILK FIBROIN NANOPARTICLES EMBEDDED IN MESENCHYMAL STEM CELL EXTRACELLULAR VESICLES

Elia Bari[1], Lorella Giovannelli[1], Lorena Segale[1], Dmitry Lim[1], Maria Luisa Torre[1,2]
[1] University of Piemonte Orientale, Department of Pharmaceutical Sciences
[2] PharmaExceed S.r.l.

OBJECTIVE

This study aims to obtain a “smart” and advanced hybrid drug delivery system featuring a “carrier-in-carrier” structure: silk-fibroin nanoparticles (SFNs) in the core, capable of loading high quantities of hydro- or lipophilic drugs and releasing them in a controlled manner, encased within a lipid shell composed of mesenchymal stem cell extracellular vesicles (EVs), which offer effective uptake and targeting capabilities.

MATERIALS AND METHODS

SFNs were prepared by desolvating a silk fibroin solution in acetone (where curcumin, selected as a model drug, was previously solubilized). EVs were isolated from culture supernatants by tangential flow filtration. To prepare the carrier-in-carrier, SFNs and EVs were co-incubated in a 1:1 and 1:2 ratio and then sonicated using an ultrasonic probe with 20% amplitude for six cycles; each cycle is 3 min, covering six periods of 30 s on/off with a 2 min cooling period between each cycle. The effective formation of a carrier-in-carrier structure was assessed by confocal microscopy. The stability of the carrier-in-carrier as a function of time was evaluated by measuring the size of dispersions stored at 4 °C up to 25 days. Finally, the stability of the drug (curcumin) when free or encapsulated in the carrier-in-carrier was evaluated for up to 8 days at 25 °C.

RESULTS

The effective formation of the carrier-in-carrier was verified by confocal microscopy analysis: for both tested SFN/EV ratios (1:1 and 1:2), there was overlapping fluorescence of fibroin (auto-fluorescence) and EVs (marked with Nile Red). To better understand the arrangement of SFNs and EVs relative to each other, the variation in the Z-plane was analyzed: assuming that the carrier-in-carrier has a spherical shape, the red fluorescence (EVs) predominated at the base and the apex of the sphere, while in the center, green fluorescence (SFNs) was observed, surrounded by red fluorescence (EVs). This indicates that EVs have enveloped SFNs, as desired. The carrier-in-carrier showed a mean diameter of about 150 nm and did not aggregate for up to 25 days when stored at 4 °C; conversely, SFNs alone and EVs alone showed an increase in their mean diameter due to aggregation (or fusion, for EVs). After 8 days at 25 °C, the residual absorbance % of the drug was 20% for free curcumin and above 60% when the drug was encapsulated in the carrier-in-carrier, confirming the ability of the developed system to protect drugs.

CONCLUSIONS

The technology proposed allows for an innovative and “smart” drug delivery system able to protect the drug encapsulated. Further tests are needed to verify the specific targeting and selective uptake by target cells provided by the EV component of the carrier-in-carrier.

(YIA) A TRIDIMENSIONAL PRECLINICAL MODEL OF HUMAN AND CANINE OSTEOSARCOMA TO STUDY THE EVOLUTION OF CANCER STEM CELLS

Elia Bari[1§], Ania Naila Guerrieri[2§], Gabriele Scattini[3§], Andrea Cacciamali[4], Martina Angela Checco[4], Aurora De Mattia[4], Toni Ibrahim[2], Maria Luisa Torre[1], Luisa Pascucci[2], Enrico Lucarelli[2] and Silvia Dotti[4]
[1] University of Piemonte Orientale, Department of Pharmaceutical Sciences
[2] IRCCS Istituto Ortopedico Rizzoli, Osteoncology, Bone and Soft Tissue Sarcomas and Innovative Therapies
[3] University of Perugia, Department of Veterinary Medicine
[4]Istituto Zooprofilattico Sperimentale della Lombardia e dell'Emilia-Romagna, Centro di Referenza Nazionale per i Metodi Alternativi, Benessere e Cura degli Animali da Laboratorio
[§] Equal contribution

OBJECTIVE
Osteosarcoma (OS), an aggressive sarcoma, can lead to disease progression and lung metastasis; efforts are focused on mitigating recurrence and metastasis risk by targeting non-responsive OS cancer stem cells, driving the need for models that replicate the tumor microenvironment. This study aims to develop a 3D system under anchorage-independent conditions to foster the isolation of cancer stem cells and replicate human and canine OS microenvironment for studying OS development and evaluating anticancer agents in preclinical *settings*.

MATERIALS AND METHODS
The selected cell lines were 143B-GL (aggressive and metastatic) and MG63-GL (fibroblastic and non-metastatic) for the human model and D17 cells for the canine one. Cells were suspended in 0.5% w/v xanthan gum in 100 mM Calcium chloride and then gently dripped using a syringe (without a needle) into a 0.5% w/v alginate solution. Capsules were also prepared by dispersing a demineralized bone matrix (DBM, 3% w/v) in the xanthan gum to provide a more bone tissue-like microenvironment. After filtration, capsules were characterized in size and cultured in static conditions for up to 21 days. Cell viability, gene and protein expression were assessed after 1, 7, 14, and 21 days; histochemical (E&E, Alizarin red, Alcian blue staining), immunohistochemical (SATB2, Ki67), and confocal microscopy analyses were performed.

RESULTS
Alginate capsules, with a mean diameter of 6.41 ± 0.254 mm (n=20), sustained growth for 14 and 21 days for human and canine OS cell lines, respectively. DBM boosted 143B-GL cell proliferation and increased MG63-GL cell migration from capsules, supported by an increase in MMP9 expression, similar to 143B-GL cells. Histochemistry revealed DBM remodeling, with a reduction in fragment size, collagen deposition, calcium accumulation, and potential mineralization. On day 21, immunohistochemistry for SATB2, an osteogenic marker, showed strong positivity in cell aggregates formed by the more aggressive 143B-GL cells and lower in the less aggressive MG63-GL cells.

CONCLUSIONS
Our 3D model effectively replicates human and canine OS environments in vitro. Given the precedent of similar techniques in isolating and enriching cancer stem cells under anchorage-independent growth conditions, our model holds promise, after further optimization, for investigating aggressive OS cell populations, which play a pivotal role in disease recurrence and poor outcomes.

(YIA) Human dental pulp stem cells promote the lipofibroblast transition in the early stage of a fibro-inflammatory process

Giulia Bertani[1], Alessandra Pisciotta[1], Rosanna Di Tinco[1], Giulia Orlandi[1], Laura Bertoni[1], Elisa Pignatti[1], Monia Orciani[2], Paola Sena[1], Jessika Bertacchini[1], Carlo Salvarani[1,3†] and Gianluca Carnevale[1†]
[1] Department of Surgery, Medicine, Dentistry and Morphological Sciences with Interest in Transplant, Oncology and Regenerative Medicine, University of Modena and Reggio Emilia, Modena, Italy
[2] Department of Clinical and Molecular Sciences, Polytechnic University of Marche, Ancona, Italy
[3] Unit of Rheumatology, Azienda Unità Sanitaria Locale-IRCCS, Reggio Emilia, Italy

OBJECTIVE
There is a close correlation between chronic inflammation and fibrosis in autoimmune diseases, particularly in systemic sclerosis and chronic periaortitis. A better comprehension of the molecular mechanisms exerted by cell types implicated in fibro-inflammation is needed to develop novel therapeutic strategies, since the currently used drugs prove mostly effective in suppressing inflammation. Mesenchymal stromal/stem cells (MSCs) are being matter of deep investigation to unveil their role in the evolution of fibrogenetic process. Several findings pointed out the controversial implication of MSCs in these events, with reports lining at a beneficial effect exerted by external MSCs and others highlighting a direct contribution of resident MSCs in fibrosis progression. Human dental pulp stem cells (hDPSCs) were demonstrated to hold promise as potential therapeutic tools due to their immunomodulatory properties, which strongly support their contribution to tissue regeneration. Based on these considerations, the aim of the present study was to evaluate the role of hDPSCs in an in vitro model of fibro-inflammation mimicked by a co-culture system with fibroblasts exposed to TGF-β1.

MATERIALS AND METHODS
This study evaluated hDPSCs response to a fibro-inflammatory microenvironment, mimicked in vitro by a transwell co-culture system with human dermal fibroblasts, at early and late culture passages, in presence of TGF-β1, a master promoter of fibrogenesis.

RESULTS
The results obtained indicate that hDPSCs promote a myofibroblast-to-lipofibroblast transition, likely based on BMP2 dependent pathways, when exposed to acute fibro-inflammatory stimuli. In contrast hDPSCs reduce their anti-fibrotic effect and acquire a pro-fibrotic phenotype, when a chronic fibro-inflammatory microenvironment is generated.

CONCLUSIONS
We argued that BMP2 upregulation in hDPSCs might have exerted either an autocrine effect on stem cells themselves, avoiding their activation toward myofibroblasts, or paracrine effects on TGF-β1-pre-stimulated hDFs, leading their transition toward the lipofibroblast phenotype. At the same time, when a pro-fibrotic compartment is already activated and further induced toward the deposition of ECM components, hDPSCs are no longer capable of exerting a modulatory effect. These data provide the basis for further investigations on the response of hDPSCs to varying fibro-inflammatory conditions.

(YIA) Establishment of reliable in vitro models to study the mechanism of action of Mesenchymal stromal cell-derived extracellular vesicles toward the inhibition of fibrosis and oxidative stress.

Paola Bisaccia[1,2], Agner Henrique Dorigo Houchuli[1,2], Alice Zaramella[1,2], Miriam Duci[1,2], Marcin Jurga[3], Maurizio Muraca[2,4,5], Michela Pozzobon[1,2].

[1] Stem Cells and Regenerative Lab, Institute of Pediatric Research Città della Speranza, Padova, Italy.

[2] Department of Women and Children Health, Università di Padova, Italy

[3] Exobiologics (NV), Belgium

[4] Institute of Pediatric Research Città della Speranza, Padova, Italy.

[5] Lifelab Program, Consorzio per la Ricerca Sanitaria (CORIS), Veneto Region, Padova, Italy

OBJECTIVE

The regenerative and anti-inflammatory capabilities of mesenchymal stromal cells-derived extracellular vesicles (MSC-EVs) are well known. In our previous work we found that MSC-EVs can prevent the main outcomes of Bronchopulmonary Dysplasia (BPD) in rat models, such as fibrosis and oxidative stress. To reveal the mechanisms of action of MSC-EVs toward these two biological processes, we developed two reliable *in vitro* models.

MATERIALS AND METHODS

The fibrosis assay considered the use of primary macrophages isolated from rats' bone marrow. Macrophages were analysed for αSMA and CD90 expression by flow cytometry after TGFβ1 treatment for a time course of 7days and was tested the antifibrotic capability of MSC-EVs versus a positive control that is represented by an inhibitor of tgfβ receptor I. Cells were analysed by qRT PCR, immunofluorescence, and flow cytometry for profibrotic markers. As regard the oxidative stress assay, human alveolar epithelial cells from healthy donor were used. The cells were damaged with hydrogen peroxide and rotenone. The efficacy of MSC-EVs in scavenging oxidative damage versus vitamin C in a time course of 10days was investigated. The DNA oxidation and the cells proliferation by immunofluorescence were analyzed.

RESULTS

MSC-EVs suppressed the induction of αSMA in macrophages and the collagen deposition in a dose-response dependent manner, more than the inhibitor of tgfβ receptor I. In addition, MSC-EVs could protect the alveolar epithelial cells from the DNA oxidation in a dose response manner. MSC-EVs were a better scavenger than vitamin C. MSC-EVs exploited their action inhibiting the proliferation of the cells and then repairing the DNA damage.

CONCLUSIONS

MSC-EVs are able to counteract the onset of fibrosis and the oxidative stress in two *in vitro* models that recapitulate the major consequences of BPD diseas

Mesenchymal stromal cell-derived extracellular vesicles restore lung function and protectbrain from oxidative injury in a rat model of bronchopulmonary dysplasia.

Paola Bisaccia[1,2], Fabio Magarotto[1,2], Agner Henrique Dorigo Houchuli[1,2], Arben Dedja[3], Alice Zaramella[1,2], Miriam Duci[1,2], Marcin Jurga[4], Eugenio Baraldi[2], Andrea Porzionato[5], Maurizio Muraca[2,5], Michela Pozzobon[1,2,5].

[1] Stem Cells and Regenerative Lab, Institute of Pediatric Research Città della Speranza, Padova, Italy.

[2] Department of Women and Children Health, Università di Padova, Italy

[3] Department of Cardiac, Thoracic and Vascular Sciences and Public Health, University of Padua, Italy

[4] Exobiologics (NV), Belgium

[5] Institute of Pediatric Research Città della Speranza, Padova, Italy.

[6] Department of Neuroscience-DNS, Università di Padova, Italy

[7] Lifelab Program, Consorzio per la Ricerca Sanitaria (CORIS), Veneto Region, Padova, Italy

OBJECTIVES

In an animal model of hyperoxia-induced BPD, we assessed the effects of mesenchymal stromal cell-derived extracellular vesicles (MSC EVs) on the development of oxidative stress and fibrosis both in the lungs and in the brain, the two mostly affected organs of this disease.

METHODS

Good Manufacturing Practice (GMP)-grade EVs were produced by human Wharton-Jelly and were characterized according to MISEV2018. Rat pups were divided in 3 groups: normoxia + PBS vehicle, hyperoxia with PBS, hyperoxia with MSC-EVs in PBS. Both PBS and MSC EVs were injected intratracheally (IT) on days 3, 7 and 10 and pups were sacrificed on day 14. The expression of the profibrotic genes, concentration of glycosaminoglycans and the balance between the two types of alveolar cells (ATI and ATII) were analyzed in the lungs as well as protein surfactant and collagen expression. Mitochondrial lamellar bodies were analyzed by TEM. Oxidative damage was assessed by protein carbonylation and by DNA oxidation in the lungs and in the brain.

RESULTS

Pups under hyperoxia exhibited increased collagen deposition and expression of pro-fibrotic genes in the lungs. Both parameters were reduced by treatment with MSC EVs. The lung epithelial compartment showed improvement in glycosaminoglycan and surfactant protein expression as well as in lamellar bodies shape in MSC EVs-injected rat pups compared to untreated animals. Both protein carbonylation and DNA oxidation were reduced by MSC EV treatment. Furthermore, the levels of glial fibrillary acidic protein (GFAP) in the brain were significatively reduced with the MSC EVs. The results of this work have been included in an Investigational Medicinal Product dossier that was approved by the European Medicines Agency as experimental treatment of BPD. The clinical trial will start in October 2023.

CONCLUSIONS

In a neonatal model of BPD, IT administration of MSC EVs counteracts the development of fibrosis, improve pulmonary epithelial function in the lungs and protects against oxidative stress both in the lungs and in the brain.

(YIA) How to train your cells: cytokine priming to address ASC conditioned medium against inflammation

Francesca Cadelano[1,2,3], Chiara Giannasi[1,2], Elena Della Morte[2], e Anna Teresa Brini[1,2]
[1] Department of Biomedical, Surgical and Dental Sciences, University of Milan, Milan, Italy
[2] IRCCS Istituto Ortopedico Galeazzi, Milan, Italy
[3] PhD student in Experimental Medicine, University of Milan, Milan, Italy

OBJECTIVES

The conditioned medium (CM) from Mesenchymal Stem/stromal Cells (MSCs) possesses promising features that can be exploited for regenerative medicine purposes. Here we characterized the composition of the CM obtained from adipose MSCs (ASCs), either naïve or primed with inflammatory cytokines, and compared their chemotactic properties and anti-catabolic effects.

MATERIALS AND METHODS

CM were produced from confluent ASCs after 72 hours in starving conditions. The priming step was performed by adding 10ng/ml IL-1β and/or TNFα to the cultures for 5 minutes and accurately rinsing cells before starvation. We concentrated the CM using 3kDa cut-off Amicon filtering units and analyzed the resulting products for protein content, particle concentration, and the levels of various immunoregulatory, anti-inflammatory, and pro-resolving factors using different techniques such as Nanoparticle Tracking Analysis (NTA), immunoassays, and mass spectrometry. We evaluated the effects of control and primed CM (CM and pCM) in vitro on THP-1 cells to assess cell attraction and on inflamed articular chondrocytes to evaluate the inhibition of matrix metalloproteinase (MMP) activity. We analyzed the data using one-way or two-way ANOVA and set significance at $p < 0.05$.

RESULTS

The characterization of CM and pCM highlighted an enrichment in the latter of total protein content and number of particles, together with higher levels of immunoregulatory, anti-inflammatory, and protective mediators (e.g. TGF-β1, PGE2, and CCL-2). Surprisingly, CM and pCM were equally effective in attracting THP-1 cells, although cytokine priming induces the accumulation of chemoattractants in pCM. At last, both products efficiently hampered the pathological activity of MMPs in TNFα-inflamed chondrocytes, even though the levels of TIMP-1 and -2 were reduced in pCM.

CONCLUSIONS

The comprehensive characterization of naïve and primed CM confirms the hypothesized empowering effect of the priming strategy, demonstrating that it could be a mighty tool to obtain a richer and pathology-tailored product. Both CM showed comparable abilities to attract cells and prevent catabolism, but their uncharted mechanisms and any real differences should be studied in more complex *in vitro* and *in vivo* models, which include other components of the immune system and consider inter-tissue crosstalk and repairing mechanisms

Extracellular vesicles released by human mesenchymal stromal cells cultured in chemically defined medium exert anti-fibrotic effect in *in vitro* models of liver fibrosis

Giulia Chiabotto[1], Elena Ceccotti[1], Marco Guenza[1], Giovanni Camussi[1,2], Stefania Bruno[1,2]
[1] Department of Medical Sciences, University of Torino, Torino, Italy
[2] Molecular Biotechnology Center, University of Torino, Torino, Italy

OBJECTIVES

Hepatic fibrosis develops from chronic liver injury and is characterized by hepatocyte damage and activation of hepatic stellate cells (HSCs), which are primarily responsible for enhanced extracellular matrix deposition. Hence, hepatocyte regeneration and reversal of the activated phenotype of HSCs represent possible strategies to counteract the development of liver fibrosis. Extracellular vesicles (EVs) are nanosized membrane structures that can transport proteins, lipids and nucleic acids to target cells. Previous studies indicate that EVs derived from mesenchymal stromal cells (MSCs) have pro-regenerative properties and inhibit the activation of HSCs. However, potential contamination by culture medium components should be avoided for translational applications of EVs. Here, we investigated the effect of EVs derived from MSCs grown in chemically-defined media on activated HSCs and liver spheroids.

MATERIALS AND METHODS

Human MSCs isolated from bone marrow and umbilical cord were provided by RoosterBio® and expanded in RoosterNourish™-MSC-XF medium. To obtain EVs, supernatant from MSCs cultured in chemically-defined RoosterCollect™-EV medium was deprived of cell debris and apoptotic bodies by centrifugation and filtration, and then centrifuged at 100,000 g for 2 hours at 4°C. EV concentration, size and integrity were evaluated using NanoSight NS300 system and transmission electron microscopy. EV phenotype analysis was performed using flow cytometry. Different doses and regimens of EVs were used to stimulate human hepatic stellate cells (LX-2) activated by transforming growth factor-beta 1 (TGF-β1) and liver spheroids generated using Sphericalplate 5D™, in which LX-2 were co-cultured with human hepatoma cells (HepG2) in the presence of TGF-β1. The expression of the fibrosis-related alpha-smooth muscle actin (α-SMA), collagen I and TGF-β1 genes was monitored on RNA isolated from LX-2 and liver spheroids, by real time PCR analysis.

RESULTS

Increased expression of pro-fibrotic genes was detected in LX-2 and in liver spheroids following TGF-β1 stimulation. Treatment with MSC-EVs reduced the expression of collagen I and α-SMA in activated LX-2 and the expression of collagen I and TGF-β1 in liver spheroids.

CONCLUSIONS

We demonstrated that EVs derived from MSCs maintained in a chemically-defined medium can attenuate the activated phenotype of HSCs and liver spheroids, using two *in vitro* models of liver fibrosis developed in our laboratory.

Mesenchymal Stem Cells and Erythroid Progenitors: a co-culture for modeling the niche of Parvovirus B19 infection

Chiara Gamberini[1], Pasquale Marrazzo[2], Gloria Bua[3], Giorgio Gallinella[3], Laura Bonsi[2], Francesco Alviano[1]
[1]Department of Biomedical and Neuromotor Sciences, University of Bologna, 40126 Bologna, Italy
[2]Department of Medical and Surgical Sciences, University of Bologna, Bologna, Italy;
[3]Department of Pharmacy and Biotechnology, University of Bologna, Bologna, Italy;

OBJECTIVE
Parvovirus B19 (B19V) is a human pathogenic virus able to cause anemia in immunodeficient people by targeting bone marrow Erythroid Progenitor Cells (EPCs). Moreover, the characteristics of B19V include the ability to cross the placenta, infecting the fetus, and persist in other several tissues. The bone marrow hematopoietic niche, including the compartment of mesenchymal stromal cells (MSCs), is an important component in defining the tropism of this virus. Our previous findings showed that MSCs constitute a non-productive environment without significant viral replication; however, MSCs can operate as a reservoir of B19V DNA. Then, we aim to investigate the dynamics of B19V infection in co-cultures of MSCs with EPCs, which are generated *in vitro* starting from PBMCs.

MATERIALS AND METHODS
First, we optimized the co-culture conditions, in order to create the best supportive environment for both types of cells. Secondly, we characterized the progress of the infection in the MSCs-EPCs co-culture, comparing it with the infection of the EPCs alone. We compared by using different techniques, such as PCR, indirect immunofluorescence (IIF) and fluorescence in situ hybridization (FISH).

RESULTS
In the MSCs-EPCs co-culture system, we identified two sub-fractions of EPCs, one that grew strictly in contact with the adherent MSCs, and the other that grew in suspension over the previous ones. Viral total DNA, RNA and protein expression analysis suggest a diverse infection development in the different conditions, probably due to the grade of interaction between the EPCs and the MSCs.

CONCLUSIONS
The creation of a tissue model reconstructing the human niche of Parvovirus B19 and the characterization of the MSC role in this context will help in understanding the pathogenesis *in vivo*. In addition, our study may have fundamental implications for safety in transplantation biology and cellular therapy involving MSCs.

HYDROGEN PEROXIDE INDUCES APOPTOSIS AND AUTOPHAGY IN MESOANGIOBLAST (C57) CELLS

Roberto Chiarelli[1], Maria Magdalena Barreca[2], Samira Cacciatore[1], Fabiana Geraci[1]
[1]Department of Biological, Chemical and Pharmaceutical Sciences and Technologies, University of Palermo, Palermo, Italy. [2]Department of Biomedicine, Neurosciences and Advanced Diagnostics, University of Palermo, Palermo, Italy

OBJECTIVE

One of the limiting factors in the use of stem cells in regenerative medicine is their high mortality rate during the first days post-transplantation. Indeed, the microenvironment within damaged tissues is hostile for stem cell survival and hydrogen peroxide (H_2O_2) may play a relevant role in inducing death of the injected cells. H_2O_2 is well known as a cell damaging agent that is produced during normal cell metabolism of aerobic organisms. The aim of our study was to determine the mechanism of mesoangioblast (C57) cell death after an H_2O_2 treatment.

MATERIAL AND METHODS

Several assays were used to test the effects of H_2O_2 on mouse mesoangioblast stem cells (C57) to study the activation of apoptotic and autophagic pathways.

In particular, FACS analysis with annexin V/cytox green were used to determine the percentage of early apoptotic-, late apoptotic/necrotic-, and necrotic-cells. Caspase 8, 9, 3/7 activity were determined to highlight the apoptotic pathway involved. Terminal deoxynucleotidyl transferase dUTP nick end labeling (TUNEL) assay was carried out to investigate DNA fragmentation. Mitochondrial membrane potential was measured by FACS with the JC-1 dye.

Autophagy was detected by the C57 transfection with an LC3-GFP plasmid, acridine orange staining and western blot analysis using anti-beclin-1, LC3, p62/SQSTM1, Atg7 and Atg5 antibodies.

RESULTS

We have demonstrated that H_2O_2 induced apoptosis in C57 cells in a time-dependent way, involving both intrinsic and extrinsic pathways. Furthermore, H_2O_2 induced DNA fragmentation and alteration of mitochondrial membrane potential. H_2O_2 also promoted both early and late autophagy

CONCLUSIONS

This preliminary data seems to demonstrate that C57 treatment with H_2O_2 induced both autophagy and apoptosis. Further analysis will be necessary to investigate the possible interaction between the two pathways. A better understanding of why stem cells die after transplantation will help their use in regenerative medicine.

The activity of conditioned medium from amniotic mesenchymal stromal cells and amniotic membrane varies depending on the types of ovarian cancer cells.

Paola Chiodelli[1], Patrizia Bonassi Signoroni[2], Silvia De Munari[2], Jacopo Romoli[1], Andrea Papait[1], Antonietta Silini[2] and Ornella Parolini[1,3].
[1] Università Cattolica del Sacro Cuore, Department of Life Science and Public Health, Rome, Italy.
[2] Fondazione Poliambulanza Istituto Ospedaliero, Centro di Ricerca E. Menni, Brescia, Italy.
[3] Fondazione Policlinico Universitario "Agostino Gemelli" IRCCS, Rome, Italy.

OBJECTIVE
Ovarian cancer accounts for more deaths than any other cancer of the female reproductive system. Nowadays surgical resection followed by chemotherapy is the standard of care. However, a number of patients are faced with recurrence due to tumor dissemination and acquired chemoresistance. Therefore, novel alternative approaches are urgently needed.
Human amniotic membrane (hAM) and mesenchymal stromal cells isolated thereof (hAMSC), have been proposed for several clinical applications, including cancer therapy. Nowadays, it is becoming more evident that the actions exerted by hAMSC are mediated by bioactive factors released during culture (conditioned medium, CM).
This work aims to evaluate the potential anti-tumor effects of CM from hAMSC (hAMSC-CM) and hAM (hAM-CM) on ovarian cancer cells *in vitro.*

MATERIALS AND METHODS
hAMSC-CM and hAM-CM were collected after 5 days in culture and their effect on ovarian cancer cell (HEY, SKOV-3, OV-90) viability was tested with CyQUANT and clonogenic assays. Apoptosis was determined using PI/Annexin V staining and flow cytometry analysis. The migration rate of cancer cells was assessed by wound healing and transwell assays. Moreover, CM-treated cells were also tested for their ability to adhere on extracellular matrix proteins.

RESULTS
Both hAMSC-CM and hAM-CM inhibited cancer cell proliferation and increased apoptosis in a concentration-dependent manner, and with varying potencies depending on the specific cell line. Furthermore, both hAMSC-CM and hAM-CM inhibited HEY and OV-90 colony formation, migration, and adhesion to fibronectin and collagen. Interestingly, hAMSC-CM has an opposite effect on SKOV-3 with respect to hAM-CM, whereby it increases SKOV-3 colony formation, migration, and adhesion on fibronectin and collagen.

CONCLUSIONS
The data collected so far suggest that hAMSC-CM and hAM-CM have different effects on ovarian cancer cells, depending on the specific cell line. The results suggest that CM may affect the migratory capacity, potentially through alterations of integrin expression and/or activity. Further experiments are needed to clarify their mechanism of action on ovarian cancer cells and to clarify their use as a potential adjuvant therapeutic strategy able to target tumor cells.

THE MESENCHYMAL STROMAL CELLS INCREASED THE ACTIVITY ON GLIOBLASTOMA OF A NEW DELIVERED ANTICANCER PLATINUM-DRUG

Coffetti Giulia[2], Valentina Coccè[1], Eleonora Martegani[1], Luisa Doneda[1], Isabella Rimoldi[2], Giorgio Facchetti[2], Giulio Alessandri[1], Emilio Ciusani[3], Francesco Cilurzo[2], Silvia Franzè[2], Francesca Paino[1], Augusto Pessina[1]

[1] CRC StaMeTec, Department of Biomedical, Surgical and Dental Sciences, University of Milan, 20122 Milan, Italy.

[2] CRC StaMeTec, Department of Pharmaceutical Science, University of Milan, Via Mangiagalli 25, 20133 Milan, Italy.

[3] Laboratory of Clinical Pathology and Medical Genetics, Istituto Neurologico Fondazione C. Besta, Milan, Italy.

OBJECTIVE

Glioblastoma multiforme (GBM) is nowadays the most aggressive tumor affecting brain in adults with a very poor prognosis due to limited therapies and systemic cytotoxicity. Among the different new drugs, recently has been reported the in vitro anti-glioma activity of a new cationic platinum(II) complex bearing 8-aminoquinoline as chelating ligand (Pt-8AQ The purpose of this research was to confirm the activity of Pt-8AQ on U87-GM spheroid and to investigate the ability of Mesenchymal Stromal Cells (MSCs) to incorporate and release Pt-8AQ in active form.

MATERIALS AND METHODS

Dosages of 20 μM were employed for the inhibition of the spheroid formation and 5 μM of the drug, added to pre-formed spheroids, produced a significant dramatic degradation after 96 h of treatment. The MSCs were primed with Pt-8AQ in optimized conditions and the secretome was analyzed for the activity of Pt-8AQ and the presence of Evs.

RESULTS

We confirm our previous results in traditional 2D cell culture by demonstrating the anticancer activity of Pt-8AQ against U87-GM cells grown as 3D multicellular spheroids. The principal results showed that Pt-8AQ incorporated by MSCs was released in the secretome and exerted a significant anticancer higher in comparison to free drug's one. The release of Pt-8AQ did not occur in Evs, as found with other drugs, but could be delivered bound to some particular carrier able to enhance its bioavailability and efficacy. Some hypotheses are discussed to explain this surprisingly finding that however will need more investigations.

CONCLUSIONS

The major conclusions are that cell mediated drug delivery systems could provide a potential approach to facilitate the GMB therapy by intra-tumoral administration of cells loaded with Pt-8AQ being MSCs able to integrate into the tumor mass and exert high therapeutic efficacy in situ. The increased efficacy of Pt-8AQ delivered by MSCs even suggests to deeper investigate a possible direct use of MSCs secretome both in situ and/or by systemic administration being secretome able to pass the blood-brain tumor.

(YIA) In vitro modelling of osteoarthritis to disclose the modulation of Connexin 43 in health and disease

Elena Della Morte[1], Chiara Giannasi[1,2], Francesca Cadelano[1,2,3] and Anna Teresa Brini[1,2]
[1]IRCCS Istituto Ortopedico Galeazzi, Milan, Italy
[2]University of Milan, Department of Biomedical Surgical and Dental Sciences, Milan, Italy
[3]PhD student in Experimental Medicine, University of Milan, Milan, Italy

OBJECTIVE

Osteoarthritis is the most common joint disorder that affects millions of people and it is characterized by progressive destruction of the joint's articular cartilage and resultant inflammation. This subsequently leads to stiffness, swelling, pain and loss of mobility. There is no current cure for OA, the treatments are focused on pain reduction until a surgical procedure is needed. Recently, it was demonstrated that Connexin 43 (Cx43), the major component of hemichannel and Gap Junction, is involved in the maintenance of chondrocytes phenotype by interacting with a wide repertoire of intracellular proteins mainly through its C-terminal domain. The aim of our study is to investigate:1. Cx43-modulation by TNFα and IL-1β in articular chondrocytes in comparison with synovial fluid treatment 2. its possible role in the early steps of osteoarthritis development.

MATERIALS AND METHODS

Primary articular chondrocytes (CHs) were isolated from femoral head of patients who underwent total hip replacement. Cells were employed at the first passage, when they reached the confluence were stimulated with TNFα (10ng/ml) and/or IL-1β (1ng/ml) for 1 and 3 days. Cells were also treated with synovial fluid from osteoarthritic patients for 3 days. At the time points, Cx43, MMP-3, MMP-13 and COX2 expression was evaluated by rtPCR and western blotting. The functionality of the channels was displayed with scrape loading assay and the subcellular localization of Cx43 with immunofluorescence.

RESULTS

After 1 day, the cytokines activate the catabolism and the inflammation in chondrocytes increasing genic expression of the MMPs and COX2 in a synergistic manner. The protein expression of Cx43 is strongly downmodulated both by TNFα and synovial fluid in CHs after 3 days but not with IL-1β. The inhibition of the channel can be observed only when Cx43 expression is strongly affected by the treatments. Immunofluorescence shows that other than being present at membrane level Cx43 can be found also in the nucleus.

CONCLUSIONS

The stimulation of articular chondrocytes with two cytokines strongly involved in the onset of osteoarthritis has conflicting effects on the modulation of Cx43 expression. The exposition for 3 days with synovial fluids, that physiologically lubricates cartilage, has a similar effect to TNFα. Further studies investigating the dynamics of Cx43 modulation under different conditions are needed to better understand its role in the physiopathology of cartilag

(YIA) Pleiotropic role of IL6 in affecting PD-L1 expression in hDPSCs and hSFs exposed to inflammatory conditions

Rosanna Di Tinco[1], Giulia Bertani[1], Giulia Orlandi[1], Alessandra Pisciotta[1], Laura Bertoni[1], Elisa Pignatti[1], Monia Maccaferri[1], Paola Sena[1], Stefania Croci[2], Martina Bonacini[2], Alessandro Rossi[2], Jessika Bertacchini[1], Anke Roelofs[4], Cosimo De Bari[4], Carlo Salvarani[2,3], Gianluca Carnevale[1]
[1]Department of Surgery, Medicine Dentistry and Morphological Sciences with Interest in Transplant, University of Modena and Reggio Emilia, Modena, Italy.
[2]Clinical Immunology, Allergy and Advanced Biotechnologies Unit, Azienda Unità Sanitaria Locale-IRCCS di Reggio Emilia, Reggio Emilia, Italy.
[3]Rheumatology Unit, Azienda Unità Sanitaria Locale-IRCCS di Reggio Emilia, Reggio Emilia, Italy.
[4]Arthritis and Regenerative Medicine Laboratory, Aberdeen Centre for Arthritis and Musculoskeletal Health, Institute of Medical Sciences, University of Aberdeen, Aberdeen, UK

OBJECTIVE

Human dental pulp stem cells (hDPSCs) exposed to an *in vitro* inflammatory microenvironment exert immunomodulatory properties through the expression of FasL and the immune checkpoint PD-L1.
Moreover, in the same inflammatory conditions, hDPSCs are also able to up-regulate IL6 which was demonstrated to strongly correlate with PD-L1. Since these two molecules are involved in autoimmune disorders, such as rheumatoid arthritis (RA), the aim of the present study was to investigate the correlation between PD-L1 and IL6 in physiological and pathological conditions by using mesenchymal stromal/stem cells (MSCs) and progenitor cells, such as hDPSCs and human synovial fibroblasts (hSFs), respectively.

MATERIALS AND METHODS

Human DPSCs and hSFs from healthy subjects were exposed to a fine-tuned *in vitro* inflammatory microenvironment by using activated anti-CD3/CD28 PBMCs. The PD-L1/IL6 axis was investigated through RT-PCR, WB and IF by stimulating or inhibiting IL6 pathway. The PD-L1 expression was also studied *in vivo* in the antigen-induced arthritis (AIA) mouse model.

RESULTS

Our data pointed out that hDPSCs up-regulate both PD-L1 and IL6 after exposure to the inflammatory microenvironment. Particularly, it was unveiled that IL6 affect PD-L1 expression through post-transcriptional mechanisms suggesting how stem cells take advantage of IL6/PD-L1 correlation to modulate the inflammatory milieu besides maintaining their stemness phenotype. The IL6-mediated PD-L1 expression was also detected in hSFs underlying how these cells are able to modulate the inflammatory microenvironment through the activation of PD1/PD-L1 pathway. However, preliminary *in vivo* analyses demonstrated that PD-L1 expression was undetectable in inflamed synovium, suggesting that SFs lose this capability after the onset of inflammatory arthritis, even though the inflammatory infiltrate still expressed PD1. Further studies involving hSFs from RA patients are ongoing to validate these preliminary results.

CONCLUSIONS

In the present study, it has been investigated the involvement of IL6 in the immunomodulatory properties of MSCs and progenitor cells. Particularly, it was demonstrated that IL6 is able to induce PD-L1 expression in hDPSCs and hSFs exposed to inflammatory conditions, supporting the pleiotropic role of IL6.

(YIA) Induced pluripotent stem cell-derived 3D Brain organoids cultured in a dynamic bioreactor as an *in vitro* model for the study of microcephaly in Aicardi Goutières Syndrome.

Ferraro Rosalba Monica[1], Manganelli Michele[1], Mazzoldi Elena Laura[1], Parigi Marta[1], Bugatti Mattia[2], Galli Jessica[3], Cattalini Marco[4], Piovani Giovanna[5], Poliani Pietro Luigi[6], Vermi William[6], Fazzi Elisa[3], Giliani Silvia[1].
[1] "Angelo Nocivelli" Institute for Molecular Medicine. Department of Molecular and Translational Medicine, University of Brescia, Italy.
[2] ASST Spedali Civili, University of Brescia, Brescia, Italy.
[3] Child Neurology and Psychiatry Unit, ASST Spedali Civili, Brescia, Italy. Department of Clinical and Experimental Sciences, University of Brescia, Italy.
[4] Pediatric Clinic. ASST Spedali Civili, Brescia, Italy. Department of Clinical and Experimental Sciences, University of Brescia, Italy.
[5] Biology and Genetics Division. Department of Molecular and Translational Medicine, University of Brescia, Italy.
[6] Pathology Unit. ASST Spedali Civili, Brescia, Italy. Department of Molecular and Translational Medicine, University of Brescia, Brescia, Italy.

OBJECTIVE

Neurological disorder is the kind of genetic disease that can benefit the most from 3D modelling for its capability to generate an organized neuronal and glial network, otherwise only available from post-mortem samples. We exploited this possibility to create a 3D neural *in vitro* model of disease to investigate the Aicardi Goutières Syndrome (AGS). AGS is a severe neuro-inflammatory disorder with onset in early infancy. AGS patients exhibit psychomotor retardation, and microcephaly with demyelination and calcification. To date, 9 genes have been identified responsible of the disease.

MATERIALS AND METHODS

We have deepened the *in vitro* 2D neuronal differentiation, generating and characterizing NSCs and neurons from iPSCs of three patients, mutated in: *RNaseH2B, IFIH1, and TREX1*. We didn't observe significant differences between AGS and control-derived neurons in terms of gene and protein expression of typical markers. As one of the features of AGS is the profound microcephaly, we generated iPSC-derived cortical cerebral organoids using a bioreactor that let organoids grew in a dynamic suspension as a better *in vitro* model to explore the cytoarchitectural alteration of the disease. Mini-brains were generated from optimizing the protocol described by Lancaster. The workflow consists in iPSC-derived embryoid bodies generation, neuroectodermal induction, matrix embedding for the neuroepithelium expansion, and cerebral organoids maturation. After 6 weeks of maturation the resulting mini-brains were analyzed in terms of dimension, shape, and expression of neuro-markers by qPCR and immunohistochemical analysis.

RESULTS

The presence of rosette-like structures, typical of cortical-like regions, containing neuroepithelial stem cells (PAX6+) organized into polarized radial structures with a lumen was evaluated. More mature organoids also displayed the presence of cells expressing cortical and glial layer markers (DCX+/ Synaptophysin+/GFAP+). Neural rosettes were positive for the expected markers in all samples and resemble the folding structure of the cerebral ventricles. In particular, AGS organoids showed a smaller size and irregular shape in comparison to control.

CONCLUSIONS

Since to date the description of AGS iPSCs-derived brain organoids is documented only for *TREX1* mutated-samples, we expanded the study cohort to investigate the pathogenetic contributions and interaction between neurons and glia also in other AGS-derived brain organoids characterized by specific genetic mutations.

Microplastics and pregnancy: investigating the biological effects of bisphenol A on mesenchymal stromal cells of the amniotic membrane

Sara Ficai[1*], Andrea Papait[1,2*], Marta Magatti[3], Elsa Vertua[3], Antonietta Silini[3], Ornella Parolini[1,2]
1 Department of Life Science and Public Health, Università Cattolica del Sacro Cuore, Rome, Italy
2 Fondazione Policlinico Universitario "Agostino Gemelli" IRCCS, Rome, Italy
3 Centro di Ricerca E. Menni, Fondazione Poliambulanza Istituto Ospedaliero, Brescia, Italy.
*The authors equally contributed to this study

OBJECTIVE
Microplastic contamination has emerged as a critical environmental concern with potential health implications for humans. Notably, recent discoveries have unveiled the presence of microplastics in various reproductive-related contexts, such as placenta, breast milk, and infant urine. Among the inevitable by-products of microplastic degradation, bisphenol A (BPA) has gained attention for its toxicity in female reproductive health. These findings suggest that the effects of microplastics and associated chemicals might extend beyond adults and potentially impact babies, influencing crucial stages of gestation. The amniotic membrane, which constitutes the outermost membrane separating the fetus from the mother, plays an essential role during pregnancy. Our study aimed to explore the effects of BPA on mesenchymal stromal cells isolated from the amniotic membrane (hAMSCs), with the primary goal of identifying the specific pathways that are disrupted by BPA exposure.

MATERIAL AND METHODS
hAMSC were isolated from full-term deliveries with prior informed consent. The isolated cells were expanded to the first passage and then exposed to increasing concentrations of BPA for 24 hours. Subsequently, hAMSCs were collected to assess the impact of BPA on their viability (MTT, ATPlite, positive staining with eFLUOR probe), apoptosis (PI-AnnV staining), production of reactive oxygen species (ROS) (positive staining with Mitosox RED probe). The stabilization of p53 and activation of the inflammasome were assessed through molecular analysis of key genes including NLRP3, NLRC4, AIM2, ASC, caspase 1, and IL-1β.

RESULTS
Our results demonstrated that increasing concentrations of BPA lead to decreased cell viability in a dose-dependent manner, while inducing the production of oxidative species, the stabilization of p53, and the activation of apoptosis in hAMSC. On the other hand, we hypothesized that ROS production can induce the activation of inflammasome as highlighted by the increased expression of molecules forming the inflammasome complex.

CONCLUSIONS
Understanding the mechanisms through which BPA exerts its effects is a significant step toward comprehending the detrimental impact of environmental pollution on pregnancy progression and outcomes. These findings emphasize the need for further research and awareness regarding the potential risks posed by microplastic contamination and associated chemicals, particularly in the context of pregnancy.

AMNIOTIC MESENCHYMAL EXTRACELLULAR VESICLES FOR EQUINE CHRONIC DEGENERATIVE ENDOMETRITIS (CDE) THERAPY

Giulia Gaspari[1], Federico Funghi[2], Carlo Cantile[3], Fausto Cremonesi[1], Anna Lange-Consiglio[1]

[1]Department of Veterinary Medicine and Animal Science (DIVAS), Laboratoy of Reproduction and Rigenerative Medicine, Università degli Studi di Milano, Via dell'Università, 6, 26900 Lodi, Italy.
[2]Private practitioner, 58100 Grosseto, Italy.
[3]Department of Veterinary Science, Università di Pisa, Viale delle Piagge 2, 56124 Pisa.

OBJECTIVE
Equine chronic degenerative endometritis (CDE) is recognized as a major cause of subfertility in mares. CDE is a progressive process characterized by fibrosis of endometrium with morphological and functional alterations that are responsible of modifications of uterine environment. Peri-implantation stage is a critical phase, in which the proper interaction between embryo and maternal counterparts is essential. This communication is guaranteed by paracrine signaling among cells, and extracellular vesicles (EVs) are recognized to play a pivotal role in this mechanism. Since fetal-maternal communication is altered in mares affected by CDE, the aim of this study was to try to restore this communication by intrauterine infusions of amniotic-derived extracellular vesicles (AMC-EVs).

MATERIALS AND METHODS
Three at term placentas were obtained following spontaneous vaginal delivery. Amniotic derived cells (AMCs) were isolated and cultured until passage three, then were maintained in serum-free medium for one night to collect conditioned media (CM) that was ultracentrifuge at 100,000× g at 4°C for 1 h. EVs were characterized for size and concentration by nanoparticle tracking analysis and for markers via Western blotting. Transmission electron microscopy was also performed.
Mares enrolled in this study (n=12) were selected based on a diagnosis of CDE associated with infertility. Endometrial biopsies were performed to evaluate histologically the severity of endometritis. All mares received two intrauterine infusions of $20x10^9$ EVs diluted in 50 ml of sterile saline solution, at day 5 and day 9 after ovulation estimated by ultrasound. At the following ovulation, all mares were artificially inseminated with fresh semen.

RESULTS
On a total of n=12 CDE-affected mares enrolled in this study, 11 mares had conception and to date 8 mares were able to carry the pregnancy to term after the treatment with AMC-EVs. Interestingly, the only mare that couldn't establish a conception was a mare with an altered perineal conformation that exposed her to a higher risk of uterine infections, that might have contributed to the failing outcome.
The histological condition of the endometrium of all mares did not show any improvement, meaning AMC-EVs did not exert a regenerative activity.

CONCLUSIONS
The AMC-EV administration might have contributed to re-establish a functional paracrine interaction between embryo and maternal tissues, leading to successful pregnancies.

(YIA) ISOLATION AND PRELIMINARY CHARACTERIZATION OF MESENCHYMAL STROMAL CELLS FROM ADIPOSE TISSUE OF CHELONIAN TRACHEMIS SCRIPTA.

Martina Gavezzoli (1), Valentina Andreoli (1), Priscilla Berni (1), Alessandro Vetere (1), Virna Conti (1), Francesco di Ianni (1), Stefano Grolli (1).
(1) Department of Veterinary Science, University of Parma, 43100 Parma, Italy

OBJECTIVE

Mesenchymal stromal cells (MSCs) are of strong clinical interest in veterinary regenerative medicine. Notably, little is known about these cells in chelonians and other reptile species considered of importance as endangered species or as non-conventional pets. Recently, isolation of bone marrow derived MSCs-like cells has been reported in the amphibian *Xenopus la*evis. The aim of this work was to evaluate the feasibility of the isolation of adipose tissue-MSCs from a model chelonian, *Trachemis Scripta*.

MATERIALS AND METHODS

Adipose tissue samples (n=5) were collected during elective surgery aimed at *T.scripta* sterilization. Biopsies (0.5-1 gr) were collagenase-digested or alternatively tissue micro-fragments were glued on plastic surface by means of plasma gel droplets. Cultures were performed in DMEM high glucose supplemented with 10% FBS at 28°C, in a humidified atmosphere with 5% CO_2. Cell growth rate was characterized (P1 to P8) by direct cell counting. Multi-differentiative potential was assessed by inducing adipogenic, osteogenic and chondrogenic differentiation. Cell phenotype was characterized by RT-PCR and amplicon sequencing of a panel of markers routinely used for mammalian MSCs characterization. Blood cells were used to validate primers for genes not expressed in MSCs. Furthermore, CFU forming ability was evaluated in low density cell cultures.

RESULTS

T.scripta MSCs maintain constant replicative rate from P1 to P8 showing lower replicative capacity than MSCs from mammalian species, but also from *X. laevis*. Cells stimulated by differentiation mediums typically used for mammalian MSCs were able to differentiate in an adipocytic, chondrogenic, and osteogenic phenotype. Gene expression analysis performed by RT-PCR demonstrated that the cells express CD105, CD73, CD44, CD90 but do not express CD34 and HLADRA. Amplicons were validated by direct sequencing, giving a mean homology >98% with sequences reported for *T. Scripta* genome.

CONCLUSION

Experimental data collected from cultures of stromal cells derived from adipose tissue of *T. scripta* strongly suggest that the isolation of MSCs-like cells from chelonian is feasible. Adequate numbers of cells suitable for clinical application were available in 2-3 weeks. *T.scripta* MSCs cells recapitulate most of the basic characteristics of mammalian MSCs. Regenerative therapy in reptiles is a quite new field but promises interesting possibilities both for pet patients and wild endangered species.

Effect of Metformin on Human Neural Stem Cells: How to Improve Their Therapeutic Potency

Valentina Grespi[1], Rita Romani[2], Ilaria Bellezza[2], Davide Chiasserini[2], Magdalena Davidescu[2], Lara Macchioni[2], Letizia Mezzasoma[2], Gianmarco Muzi[1] and Carlo Conti[3]

[1]Stem Cells Laboratory, Cell Factory and Biobank, "S.Maria" Hospital, Terni, Italy
[2]Department of Medicine and Surgery, University of Perugia, Perugia, Italy
[3]Neuroscience Department, "S.Maria" Hospital, Terni, Italy

OBJECTIVE

Human Neural Stem Cells (hNSCs) treatment for neurodegenerative and neuroinflammatory diseases might exert antagonizing effects on inflammation and neurodegeneration. Metformin exhibits strong anti-aging properties and promote adult neurogenesis under physiological and pathological conditions. The aim of the study was to understand whether metformin could promote proliferation, self-renewal and neuronal differentiation of clinical grade hNSCs in order to optimize their therapeutic effect.

MATERIALS AND METHODS

hNSCs lines used for this study derived from brain biopsies of fetuses from spontaneous miscarriage or natural in uterus death. The lines have been reproducibly and stably expanded *ex vivo* by means of a production protocol adherent to GMP and authorized by the Italian Medicine Agency (AIFA). Cells were cultivated for 30 days in a chemically defined medium with or without metformin supplementation at 500nM and 1μM. For each condition we evaluated specific markers for hNSCs (Ki-67, Olig2, GFAP), self-renewal potential, by calculating the growth kinetics during *in vitro* culture, and clonogenic capacity, estimated using the clonal efficiency assay. Oxygen consumption was determined using Clark's electrode. The *in vitro* differentiation test was used to assess the ability of the cells to give rise to astrocytes, neurons and oligodendrocytes by immunocytochemical specific labeling (β-tubulin III, GFAP, GalC). Finally, we evaluated inflammasome activation in THP1 monocyte cells by hNSCs-derived extracellular vesicles (hNSCs-EVs) treated or not with metformin.

RESULTS

We observed that, compared to the control, metformin significantly increases cell proliferation rate and viability. Metformin, at both concentrations, robustly increases the number and the size of neurospheres, indicators of self-renewal and proliferation, and significantly enhanced neuronal differentiation. Metformine-treated hNCSs did not show changes in O_2 consumption. In addition, we found that THP-1 cells treated with hNCSs-EVs from control and from metformin treated cells failed to activate the NLRP3/caspase-1/inflammasome pathway in response to LPS and ATP treatment.

CONCLUSIONS

Our findings provide evidence that metformin treatment produces sustained hNSCs self-renewal and proliferation responses. The identification of molecular pathways regulated by metformin, as well as the effect of this drug on hNSCs-EVs biological properties will be the subjects of future evaluations.

Robust and reproducible GMP-compliant protocol to manufacture human inducted pluripotent stem cells

Salvatore La Rosa[1], Jessica Baldi[1], Emilio Carrino[1], Claudio Prencipe[1], Cristiana Lavazza[1], Silvia Budelli[1], Elisa Montelatici[1], Francesco Rusconi[1], Valeria Peli[1], Silvia Marasco[1], Alejandro Espadas De Arias[3], Rosalba Monica Ferraro[2], Silvia Clara Giliani[2], Lorenza Lazzari[1]

[1]Unit of Cell and Gene Therapies, Fondazione IRCCS Ca' Granda Ospedale Maggiore Policlinico, Milano, Italy
[2]A. Nocivelli Institute for Molecular Medicine, Department of Molecular and Translational Medicine, University of Brescia, Italy
[3]Laboratory of Transplant Immunology SC Trapianti Lombardia – NITp, Fondazione IRCCS Ca' Granda Ospedale Maggiore Policlinico, Milano, Italy

OBJECTIVE

Human induced pluripotent stem cells (hiPSC) emerged as an attractive tool for regenerative medicine due to their potential to differentiate into cell types. The hiPSC application demands adherence to stringent quality and safety standards, governed by Good Manufacturing Practices (GMP). These regulations ensure the reproducibility and safety of hiPSC-derived therapeutic products, enhancing their translational potential. The goal of our work was to obtain stable and safe clones to create a starting material to manufacture human neural progenitor cells and beta cells for future clinical applications.

MATERIALS AND METHOD

After Ethics Committee approval (EC approval 540/2021) and signed informed consent, we isolated, expanded and reprogrammed peripheral mononuclear cells from blood samples of a healthy female blood donor (age 33yrs) carrying homozygous HLA alleles (A*02:01, B*50:01, DR*07:01). Given the safety concerns surrounding the integrating viral vectors, a non-integrative episomal-based technology was used for reprogramming. The matrix usually used for iPSC is extracted from a mouse sarcoma, therefore its applicability to therapeutic cell manufacturing is limited. Consequently, we established optimal iPSC culture conditions based entirely on recombinant substrates. Different GMP-compliant media were tested to optimize the culture conditions and to allow the best expansion of the transduced cells. Optimal quality controls were defined including the flow cytometry analysis for the pluripotency genes and the assess of pluripotent marker expression. hiPSC colonies started to appear from day 13. At passage 15 hiPSC clones showed expression of OCT4 and of SOX2 higher than 80% and 90%. The expression of SOX2, OCT4, LIN28, NANOG was confirmed by qPCR. Karyotype assay resulted in normal genomic profile validating the hiPSC stability during passages, and the STR test confirmed the identity of the donor. Trilineage cell differentiation defined the ability of GMP-compliant hiPSC lines to differentiate toward mesoderm, endoderm and ectoderm. The viral copy number residue procedure was validated. Batch records for reagents, materials and activities were drawn up.

CONCLUSIONS

hiPSC lines were effectively generated through a robust, reproducible, and GMP-compliant manufacturing process, employing top-tier practices for cell culture, documentation, and quality control.

(YIA) Ultrasound stimulation reduces inflammation and promotes the chondrogenic differentiation of adipose mesenchymal stromal cells embedded in a 3D piezoelectric hydrogel

Enrico Lenzi[1], Cristina Manferdini[1], Elena Gabusi[1], Paolo Dolzani[1], Yasmin Saleh[1], Diego Trucco[1,2,3], Giovanni D'Atri[1], Marta Columbaro[4], Lorenzo Vannozzi[2,3], Andrea Cafarelli[2,3], Leonardo Ricotti[2,3], Gina Lisignoli[1]
[1] IRCCS Istituto Ortopedico Rizzoli, SC Laboratorio di Immunoreumatologia e Rigenerazione Tissutale, Bologna, Italy;
[2] The BioRobotics Institute, Scuola Superiore Sant'Anna, Pisa, Italy;
[3] Department of Excellence in Robotics & AI, Scuola Superiore Sant'Anna, Pisa, Italy;
[4] IRCCS Istituto Ortopedico Rizzoli, Piattaforma di Microscopia Elettronica, Bologna, Italy.

OBJECTIVE

In cartilage tissue engineering (TE) new solutions are needed to effectively drive chondrogenic differentiation of adipose mesenchymal stromal cells (ASCs) both in normal and inflammatory milieu. Hydrogels are promising biomaterials capable of embedding ASCs by providing an instructive biomimetic environment which can be stimulated by low-intensity pulsed ultrasound (LIPUS) to regulate cell differentiation. Graphene oxide (GO) thanks to its chondroinductive properties when embedded into polymeric formulations and barium titanate piezoelectric nanoparticles (BTNPs), usable as nanoscale transducers, represent promising nanomaterials for cartilage TE. The aim of this study was to evaluate the effect of dose-controlled LIPUS in reducing inflammation and positively committing the chondrogenesis of ASCs embedded in a 3D piezoelectric hydrogel.

MATERIALS AND METHOD

ASCs at $2x10^6$ cells/mL were embedded in a 3D VitroGel RGD® hydrogel without nanoparticles (Control) or doped with 25 μg/mL of GO nanoflakes and 50 μg/mL BTNPs. The hydrogels were exposed to basal or inflammatory milieu (+IL1β 10 ng/mL), stimulated with LIPUS every 2 days until day 10 of culture and chondrogenic differentiated for 28 days.

At day 2, 10 and 28 cell viability, cytotoxicity, gene expression and immunohistochemistry (COL2, aggrecan, SOX9, and COL1) were evaluated. Inflammatory cytokines (IL6, CXCL8, TNF-α, CCL2, CCL4 and CCL5) were also analysed.

RESULTS

In basal condition, LIPUS considerably push the chondrogenic differentiation of ASCs embedded in 3D piezoelectric hydrogel, in particular COL2 and aggrecan were overexpressed, while the fibrotic marker COL1 decreased compared to control samples. LIPUS also has effective anti-inflammatory effects by down-modulating IL6, CXCL8, TNF-α, CCL2, CCL4 and CCL5, keeping its ability to boost chondrogenesis.

CONCLUSIONS

These results suggest that LIPUS stimulation of piezoelectric hydrogels promotes the chondrogenic differentiation of ASCs by reducing the inflammatory milieu, therefore representing a promising tool in the field of cartilage TE.

Acknowledgments

This work received funding from the European Union's Horizon 2020 research and innovation program, grant agreement No 814413, project ADMAIORA (AdvanceD nanocomposite MAterIals fOr in situ treatment and ultRAsound-mediated management of osteoarthritis).

YIA Tailoring a novel colorectal cancer stem-targeted therapy by inhibiting the methyltransferase SMYD3

Martina Lepore Signorile[1], Valentina Grossi[1], Giovanna Forte[1], Candida Fasano[1], Katia De Marco[1], Paola Sanese[1] , Vittoria Disciglio[1], Elisabetta Di Nicola[1], Antonino Pantaleo[1], Cristiano Simone[1,2]

[1] Medical Genetics, National Institute for Gastroenterology, IRCCS 'S. de Bellis', Castellana Grotte (Ba), 70013, Italy

[2] Medical Genetics, Department of Precision and Regenerative Medicine and Jonic Area (DiMePRe-J), University of Bari Aldo Moro, Bari, 70124, Italy

OBJECTIVE

Colorectal cancer (CRC) is a leading cause of cancer-related death, and nearly 50% of affected patients show tumor recurrence, which depends on a small subset of cells within the tumor, called CRC stem cells (CRC-SCs). SMYD3 has been found upregulated in around one-third of CRC patients. Despite being a histone methyltransferase, reports suggest that its oncogenic activity may be mediated by functional interactions with non-histone proteins, through which it transactivates specific pathways involved in the survival of cancer cells. This study intends to investigate the role of SMYD3 in cancer stemness activity.

MATERIALS AND METHOD

I performed extensive characterization of the functional interaction between SMYD3 and CRC-SCs markers in patients derived cancer stem cells. I investigated several CRC-SCs features by gene editing and pharmacological inhibition of SMYD3.

RESULTS

Firstly, I found SMYD3 overexpressed in most of patient-derived CRC-SCs investigated. I performed a peptide screening for new SMYD3-interacting proteins and I identified 66 proteins involved in stem cell biology. Interestingly, this cluster includes 7 out of the 24 genes product identified by Takahashi as candidate factors inducing pluripotency in somatic cells. Furthermore, I performed co-immunoprecipitation assays confirming that SMYD3 interacts endogenously at least with c-MYC, STAT3, and SOX2 in CRC-SCs. Direct involvement of SMYD3 activity in CRC-SC features was suggested by the fact that its pharmacological inhibition caused the downregulation of the expression of various CRC-SC markers. Moreover, SMYD3 inhibition affected CRC-SC typical features as migration and invasion abilities, and decreased cell proliferation index while increasing CRC-SCs death. Moreover, I evaluated cancer SMYD3-KO cells for their sphere-initiating ability, a typical feature of stem cells, and found that they are unable to form tumorspheres. Taken together, these results suggest that SMYD3 is a key factor in the tumorigenesis driven by CRC-SCs and therefore could be used as a molecular target to counteract cancer development.

CONCLUSIONS

I believe that a full comprehension of the functional interplay between SMYD3 and CRC-SC markers will be useful to devise new targeted therapeutic strategies to treat CRC. Given the prevalence of this type of cancer and the severity of its side effects, the identification of new effective treatments is expected to have a major impact at the clinical level.

Systemic immune alterations after traumatic brain injury in young and elderly patients and the conditioned medium from human amniotic mesenchymal stromal cells as a potential therapeutic strategy.

Marta Magatti[1], Anna Pasotti[1], Francesca Pischiutta[2], Fabrizio Ortolano[3], Enrico Caruso[2,3], Anna Cargnoni[1], Andrea Papait[4], Franco Capuzzi[5], Tommaso Zoerle[3,6], Marco Carbonara[3], Nino Stocchetti[3,6], Stefano Borsa[7], Marco Locatelli[6,7], Elisa Erba[8], Daniele Prati[8], Antonietta R Silini[1], Elisa R Zanier[2], Ornella Parolini[4,9]

[1] Centro di Ricerca E. Menni, Fondazione Poliambulanza Istituto Ospedaliero, Brescia, Italy
[2] Laboratory of Traumatic Brain Injury and Neuroprotection, Department of Acute Brain and Cardiovascular Injury, Istituto di Ricerche Farmacologiche Mario Negri IRCCS, Milan, Italy
[3] Neuroscience Intensive Care Unit, Department of Anesthesia and Critical Care, Fondazione IRCCS Ca' Granda Ospedale Maggiore Policlinico, Milan, Italy
[4] Department of Life Science and Public Health, Università Cattolica del Sacro Cuore, Rome, Italy
[5] Dipartimento Medicina di Laboratorio, Fondazione Poliambulanza Istituto Ospedaliero, Brescia, Italy
[6] Department of Pathophysiology and Transplantation, University of Milan, Milan, Italy.
[7] Unit of Neurosurgery, Fondazione IRCCS Ca’ Granda Ospedale Maggiore Policlinico, Milan, Italy
[8] Department of Transfusion Medicine and Hematology, Fondazione IRCCS Ca' Granda Ospedale Maggiore Policlinico, Milan, Italy
[9] Fondazione Policlinico Universitario “Agostino Gemelli” IRCCS, Rome, Italy

OBJECTIVE

Traumatic brain injury (TBI) is a leading cause of mortality and disability among both young and elderly subjects, with the elderly population experiencing the worst outcome. In addition to primary brain damage and neuroinflammation, systemic immune alterations occur and can aggravate TBI outcome and complications.

Cells from the mesenchymal region of the amniotic membrane (hAMSC) and the conditioned medium generated from their culture (CM-hAMSC) act on cells of both innate and adaptive immunity, thus controlling inflammation, proliferation, and cytokines production.

Considering the critical role of the immune system in TBI, and the ability of CM-hAMSC to control inflammation, we aimed to investigate the immunological profile of peripheral blood mononuclear cells (PBMC) in relation to the age of the patients, and the ability of CM-hAMSC to modulate them.

MATERIALS AND METHODS

PBMC were collected acutely (<48h) after TBI in young (18-45 yo) and elderly (>65 yo) patients. PBMC were activated in vitro with CD3/CD28 and cultured in presence of CM-hAMSC. Analyses were performed by flow cytometry.

RESULTS

We observed a distinct response to TBI between elderly and young patients, highlighting an unfavourable immune status of the elderly population. TBI led to a reduction in systemic T cell levels, and age affects their activation and differentiation. Indeed, in young patients, only CD4 T lymphocytes were activated by TBI. However, in elderly patients, both CD4 and CD8 T cells were affected, leading to their differentiation into subtypes with low cytotoxic activity, such as central memory CD4 T cells and memory precursor effector CD8 T cells. TBI increased the percentage of the Th2 subset, with a greater effect in elderly compared to young patients. Both CD4 and CD8 T cells were able to proliferate in response to CD3/CD28, although to a lesser extent than aged-matched healthy controls. While in young TBI patients only CD4 T cells presented a reduced proliferation compared to controls, in elderly TBI patients both CD4 and CD8 showed reduced proliferation upon stimulation. To determine the effect of CM-hAMSC on T lymphocytes, PBMC were stimulated in the presence of CM-hAMSC. Our data outlined that CM-hAMSC suppressed T cell proliferation, with similar effects in healthy controls and TBI patients.

CONCLUSIONS

Our results elucidate the systemic immune changes that occur after TBI in young and elderly patients, and could suggest CM-hAMSC as a possible therapeutic adjuvant in controlling TBI immune alterations.

YIA A new potential therapeutic approach on mesothelioma with mesenchymal stromal cells loaded with Paclitaxel (PacliMES).

Eleonora Martegani[1], Valentina Coccè[1], Mara Bonelli[2], Silvia La Monica[2], Roberta Alfieri[2], Luisa Doneda[1], Giulio Alessandri[1], Costanza Annamaria Lagrasta[2], Aldo Giannì[1,3], Valeria Sordi[4], Francesco Petrella[1,5,6], Costantino Corradini[7], Angelo Guido Corsico[8], Giulia Maria Stella[9], Leda Roncoroni[1], Francesca Paino[1] and Augusto Pessina[1]

[1] CRC StaMeTec, Department of Biomedical, Surgical and Dental Sciences, University of Milan, 20122 Milan, Italy.

[2] Department of Medicine and Surgery, University of Parma, 43126 Parma, Italy.

[3] Maxillo-Facial and Dental Unit, Fondazione Ca' Granda IRCCS Ospedale Maggiore Policlinico, 20122 Milan, Italy.

[4] Diabetes Research Institute, IRCCS San Raffaele Hospital, 20132, Milan Italy

[5]Department of Thoracic Surgery, IRCCS European Institute of Oncology, 20139 Milan, Italy.

[6] Department of Oncology and Hemato-Oncology, University of Milan, 20122 Milan, Italy.

[7]Department of Biomedical Surgical and Dental Sciences, Sports Trauma Researches Center, State University of Milan c/o 1st Division of Orthopedics and Traumatology, Orthopedic Center Pini CTO - ASST Gaetano Pini, Milan, Italy

[8] Department of Internal Medicine and Medical Therapeutics, University of Pavia Medical School, 27100 Pavia, Italy.

[9] Cardiothoracic and Vascular Department, Unit of Respiratory Diseases, IRCCS Policlinico San Matteo, 27100 Pavia, Italy.

OBJECTIVE

Malignant pleural mesothelioma (MPM) is an asbestos-related malignancy originating in the mesothelial cells of the pleura. Different chemo-therapeutic approaches have been tested, but results have generally been disappointing. Adult mesenchymal stromal cells (MSCs) derived from bone marrow (BM) or adipose tissue (AT) have been suggested as suitable cell sources for cell-based therapies. Our group previously demonstrated that both lysates and conditioned media (CM) from AT-MSCs, can inhibit the proliferation of mesothelioma cell lines. Furthermore, our *in vivo* study confirmed that a loco-regional treatment of mesothelioma xenograft with high amounts of AT-MSCs resulted in a dramatic inhibition of tumor growth comparable with that produced by systemic administration of PTX. The aim of this study is to demonstrate the *in vivo* effect exerted on mesothelioma xenografts by “low amount” of MSCs both untreated and primed with PTX (MSCs/PTX).

MATERIALS AND METHODS

The effect of Pemetrexed (PMX) and Paclitaxel (PTX) were compared *in vitro* by an antiproliferation MTT assay on mesothelioma cell line MSTO-211H. AT-MSCs were loaded with PTX according to standardized procedure. The effect of PTX, MSCs and MSCs/PTX on MSTO-211H was analyzed using transwell inserts (2D) and 3D spheroids. Caspase-3 expression in spheroids after PTX treatment was evaluated by RT-qPCR analysis. To evaluate the effect of PTX released by MSCs on cell migration, CM from MSCs/PTX were tested in wound healing assay. *In vivo* experiments were performed in a Balb/c-Nude female mice that were subcutaneously injected with MSTO-211H cells. Mice were intraperitoneal treated with PTX, MSCs or MSCs/PTX. Tumor xenografts were measured three times per week and tumor volume was determined.

RESULTS:

Our experiments confirmed the efficacy of PTX on MSTO-211H in 2D and 3D cultures, and that “low amount” MSCs/PTX inhibit tumor growth at a higher extent then Paclitaxel alone. An *in vivo* approach to treat *in situ*

mesothelioma xenografts using a minimal amount of MSCs/PTX showed the same efficacy of a systemic administration of 10 mg/kg of Paclitaxel.

CONCLUSION

These data support drug delivery system by MSCs as a useful approach against solid tumors. MSCs/PTX as a new Advanced Medicinal Therapy Product (Paclimes) has been approved for a Phase I clinical trial on mesothelioma patients and we hope that the results could pave the way for using MSCs for drug delivery as adjuvant therapy in other solid tumors.

Assessment of Mitochondrial Function in Stem Cells following Oxidative Damage

Deanira Patrone[1], Sura Al-sammarraie[1], Mohd Shahzaib[1], Domenico Aprile[1], Umberto Galderisi[1]
[1] Department of Experimental Medicine, Biotechnology and Molecular Biology Section, University of Campania "Luigi Vanvitelli", Naples, Italy

OBJECTIVE

Mitochondria plays a pivotal role in cellular stress response, serving as both sensors and effectors in maintaining cellular homeostasis during challenging conditions. They are at the crossroads of the cellular stress response, orchestrating various processes to ensure cell survival and maintain overall tissue and organismal health. The ability of mitochondria to regulate energy production, ROS levels, calcium homeostasis, apoptosis and metabolism is essential for the cell's adaptability and resilience in the face of diverse stressors.

Multilineage Differentiating Stress Enduring (Muse) Cells are stress-resistant stem cells capable of surviving and differentiating in damaged tissues. To understand the mechanisms underlying the stress resistance of Muse cells, we focused on the role of mitochondria.

MATERIALS AND METHODS

We induced oxidative stress with H_2O_2 for 30 minutes in Mesenchymal Stromal Cells (MSCs), Muse cells and Non-Muse cells. After 48 hours, we assessed the mitochondrial conditions by evaluating mitophagy, mitochondrial integrity, the expression of mitochondrial small peptides, and mitochondrial biogenesis.

RESULTS

Our preliminary findings regarding the mechanisms of mitochondrial stress resistance in Muse cells show that mitophagy, the process of eliminating damaged mitochondria, increases in Muse cells after stress compared to Non-Muse cells and MSCs. This was demonstrated by immunocytochemistry staining using Mito-tracker, a marker for detecting mitochondria, and Lamp1, a lysosomal marker. The integrity of Muse cell mitochondrial DNA is preserved after H_2O_2 damage, unlike MSCs and Non-Muse cells, as revealed by PCR analysis of both large and small mitochondrial DNA fragments.

Additionally, ICC analysis demonstrated the overexpression of Humanin and MOTS-c, two small peptides encoded in mitochondria, in Muse cells after exposure to oxidative stress. These peptides were found to play intriguing roles in the cellular stress response, particularly in safeguarding mitochondria and enhancing cellular health. Finally, we evaluated mitochondrial biogenesis through western blot analysis of COX-1 and SDHA proteins. Following oxidative stress, the COX1/SDHA ratio was higher in Muse cells, indicating an increase in mitochondrial biogenesis.

CONCLUSIONS

Our preliminary results underscore the fundamental role of mitochondrial regulation in the stress resistance mechanisms of Muse cells, with the aim of further understanding mitochondrial behavior.

A 3D-printed composite scaffold to study periodontal ligament regeneration by PDLSCs and ASCs

Riccardo Pedraza[1,2], Alessandro Mosca Balma[1], Ilaria Roato[1], Federico Mussano[1]

[1]Department of Surgical Science, C.I.R. Dental School, Università di Torino, Italy.

[2]Institute of Science and Technologies for Sustainable Energy and Mobility, National Council of Research, Torino, Italy.

OBJECTIVE

The union of 3D-printing technology and stem cell-based cell biology may be a valid option to build scaffolds capable of supporting tissue regeneration in maxillary surgery. Adipose tissue-derived stem cell (ASC) and periodontal ligament stem cell (PDLSC) behaviors are being investigated on a 3D-printed composite material (CM). Smart bone (SB) filler and polycaprolactone (PCL) matrix are being used to promote bone tissue regeneration.

MATERIALS AND METHODS

Three CM were compared: PCL, PCL/SB 70/30 w/w blend, and SB. The PCL/SB 70/30 blend was created by incorporating milled SB into the polymer matrix through a dry mixing technique with a milling machine. PCL/SB 70/30 blend and PCL were employed in 3D printing, following standard parameters, to fabricate scaffolds for ASC52 hTert stem cell line and human primary PDLSC. Biological and morphological assessments were conducted. Cell viability was carried out using the CellTiter-Glo kit to assess cell proliferation over three time points:3, 7, and 14 days. All materials underwent a 2-month stimulation in osteogenic culture medium to observe in vitro differentiation. Real-Time PCR was employed to evaluate gene expression, focusing on RUNX2, ALP, OCN, and COL1. SEM was used to observe new tissue formation, and EDX analysis was performed to quantify the presence of calcified nuclei like Na2O, P2O3 and CaO within the collagenic matrix.

RESULTS

ASCs demonstrated a significant preference for PCL. Cell proliferation on PCL differed significantly from the one of the other two materials, indeed on SB a rapid activity in terms of adhesion and proliferation were observed for PDLSC. Thus, SB showed a statistically difference from PCL and from PCL/SB on the day 7 only. Real-Time PCR results highlighted a distinct variance between the two cell populations. COL1 gene displayed broad expression across all substrates for both cell types. OCN gene expression of PDLSCs varied significantly from that of ASCs. This discrepancy led to heightened calcification of collagenic matrix by PDLSCs compared to ASCs, a finding further substantiated by EDX analysis and SEM images.

CONCLUSIONS

PCL/SB CM exhibited enhanced biocompatibility and osteoinductivity compared to PCL, and it must be considered in the design of customizable, durable and osteoconductive substrate for tissue regeneration. Subsequent investigations will be directed towards testing other mesenchymal stem cell types and conducting additional biomechanical assessments.

Wharton's Jelly-derived multifunctional hydrogels: new tools for regenerative medicine applications

Letizia Penolazzi[1], Anna Chierici[1], Maria Pina Notarangelo[1], Federica Fortuna[1], Elisabetta Lambertini[1], Roberta Piva[1], Claudio Nastruzzi[2]
[1] Department of Neuroscience and Rehabilitation, University of Ferrara, 44121 Ferrara, Italy
[2] Department of Chemical, Pharmaceutical and Agricultural Sciences, University of Ferrara, 44121 Ferrara, Italy

Human Wharton's jelly (WJ) has a great potential for regenerative medicine applications as "natural constituent" to produce biomimetic scaffolds. WJ is rich in extracellular matrix (ECM) components (hyaluronic acid, various types of collagens and glycosaminoglycans), growth factors (FGF, IGFBP 1, 2, 3, 4, and 6, TGFα, GH and PDGF-AA), immunomodulators (RANTES, IL-6R, and IL-16), pro- and anti-inflammatory (MCSFR, MIP-1a, TNF-RI, TNF-RII, and IL-1RA) and homeostatic cytokines (TIMP1 and TIMP2), cytokines associated with wound healing (ICAM-1, G-CSF, GDF-15, MCP-1, GDNF) and extracellular vesicles. Proper and controlled cell removal procedures produce the decellularized WJ (DWJ) which maintains the abundance of all these factors, providing a biocompatible structure capable of supporting many reparative processes, although many questions remain open regarding the fabrication and usability of DWJ-based scaffolds.

OBJECTIVE

we aimed to produce scaffolds with a tailored architecture and mechanical properties closest to those of the specific tissue, without losing the ability to support cell proliferation, differentiation, ECM synthesis and remodeling.

MATERIALS AND METHODS

We prepared and characterized DWJ based multifunctional hydrogels, in form of 3D millicylinders (WJM), composed of a mixture of alginate, gelatine and DWJ (at various %). Different aspects were considered, including, the setup of the decellularization protocol, the usability and conservation of the devices, and finally the ex-vivo biological evaluation. Degenerated intervertebral disc (IVD) cells isolated from lumbar disc tissues of patients undergoing surgical discectomy were used as an experimental model.

RESULTS

The developed protocol, based on a freezing step, leads to the consolidation of the entire polymeric dispersion mixture, followed by an ionic gelation step. After gelation of the freshly prepared millicylinders, the samples were subjected to a freeze-drying process, resulting in a porous, stable, easily storable and suitable matrix for ex-vivo experiments.
WJMs with the highest percentage of DWJ were effective in supporting cell migration, restoration of the IVD phenotype (increased Collagen type 2, aggrecan, Sox9 and FOXO3a), anti-inflammatory action, and stem cell activity of resident progenitor/notochordal cells (increase in CD24).

CONCLUSIONS

The DWJ-based formulations proposed here provide an adequate stimulus to the resident cells to restart the anabolic machinery.

(YIA) Oxytocin Modulates Osteogenic Commitment in Human Adipose-Derived Stem Cells

Giovannamaria Petrocelli[1], Provvidenza Maria Abruzzo[1], Luca Pampanella[1], Riccardo Tassinari[2], Serena Marini[1], Martina Fazzina[3], Elena Zamagni[1,4], Carlo Ventura[1,5], Federica Facchin[1], and Silvia Canaider[1]

[1] Department of Medical and Surgical Sciences (DIMEC), University of Bologna, Bologna, Italy
[2] Eldor Lab, Bologna, Italy
[3] Department for Life Quality Studies (QUVI), University of Bologna, Rimini, Italy
[4] IRCCS Azienda Ospedaliero-Universitaria di Bologna, Istituto di Ematologia "Seràgnoli", Bologna, Italy
[5] National Laboratory of Molecular Biology and Stem Cell Bioengineering, National Institute of Biostructures and Biosystems (NIBB) c/o Eldor Lab, Bologna, Italy

OBJECTIVE

Human adipose-derived stem cells (hASCs) are harvested in not-invasive contexts and exhibit properties that make them attractive candidates for cell therapy approaches. Identifying natural molecules capable of modulating their biological properties is a challenge for many researchers. Oxytocin (OXT) is a neurohypophyseal hormone involved in health and well-being processes. The aim of this study was to analyze the role of OXT in hASC osteogenesis and adipogenesis, to date poorly investigated, as well as to provide a comprehensive framework of the effects of OXT on hASC proliferation, senescence, and migration.

MATERIALS AND METHODS

hASCs were isolated from adipose tissue of 3 healthy female patients. The effect of 72 hours treatment of OXT on proliferation and cell migration was evaluated by Bromodeoxyuridine (BrdU) and by scratch wound healing assays, respectively. Cell senescence was assessed by Senescence-Associated-β-Galactosidase (SA-β-Gal) activity staining. Gene expression of proliferation, cell cycle, senescence and autophagy marker genes was analyzed by real-time PCR (qPCR). The adipogenic and osteogenic differentiation ability was assessed by Oil Red O and Alizarin Red S staining respectively, and expression analysis of the respective gene markers were performed. The osteogenic commitment was also evaluated by immunofluorescence analyses. The autophagic process was investigated by qPCR also during the osteogenic differentiation protocol.

RESULTS

OXT did not affect hASC proliferation and migratory ability, but increased the SA-β-Gal activity, probably related to the promotion of the autophagy. OXT promoted osteogenic differentiation in a dose-dependent manner, while it reduces adipogenic differentiation at highest dosage. We also observed an increase in autophagy gene markers expression at the beginning of the osteogenic process in OXT-treated hASCs, leading us to hypothesize that OXT could promote osteogenesis by modulating the autophagic process.

CONCLUSIONS

Based on our findings, OXT promotes hASCs osteogenic commitment, acting as a driving force in the early days of the differentiation pathway, and is also able to activate autophagy marker genes in the same period of time. Further in vitro and in vivo studies will be needed to address this wide area of inquiry. OXT could be a promising molecule to use in the field of bone and fracture regenerative medicine.

(YIA) Structural characterization and immunomodulatory properties of spheroids consisting of Urine Derived Renal Epithelial Cells and Wharton's Jelly Mesenchymal Stem Cells

Valeria Pizzuti[1], Francesca Paris[1], Emma Balducelli[1], Pasquale Marrazzo[1], Jacopo Peca[1], Lucio Publio Gallo[1], Francesco Alviano[3], Laura Bonsi[1], Giorgia Comai [2], Gaetano La Manna[1,2]

[1] Department of Medical and Surgical Sciences (DIMEC), University of Bologna, 40126 Bologna, Italy

[2] Nephrology, Dialysis and Renal Transplant Unit, IRCCS-Azienda Ospedaliero-Universitaria di Bologna, 40139 Bologna, Italy

[3] Department of Biomedical and Neuromotor Sciences (DIBINEM), University of Bologna, Bologna, Italy

OBJECTIVE

Kidney transplant is a lifesaving procedure for patients with end stage kidney diseases. Organs derived from cardiac, or brain death donors are increasing, due to the high demand of kidney replacement. These organs are highly exposed to the ischaemia-reperfusion and Acute Kidney Injury events, leading to an increase in kidney cell turnover for replacing damaged cells with functional ones. The damaged or senescent cells lining kidney tubules which are exfoliated and voided in urine are known as Urine-derived Renal Epithelial Cells (URECs), rarely present in the urine of healthy subjects. Our recent results have indicated that URECs from kidney transplanted patients suppressed the proliferation of CD4 and CD8 T Lymphocytes and increased the T regulatory cell subset, during coculture with Peripheral Blood Mononuclear Cells (PBMCs). However, these experiments were carried out in traditional tissue culture plates, which have several limitations compared with more relevant three-dimensional (3D) systems. The aim of this study is to combine URECs with Wharton's Jelly Mesenchymal Stromal Cells (WJ-MSCs) to generate spheroids and to evaluate the resulting formed 3D structure. The immunomodulatory potential of URECs+WJ-MSCs spheroids was also analysed.

MATERIALS AND METHODS

URECs and WJ-MSCs were combined in different ratios, using ultra-low attachment plates to obtain spheroids of 5000 cells each. The spheroids were co-cultured with PBMCs for 72 hours, while PBMCs alone were used as control. The apoptosis of PBMCs, the proliferation and activation of CD4 and CD8 T cells and the regulatory T subset were evaluated by flow cytometry.

RESULTS

URECs and WJ-MSCs successfully aggregate within 24 hours in both 1:1 and 2:1 ratio (URECs: WJ-MSCs), while spheroids consisting of URECs without the mesenchymal cell type did not completely form a defined 3D structure. URECs+WJ-MSCs spheroids did not affect the apoptotic rate of PBMCs, but significantly reduced the activation of CD4 and CD8 T cells. Also, the percentage of regulatory T cells was increased in the presence of the spheroid with a 1:1 ratio.

CONCLUSIONS

The combination of URECs and WJ-MSCs supports the formation of functional 3D cell aggregates, probably due to the higher condensing capacity of WJ-MSCs. Our 3D model shows interesting immunomodulatory properties, which need to be further elucidated by extending the analysis panel for the immune cell population involved, including B lymphocytes and monocytes.

(YIA) Comparative Analysis of Mesenchymal Stromal Cells Isolated from Adult and Paediatric Bone Marrow Donors and expanded in Human Platelet Lysate

Camilla F. Proto[1], Alessia G.S. Banche Niclot[1], Elena Marini[1], Federico Divincenzo[1], Ivana Ferrero [2], Luciana Labanca[3], Franca Fagioli [1,2], Katia Mareschi [1, 2]
[1] Department of Public Health and Paediatrics, The University of Turin, Piazza Polonia 94, 10126 Torino, Italy;
[2] Stem Cell Transplantation and Cellular Therapy Laboratory, Paediatric Onco-Haematology Division, Regina Margherita Children's Hospital, City of Health and Science of Turin, Torino, Italy
[3] Blood Component Production and Validation Centre, City of Health and Science of Turin, S. Anna Hospital, Turin, Italy

OBJECTIVE

The high heterogeneity of Mesenchymal Stromal Cells (MSCs) suggests an improvement of standard protocols of isolation and expansion for their clinical use. We have set a method to isolate MSCs from Bone Marrow (BM) using inactivated Human Platelet Lysate (HPL) in Good Manufacturing Practice (GMP) conditions. In this study we compared the MSCs obtained from adult and paediatric donors and analysed the characteristics of MSCs during the expansion until the 3rd passage.

MATERIALS AND METHODS

After informed consent, BM samples were collected by washing out transplant-dedicated bags from BM donors. Cells were counted and plated in six-multiwell plates to evaluate colony-forming unit fibroblasts (CFU-F) and seeded at a density of 10.000 cells/cm^2 in alpha-MEM containing 10% of inactivated HPL produced from Blood Component Centre and validated under GMP conditions. After detachment, the cells were plated at a density of 1000 cells/cm^2 and evaluated, during the expansion, for CD90, CD105, CD73 and CD45/34/14 immunophenotype expression. Furthermore, the capacity of MSCs to mature into osteoblasts, adipocytes and chondroblasts was analysed with differentiative medium and appropriate staining. Statistical analyses were performed using GraphPad software 9.0.

RESULTS

We isolated MSCs from 43 healthy donors: 20 adults with a median age of 28.5 (range 19-49, 6 females and 14 males) and 23 paediatrics with a median age of 7 (range 1-18, 7 females and 16 males). The CFU-F number was significantly higher in the paediatric group than in the adult one ($p < 0.0084$) while no gender-related differences were observed.

A multivariable analysis showed a correlation between CFU-Fs and the donor age ($p < 0.0001$) and the growth rate at the first passage ($p < 0.0004$), but not in following passages. No significant differences were observed in the immunophenotype and in the differentiative capacity between the two groups.

CONCLUSIONS

In this study we demonstrated that paediatric donors showed a higher number of CFU-Fs than adult BM donors as reported in a previous work in which we isolated MSCs from BM in alpha-MEM containing 10% of foetal bovine serum. However, the use of HPL increases the performance of MSC growth in both groups allowing a good expansion until the 3rd passage without showing significant differences. These data demonstrate that HPL guarantees a standard method for MSC isolation and expansion under GMP conditions.

Mesenchymal stromal cell secretome for regenerative medicine: modulation of soluble factors and miRNAs in extracellular vesicles under different culture conditions for joint pathologies

Enrico Ragni[1], Alessandra Colombini[1], Michela Maria Taiana[1], Paola De Luca[1], Simona Piccolo[1], Giulio Grieco[1], Vincenzo Raffo[1], Laura de Girolamo[1]
[1]IRCCS Ospedale Galeazzi - Sant'Ambrogio, Laboratorio di Biotecnologie Applicate all'Ortopedia, Via Cristina Belgioioso 173, 20157 Milano, Italy

OBJECTIVE
In regenerative medicine approaches related to orthopaedic conditions, mesenchymal stromal cells (MSCs) have shown positive outcomes due to the secretion of therapeutic factors, both free and conveyed within extracellular vesicles (EVs). MSC-derived factors can be modulated by both culture and environmental conditions but a complete fingerprint is still missing in the context of orthopaedic applications. Thus, the aim of this work is to characterize adipose-derived MSC (ASCs)-secreted factors and EV-miRNAs and their modulation after high levels of IFNγ preconditioning or low levels of inflammation, mimicking the synovial fluid of patients with osteoarthritis (OA). Furthermore, the ability of ASC-EVs to penetrate cartilage explants is evaluated.

MATERIALS AND METHODS
ASCs were isolated from four donors and cultured with and without IFNγ (1 ng/ml) or TNFα (5 pg/ml) + IL1β (10 pg/ml) + IFNγ (40 pg/ml) mimicking OA synovial fluid concentration. First, 200 secreted factors were analysed by ELISA. Second, 754 miRNAs were studied by high-throughput qRT-PCR screening in ultracentrifuge-purified EVs. Bioinformatics tools were used to predict the modulatory effect of the identified molecules on pathological cartilage and synovial macrophages. Immunofluorescence and time-lapse coherent anti-Stokes Raman scattering, second harmonic generation and two-photon excited fluorescence were used to follow and quantify the incorporation of fluorescent EVs into cartilage explants.

RESULTS
More than 50 cytokines/chemokines and more than 200 EV-miRNAs could be identified. The majority of molecules are involved in the remodelling of the extracellular matrix and in the homeostasis of inflammatory cells including macrophages, promoting their shift towards an M2 phenotype. Inflammatory priming and synovial fluid-like conditions were able to modulate the secretome ability to stimulate healing and reduce inflammation. Finally, the penetration of EVs in cartilage explants was monitored as a rapid process, starting within minutes of administration and reaching a depth of 30-40 μm after 5 hours and the plateau at 16 hours.

CONCLUSIONS
The secretome obtained from ASCs can be considered as the main vehicle through which the mesenchymal stromal cells transfer their regenerative and anti-inflammatory potential and the preconditioning in an inflammatory environment can further modulate their therapeutic capacities for future applications in the field of joint pathologies such as OA.

Human amniotic mesenchymal stromal cell conditioned medium counteracts cancer associated fibroblasts-related marker expression induced through transforming growth factor beta administration

Jacopo Romoli[1*], Paola Chiodelli[1*], Andrea Papait[1,2], Patrizia Bonassi[3], Silvia De Munari[3], Elsa Vertua[3], Serafina Farigu[3], Antonietta Silini[3], Ornella Parolini [1,2]
1 Department of Life Science and Public Health, Università Cattolica del Sacro Cuore, Rome, Italy
2 Fondazione Policlinico Universitario "Agostino Gemelli" IRCCS, Rome, Italy
3 Centro di Ricerca E. Menni, Fondazione Poliambulanza Istituto Ospedaliero, Brescia, Italy.
*The authors equally contributed to this study

OBJECTIVE
The study investigated whether the secretome of hAMSC (hereafter referred as CM-hAMSC) has the potential to contrast the differentiation of fibroblasts to cancer associated fibroblasts (CAF), thus representing a potential new adjuvant for current therapies such as immunotherapies or chemotherapies.

MATERIALS AND METHODS
In vitro differentiation of normal adult fibroblasts towards CAF was performed by adding exogenous transforming growth factor beta (TGFβ). The acquisition of CAF features was assessed by immunofluorescence for alpha smooth muscle actin (αSMA) expression and for cytoskeleton organization. In addition, we used Real Time PCR and flow cytometry to assess the expression of the most relevant CAF markers. Finally, we evaluated the ability of CM-hAMSC to counteract the acquisition of CAF-like gene expression and functionality.

RESULTS
Exogenous TGFβ up-regulated the expression of αSMA and microfibril associated protein 5 (MFAP5), two gold standard markers of differentiated CAF-like cells. This increase was also accompanied by polymerisation and organisation of αSMA and the deposition of MFAP5, as assessed by immunofluorescence analysis. On the other hand, the same markers were transcriptionally downregulated by the action of CM-hAMSC, and immunofluorescence also showed the lack of protein organization. CM-hAMSC treatment also led to downregulation of CAF-related genes as TGFβ, matrix metalloproteinase-2 (MMP-2) and fibroblast activation protein (FAP). Our study also reported that CM-hAMSC increases the expression of other proteins typically expressed by CAF such as podoplanin (PDPN), tenascin, and interleukin 6 (IL-6).

CONCLUSIONS
Our preliminary results suggest that factors present in CM-hAMSC are able to counteract the effect of TGFβ on fibroblasts, limiting the acquisition of CAF-related markers at different levels. Future experiments will assess the ability of the hAMSC secretome to interfere with functional aspects of CAF behavior such as migration and invasiveness in order to further explore its potential use in the field of oncology.

(YIA) Hybrid spheroids as a model of osteosarcoma

Martyna Malgorzata Rydzyk[1], Micaela Pannella[2], Concettina Cappadone[1], Giovanna Farruggia[1], Emil Malucelli[1], Cristina Pellegrino[1], Francesca Rossi[1], Stefano Iotti[1], Toni Ibrahim[2] and Enrico Lucarelli[2]

[1] Pharmaceutical Biochemistry Lab, Department of Pharmacy and Biotechnology, Alma Mater Studiorum University of Bologna, Via San Donato 19/2 40127 Bologna, Italy.

[2] Osteoncology, Bone and Soft Tissue Sarcomas and Innovative Therapies Unit, IRCCS Istituto Ortopedico Rizzoli, Bologna, Italy

OBJECTIVE

Osteosarcoma (OS) is the most common primary malignant bone tumour. The 5-year survival rate of patients treated with current therapy is 70% when the disease is localized and less than 20% when metastatic. Therefore, new drugs, such as tyrosine inhibitors, must be tested to improve patient's outcome. So far, most of the in-vitro pre-clinical studies have been conducted on a monolayer of OS cells. These models are limited because do not consider the tumour microenvironment (TME) in terms of cell composition and 3D structure. The aim of this work is to develop 3D OS models including different tumor-associated cells (TAC) such as mesenchymal stromal cells, fibroblasts and endothelial cells co-cultured with cancer cells. Mimicking OS TME, we will obtain robust results representing the in vivo tumor behaviour.

MATERIALS AND METHODS

We built spheroids containing OS cells (MG63 and Saos-2) alone or co-cultured with TAC. Spheroids were made by self-assembly in 96-well ultra-low attachment. Spheroids growth was monitored for 7 days by PrestoBlue assay. Spheroids were fixed and embedded in OCT for further histological characterisation.

RESULTS

Results showed that MG63 monocultures developed a compact spheroid with a regular shape, while Saos-2 cells were unable to form a compact spheroid. When mesenchymal stromal cells, fibroblast and endothelial cells were added to cancer cells, the spheroids became more compact and viable, as stromal cells can furnish a physiological scaffold to tumor tissue. Confirming the validity of the model, the structure of the spheroids remains compact and viable over time.

CONCLUSIONS

TAC contributes to OS expansion and exert an impact on invasiveness, thus supporting the idea that these cells promote a tumorigenic effect. The developed spheroids reproduce better the complexity of TME in OS tumors, can enable a more accurate model for high-throughput screening anticancer drugs. Further studies will be conducted using patient's derived primary cells.

(YIA) THE LIFESAVER PROJECT: A BIODIGITAL PLACENTA

Alice Masserdotti[1], Michael Gasik[2], Elsa Vertua[3], Pietro Romele[3], Serafina Farigu[3], Paola Chiodelli[1], Lorena Di Pietro[1], Wanda Lattanzi[1], Antonietta Rosa Silini[3], Ornella Parolini[1,4]

[1]Department of Life Science and Public Health, Università Cattolica del Sacro Cuore, Rome, Italy

[2]Seqvera Ltd., Helsinki, Finland

[3]Centro di Ricerca E. Menni, Fondazione Poliambulanza Istituto Ospedaliero, Brescia, Italy

[4]Fondazione Policlinico Universitario "Agostino Gemelli" IRCCS, Roma, Italy

OBJECTIVE

The overall objective of LIFESAVER is to create a new, digitally cloned in vitro system ("biodigital twin") for emulation of the prenatal conditions in the vicinity of the uterine/placental interface, capable of high biofidelity prediction of safety and risk of substances towards unborn babies. This model mimics the first trimester placenta and will combine physical -in vitro- and digital –in silico– parts.

MATERIALS AND METHODS

Taking into account latest developments in 3D biomanufacturing and organs-on-chip, a new placenta model is designed, combined a physical -in vitro - and a digital – in silico – part. The physical part comprises a biomechanical system, focused on two major transport way REPE (representative placental element) or RACE (representative amnion-chorion membrane element), with microfluidic circuits representing maternal and fetal blood flows where different substances can be injected and their distribution from mother to fetus analyzed. The software part comprises several computer models which communicate with the in vitro part, analyzes the data and can be used for interpolation and prediction.

RESULTS

The starting point for the construction of the model was to identify the characteristics, structure and function of first trimester placenta. Thus, by extensive literature searches and computational simulations several parameters, comprising fetal membranes layers thickness, placenta cell types, placenta tissues biomechanical and physical properties, blood streams parameters, exchange transport surface, and composition of the amniotic fluid, were collected. The ongoing attempt is now to adapt the defined conditions to the in vitro model (e.g. compatibility of bioinks with cells from amniotic membrane to identify optimal bioink for model; microfluidics downscaling of human placenta to fit model-size).

CONCLUSIONS

LIFESAVER 3D manufactured "placental barrier" will generate reproducible in vitro and in silico data, enabling effective screening of chemicals and pharmaceuticals which might affect pregnant women health, reducing animal, preclinical and clinical testing. These data will allow the creation of the scientific knowledge base needed for fostering regulations and the development of a controllable platform for predicting exposure of pregnant women to different chemicals and drugs, capable of the validation at premarketing, risk assessment, use, and revision of the status of existing compounds.

(YIA) Differentiation of human induced pluripotent stem cells into hyalocytes through ascorbic acid treatment

Elena Laura Mazzoldi[1], Rosalba Monica Ferraro[1], Gabriele Benini[1], Piergiuseppe Sacristani[2], Daniele Lussignoli[2], Francesco Semeraro[2], Silvia Clara Giliani[1]

[1] Angelo Nocivelli Institute for Molecular Medicine, Department of Molecular and Translational Medicine, University of Brescia, Viale Europa 11, 25123 Brescia, Italy
[2] Department of Ophthalmology, University of Brescia, Spedali Civili, Piazzale Spedali Civili 1, 25123 Brescia, Italy

OBJECTIVE
Hyalocytes are a tiny population of macrophage-like cells which reside in the vitreous cortex of the eye. Even though hyalocytes have been known for 150 years, they have been poorly investigated. Recently, their involvement in both physiological processes and pathological conditions of the vitreoretinal interface has been suggested. However, essentially hyalocytes from slaughtered animals have been cultured, while studies on cultured human hyalocytes are few, since their isolation requires an invasive surgery. To this reason, the aim of this study is to differentiate hyalocytes from human induced pluripotent stem cells (iPSCs) to have an unlimited source of cells in a non-invasive way.

MATERIALS AND METHODS
iPSCs, routinely cultured on Matrigel-coated multiwell plates, were firstly differentiated into hematopoietic stem cells (HSCs) through a commercial kit, and then into macrophages by macrophage colony-stimulating factor (M-CSF) treatment. Macrophages were then left untreated (NT) or treated with ascorbic acid alone or in combination with basic fibroblast growth factor (bFGF) and/or transforming growth factor beta 1 (TGFβ1). As positive control, macrophages have also been cultured in the presence of a pool of vitreous bodies from vitrectomies. Cells have been analyzed from a morphological point of view, and for gene and protein expression by qRT-PCR, western blot, immunofluorescence, and flow cytometry at day 7, 14, and 21 of culture.

RESULTS
Similar to vitreous-treated cells, macrophages treated with ascorbic acid alone or in combination with bFGF presented a more elongated shape as compared to NT or cells treated with TGFβ1. Moreover, the same treatments induced *S100A4*, *S100A10*, *S100B*, and *CX3CR1* gene expression downregulation, while upregulated *COL6A1* and *HLA-DRA*. At protein level, S100B, CD14, and CD49d resulted downregulated with all the treatments, especially at longer timepoints.

CONCLUSIONS
Hyalocytes can be differentiated by treating iPSC-derived macrophages with ascorbic acid alone for 21 days.

IGFBP5 is released by senescent mesenchymal stromal cells and is internalized by healthy cells promoting their senescence

Valeria Mazzone[1] , Yesuf Adem Siraj[1], Nicola Alessio[1], Umberto Galderisi[1]
[1] Department of Experimental Medicine, Luigi Vanvitelli Campania University, Naples, Italy

OBJECTIVE
Stem cell senescence refers to the aging or deterioration of stem cells, particularly adult stem cells, which are critical for tissue maintenance and repair throughout an individual's life. As stem cells age, their ability to function properly and regenerate damaged tissues decreases. This senescence of stem cells can have significant implications for overall health and is closely linked to the aging process and age-related diseases. The senescence-associated secretory phenotype (SASP) comprises a variety of factors that regulate numerous functions, including the induction of secondary senescence, modulation of immune system activity, remodeling of the extracellular matrix, alteration of tissue structure, and promotion of cancer progression. Identifying key factors within the SASP is crucial for developing effective strategies to counteract cellular senescence which impairs stem cells properties. We analyzed the senescence of mesenchymal stromal cells (MSCs), which is an heterogenous population, comprising stem cells, progenitors and differentiated cells. The aging of MSCs has profound consequences on human health given their role in maintaining tissue homeostasis and repair. Our research has specifically focused on investigating the role of IGFBP5, a component of the SASP observed in various experimental models and conditions.

MATERIALS AND METHODS
Firstly, we confirmed the role of recombinant IGFBP5 (rIGFBP5) protein to onset senescence in MSCs. After, cells were irradiated with 2000 mGy X-rays at room temperature. Following irradiation, we collected Conditioned Medium to evaluate the release of IGFBP5 in the CM and its correlation to an increase in oxidative stress. To assess the pathways involved in IGFBP5 intake, we treated MSCs with rIGFBP5-His Tag at different time points while inhibiting the principal receptors. To investigate the effects of IGFBP5 and RARs on senescence, we treated the cells with r-IGFBP5 in the presence or absence of RARs and performed western blot analysis and immunoprecipitation.

RESULTS
We have demonstrated that IGFBP5 actively contributes to promoting senescence in MSCs. We have gained valuable insights into the mechanisms through which IGFBP5 exerts its pro-senescence effects, which include its release following genotoxic stress, involvement in signaling pathways mediated by reactive oxygen species and prostaglandins, internalization via specialized structures called caveolae, and interaction with a specific protein known as RARα.

CONCLUSIONS
By uncovering these mechanisms, we have advanced our understanding of the intricate role of IGFBP5 in inducing senescence in MSCs. Furthermore, our findings highlight the potential of IGFBP5 as a promising therapeutic target for age-related diseases and cancer.

(YIA) 3D printed scaffold functionalized with bioactive glasses for *in vitro* bone models

Alessandro Mosca Balma[1] , Ilaria Roato[1] , Riccardo Pedraza[1] , Clarissa Orrico[1] , Marta Miola[2], Enrica Vernè[2], Federico Mussano[1]

[1] Department of Surgical Science, C.I.R. Dental School, Università di Torino, Italy.

[2] PolitoBIOMed Lab, Department of Mechanical and Aerospace Engineering, Politecnico di Torino, Italy.

OBJECTIVE

Alterations affecting the bone tissue are responsible for a wide range of pathologies with a high social impact, such as osteoporosis, osteoarthritis, bone metastases. To overcome the actual ethical problem related to the use of animal model for pathological simulation, the work aim to develop a new *in vitro* 3D bone niche model by combining the printability of poly(ε-caprolactone) (PCL) and the bioactivity of bioactive glasses (BGs).

MATERIALS AND METHODS

Two different functionalized borosilicate BGs, one doped w/ copper ions (BG_Cu) and the other w/o, were blended with PCL respectively at 90/10 w/w PCL/BG and 90/10 w/w PCL/BG_Cu and compared each other with pure PCL. The BG were incorporated in PCL matrix through dissolution in chloroform, by solvent casting method, then the blends were 3D printed to generate scaffolds with defined circular geometry and two different infill structures a porous one and a bulk one. ASC52 hTert cells (ASCs) and HMEC 1 cells (HMECs) were seeded on these scaffolds to test the early adhesion and spreading morphology within 24h on material surface, through immunofluorescence microscopy. To evaluate the biocompatibility of the scaffolds, both ASCs and HMECs were cultured for 1, 3 and 7 days to allow scaffold colonization, and registered their growth through the quantitation of the ATP release in culture by viable cells (CellTiter-Glo kit).

RESULTS

All the scaffolds allowed the adhesion of ASCs and HMECs, with a better affinity to the materials filled with BGs. The same behavior was confirmed by the spreading morphology evaluation on immunofluorescence images. The addition of BG/BG_Cu to the PCL matrix promotes the ASCs proliferation, particularly for the scaffolds containing BG w/o copper. Similar behavior was also observed for viability assays with HMECs where the presence of BG/BG_Cu significantly improve the PCL biological properties.

CONCLUSIONS

Filling a printable matrix as the PCL with a non printable bioactive filler as the BG/BG_Cu seems to be better on all fronts compare to the pure materials. Also the different behavior of the selected type of cells in presence or not of Cu ions inside BGs, could be a useful information to take into account for the future implementation of the bone model, where both ASCs and HMECs need to grow together to create a bone niche. Future tests will be focused on the quantification of osteogenic and angiogenic potential of the proposed scaffolds and on the development of co-cultures inside bioreactors.

Intervertebral disc degeneration: an opportunity of cure from new molecular circuits.

Maria Pina Notarangelo[1], Elisabetta Lambertini[1], Letizia Penolazzi[1], Federica Fortuna[1], Maria Giulia Dell'Aquila[1], Anna Chierici[1], Francesco Di Virgilio[2], Roberta Piva[1]
[1] Department of Neuroscience and Rehabilitation, University of Ferrara, 44121 Ferrara, Italy
[2] Department of Medical Sciences, University of Ferrara, 44121 Ferrara, Italy

The mobility of the intervertebral joints is ensured by complex fibrocartilaginous structures, the intervertebral discs (IVDs). The degeneration of the IVD due to aging, injury or trauma is without cure since all approaches are unable to reverse the pathological phenotype of the degenerated IVD cells. In recent years therapeutic options that meet the principles of regenerative medicine are considering strategies aimed at awakening stem/progenitor cells that reside in the IVD microenvironment. We found that exposure to adequate stimuli (hypoxic priming, growth factors from ECM-based scaffold, or molecules from plant extracts) induce cellular responses attributable to stem cell activity. This suggests that the adequacy of a targeted therapy relies on a thorough understanding of key molecules involved in maintaining both the stem cell niche and mature cell function.

OBJECTIVE

We aimed to investigate a potential relationship between: 1. the P2X7 purinergic receptor (P2X7R) as a molecule capable of mediating the effect of extracellular ATP that the IVD cells constantly experience, both to meet physiological needs and as a consequence of degeneration, and 2. NFATc1 and HIF1a transcription factors as mediators of Ca^{++} signaling and hypoxic adaption of IVD.

MATERIALS AND METHODS

IVD cells from biopsies with different level of degeneration were subjected to: gene expression analysis by immunocytochemistry and Western blot; evaluation of P2X7R expression and subcellular localization after transfection with NFATc1/A expression vector to overexpress NFATc1, or $CoCl_2$ exposure to induce hypoxia; chromatin immunoprecipitation assay to investigate the P2X7R gene promoter.

RESULTS

After expansion, IVD cells become de-differentiated resembling the degeneration process and losing their chondrogenic-like phenotype; on the contrary, NFATc1 and P2X7R expression increased. NFATc1 overexpressing cells showed a significant increase of P2X7R. NFATc1 is recruited to the *P2RX7* gene promoter. Notably, after $CoCl_2$ treatment to induce hypoxia, HIF1α occupancy significantly increased causing a decrease in NFATc1 recruitment and P2X7R expression.

CONCLUSIONS

The involvement of NFATc1 and HIF1a in the opposite modulation of P2X7R expression opens the way to consider these pathways as regulators of crucial biological activities in the IVD cell population and, consequently, potential targets for therapies aimed at support repopulation by resident cells and restart the anabolic mechanism.

(YIA) Proinflammatory microenvironment affects biological behavior of cholangiocytes: role of PD-L1 and IL-6

Giulia Orlandi[1], Giulia Bertani[1], Guido Carpino[2], Teresa De Luca[3], Matteo Franchitto[3], Savino Paradiso[2], Rosanna Di Tinco[1], Alessandra Pisciotta[1], Selenia Miglietta[2], Diletta Overi[2], Paolo Onori[2], Vincenzo Cardinale[4], Domenico Alvaro[3], Eugenio Gaudio[2], Gianluca Carnevale[1]

[1]Department of Surgery, Medicine, Dentistry and Morphological Sciences with Interest in Transplant, Oncology and Regenerative Medicine, University of Modena and Reggio Emilia, Modena, Italy;
[2]Department of Anatomical, Histological, Forensic Medicine and Orthopedics Sciences, Sapienza University of Rome, Rome, Italy;
[3]Department of Translational and Precision Medicine, Sapienza University of Rome, Rome, Italy;
[4]Department of Medico-Surgical Sciences and Biotechnologies, Sapienza University of Rome, Rome, Italy

OBJECTIVE

Cholangiocytes (CC), epithelial cells lining the bile ducts, are responsible for bile production and homeostasis. Primary damage on CC and/or microenvironment alterations of the biliary epithelial tissue contribute to the development of cholangiopathies (CPs). Particularly, proinflammatory signals play a central role in the establishment of pathophysiology conditions. To this regard, we aimed to study the immunomodulatory properties of human CC (H69 cell line) in response to an inflammatory microenvironment, mimicked *in vitro* by an indirect co-culture system with activated Peripheral Blood Mononuclear Cells (aPBMCs), to elucidate their involvement in the CPs.

MATERIALS AND METHODS

aPBMCs, isolated from healthy donors, were activated with anti-CD-3/CD-28 antibodies and cultured with H69 cells (10:1 seeding ratio) at different time points through an indirect transwell co-culture system. The activation of immunomodulatory pathways (PD-1/PD-L1), cell cycle, senescence, and epithelial-mesenchymal transition (EMT) markers were investigated in H69 by Real-Time PCR (qPCR), Western Blot (WB) and Immunofluorescence analyses. Besides, the inflammatory pattern was assayed in aPBMCs.

RESULTS

IL-6/JAK/STAT3, NF-kB, and PD-L1 expression was significantly up-regulated in H69 after co-culture with aPBMCs. qPCR data showed a decrease of cyclin B and an increase of cyclin D and up-regulation of p21. In parallel, WB analyses highlighted a reduced PCNA expression, suggesting a cell cycle arrest in H69 exposed to an inflammatory microenvironment. Moreover, H69 underwent EMT losing the epithelial markers (EpCAM, β-catenin), and gaining a mesenchymal phenotype (vimentin, α-SMA). Finally, a decreased expression of IL-2 and proinflammatory cytokines, IL-6, IL-10, IFNγ, TNFα, was detected in aPBMCs after H69 co-culture. These events might be due to the activation of compensatory pathways that synergistically allow H69 to modulate the inflammatory microenvironment and support their EMT upon fibrosis establishment and progression to CPs.

CONCLUSIONS

CC can modulate inflammation by activating IL-6 and PD-1/PD-L1 pathways. Our results pave the way for future insights on cross-talk of these pathways, aiming to investigate the pathophysiological and molecular mechanisms involved in the cascade of cholangiopathies. Further evidence will provide the basis to develop new therapeutic tools for the treatment of cholangiopathies.

(YIA) Periodontal ligament-derived stem cells (PDLSCs) isolated from patients with periodontitis show a senescent phenotype that can be rescued by RG108

Clarissa Orrico[1], Ilaria Roato [2], Giacomo Baima [2,3], Alessandro Mosca Balma [2], Daniela Alotto [4], Federica Romano [2], Riccardo Ferracini [5], Mario Aimetti [2], Federico Mussano[2]

[1] Fondazione Ricerca Molinette - Onlus, A.O.U. Città della Salute e della Scienza, Turin, Italy.

[2] Department of Surgical Sciences, University of Turin, Turin, Italy.

[3] Politecnico di Torino, Turin, Italy.

[4] Skin Bank, Department of General and Specialized Surgery, A.O.U. Città della Salute e della Scienza, Turin, Italy.

[5] Department of Surgical Sciences and Integrated Diagnostics, University of Genova, Genova, Italy.

OBJECTIVES

Periodontitis is a common disease, which at last leads to loss of teeth, thus regenerative treatments aim at rescuing the damaged periodontal ligament (PDL) are envisaged. In this work we investigated PDL-derived mesenchymal stem cells (PDLSCs) isolated from both healthy donors (hPDLSCs) and periodontitis patients (pPDLSCs) to ascertain any functional differences owing to their milieux of origin, and to understand their actual employ in treatment of periodontitis.

METHODS

PDLSCs were tested in terms of colony-forming unit efficiency; multidifferentiating capacity. PDLSC immunophenotype was checked at different passages by flow cytometry. Stemness, and senescent state was studied by gene expression, flow cytometry, immunofluorescence, and β-galactosidase staining. PDLSCs were treated or not with RG108, a selective small molecule inhibitor of DNA methyltransferase (DNMT), at 50 and 100uM for 5 days.

RESULTS

The immunophenotype of hPDLSCs and pPDLSCs was comparable as well as their multilineage differentiation capabilities, nonetheless pPDLSCs showed a marked senescent phenotype soon after isolation, expressing p16 and p21 and being positive for β-galactosidase. This subset of senescent cells expressed also stemness genes, such as OCT4, SOX2 and NANOG. We investigated whether RG108 treatment was able to reverse the senescent phenotype. No significant effects were observed on proliferation and apoptosis for both hPDLSCs and pPDLSCs after RG108 treatment. RG108 induced a significant reduction of both p16 and p21 along with an increase of SOX2 and OCT4 in pPDLSCs, resulting in a decrease of the subset of pPDLSCs co-expressing OCT4 and p21. RG108 also increased the adipogenic potential in pPDLSCs, while interferences were not detected on chondrogenic and osteogenic potentials.

CONCLUSIONS

pPDLSCs contained a subset of stem/senescent cells, which can be treated with RG108 to obtain a rejuvenation, since they significantly reduced senescent markers and up-regulated stemness one. These data open new perspectives for the potential treatment of periodontitis in situ, indeed it is conceivable that local injection of RG108 could avoid the loss of PDL.

Evaluation of Mechano-mimetic Acrylamide Polymers Ability to Drive the Fibroblast-to-myofibroblast Transition

Francesco Palmieri [1], Martina Parigi [2], Alice Cappitti [3], Rachele Garella [1], Alessia Tani [2], Flaminia Chellini [2], Martina Salzano De Luna [4], Daniele Martella [5], Chiara Sassoli [2] , Roberta Squecco [1].

[1] Department of Experimental and Clinical Medicine, Section of Physiological Sciences, University of Florence, 50134 Florence, Italy
[2] Department of Experimental and Clinical Medicine, Section of Anatomy and Histology, Imaging Platform, University of Florence, 50134 Florence, Italy
[3] Department of Chemistry "Ugo Schiff" , University of Florence, 50019 Sesto Fiorentino, Italy
[4] Department of Chemical, Materials and Industrial Production Engineering, University of Naples, 80125 Napoli, Italy
[5] European Laboratory for Non-Linear Spectroscopy (LENS), 50019 Sesto Fiorentino, Italy Istituto Nazionale di Ricerca Metrologica (INRiM), 10135 Torino, Italy

Mechano-mimetic materials are particularly attractive for providing in vitro models for fibroblast-myofibroblast (Myof) transition. This represents a key process in the physiological wound healing and is regarded the "core cellular mechanism" of pathological fibrosis in different organs. In vivo mechanical cues, especially those from the extracellular matrix (ECM), are determinant together with the chemical ones, such as the pro-fibrotic transforming growth factor (TGF)-β1, in promoting fibroblast differentiation. The aim of this in vitro study is to replicate such a process, using polymeric hydrogels as mechano-mimethic scaffold in comparison to conventional glass supports for fibroblast cell culture. The latter support in fact, do not account for the tissue specific ECM mechanics. To this aim, different mechano-mimetic substrates of polyacrylamide (PA) gels have been prepared by free radical polymerization, using acrylamide and N,N′-methylenbis(acrylamide) as monomers, with an accurate tailoring of water content and crosslinking degree, in order to modulate the mechanical properties. The resulting stiffness ranged from 29 to 1 kPa. Murine NIH-3T3 fibroblasts were cultured in the absence or presence of TGF-β1 on glass coverslips coated with these PA hydrogels. After 48h of culture, cells were subjected to morphological (cell shape and area, stress fiber assembly, α-smooth muscle actin expression by confocal laser scanning microscopy) and functional/electrophysiological analyses (cell membrane passive properties and ion currents evaluation by whole cell patch-clamp), to estimate the acquisition of a differentiated Myof phenotype.

Our results suggest that materials with Young modulus less than 1 kPa (soft) triggered an efficient mechanical stimulus to induce fibroblast differentiation towards a Myof phenotype, better than stiffer ones, even in the absence of the chemical stimulus TGF-β1, potentially mimicking the mechanical properties of ECM soon after wounding in vivo.

These findings may open the way to high-throughput in vitro Myof models with the great advantage of using biomaterials reproducible on a large scale. In addition, since the acrylamide monomers are commercially available and the technique used to create the scaffolds is easy, these hydrogels could be suitable to a wide range of applications, overcoming the major drawbacks of other mechano-mimetic supports, such as the low material reproducibility and complex synthesis.

(YIA) Perinatal Stem Cell Spheroids in Type 1 Diabetes Therapy: Structural Insights and Immunomodulatory capacity

Francesca Paris[1], Valeria Pizzuti[1], Pasquale Marrazzo[1], Aurora Longhin[1], Laura Bonsi[1] and Francesco Alviano[2]
[1] Department of Medical and Surgical Sciences, University of Bologna, Italy
[2] Department of Biomedical and Neuromotor Science, University of Bologna, Italy

OBJECTIVE

Type 1 diabetes mellitus (T1DM) is a complex metabolic disorder characterized by the immune-mediated depletion of insulin-producing cells. Currently, the only effective treatment involves the daily administration of exogenous insulin. To address the underlying immunological trigger of T1DM remains a challenge, leading to a significant focus on advancing stem cell therapy. The growing interest in employing three-dimensional (3D) cell cultures to more accurately emulate in vivo conditions can be a strategy to enhance the immunomodulation and differentiation potential of cell-based treatments. The aim of this study is to establish a reliable 3D stem cell-based model that can be investigated to evaluate its immunomodulatory capacity, a potential cell therapy application in T1DM.

MATERIALS AND METHODS

To pursue this aim, we created a co-culture spheroid made of amniotic epithelial cells (AECs) and Wharton’s jelly mesenchymal stromal cells (WJ-MSCs), assembling cells in a 1:1 ratio. The resulting spheroid was analyzed for viability, extracellular matrix production, and hypoxic state. Furthermore, we evaluated the immunomodulatory ability of spheroids by co-culturing them with activated PBMCs or T cells. After 72 hours of co-culture PBMCs or T-cells were recovered and analyzed with flow cytometry.

RESULTS

Our findings suggest that co-culture spheroids are stable during long-term culture while maintaining viability, producing a consistent extracellular matrix, as observed by immunofluorescence staining. The cellular model modulated activated PBMCs and T-cells by reducing their proliferation and activation, without affecting the viability of immune cells. Additionally, co-culture spheroids promote a tolerogenic response by enhancing regulatory T-cell proliferation compared to control PBMCs.

CONCLUSIONS

The obtained co-culture model holds the potential as an encouraging cellular therapy solution for T1DM and regenerative medicine. The assessment of the whole immunomodulatory and differentiative capacities of such a model requires further elucidation, necessitating an increase in the evaluation of the immune cell population involved in T1DM research.

(YIA) Morphological and intensity-based features for radiomics analysis of 3D multicellular spheroids used in high-content screening experiments.

Mariachiara Stellato [1], Filippo Piccinini [2,3,*], Jae-Chul Pyun [4], Misu Lee [5,6], Bongseop Kwak [7], Daniel Remondini [1], Giovanni Martinelli [3], Gastone Castellani [2].
[1] Department of Physics and Astronomy "Augusto Righi" (DIFA), University of Bologna, Bologna, Italy.
[2] Department of Medical and Surgical Sciences (DIMEC), University of Bologna, Bologna, Italy.
[3] IRCCS Istituto Romagnolo per lo Studio dei Tumori (IRST) "Dino Amadori", Meldola, Italy.
[4] Department of Materials Science and Engineering, Yonsei University, Seoul, South Korea.
[5] Division of Life Sciences, College of Life Science and Bioengineering, Incheon National University, Incheon, South Korea.
[6] Institute for New Drug Development, College of Life Science and Bioengineering, Incheon National University, South Korea.
[7] College of Medicine, Dongguk University, Seoul, South Korea.
[*] Corresponding author: Filippo Piccinini, email: f.piccinini@unibo.it; filippo.piccinini@irst.emr.it

OBJECTIVE
In oncology and regenerative medicine, 3D multicellular spheroids are a fundamental in vitro tool for studying the properties of drugs and treatments and making expectations for the effects in in vivo tissues. In particular, 3D multicellular spheroids are becoming extensively used in high-content screenings (HCS) and several morpho-biological features are typically estimated, even from a single 2D widefield image, for further radiomics analysis.

MATERIALS AND METHODS
Today, several software and tools are available for extracting morphological features from spheroid images, but all of them have pros and cons and there is not a general validated solution. The features extracted are typically diameters, area, volume, circularity, sphericity and other morphology-based ones mainly derived from a binary segmentation of the spheroid's border, and just a few tools also consider intensity-based features that can be interesting also for evaluating the presence and the extension of an eventual necrotic core of the spheroid. Starting from the second version of AnaSP (i.e. AnaSP 2.0), an open-source software designed for estimating several morphological features of 3D spheroids by analyzing single 2D widefield images, we released AnaSP 3.0 that includes a new module for extracting intensity-based features and estimating the number of living cells composing the spheroid.

RESULTS
We performed several experiments to compare with ground truth the prediction on the cell distribution in spheroids created with cancer cells, fibroblasts, stem cells and co-cultures of those. Predictions were obtained by evaluating with AnaSP 3.0 single 2D widefield images acquired with an inverted brightfield microscope. Ground truth was obtained by sectioning the spheroids with a cryostat. The differences between ground truth and predictions obtained with AnaSP 3.0 were in the range of 10%.

CONCLUSIONS
We released AnaSP 3.0, a version of the tool optimized for performing extended HCS radiomics analysis with 3D spheroids. The source code, standalone versions, user manual, sample images, video tutorial, and further documentation are freely available at: https://sourceforge.net/p/anasp.

(YIA) - EQUINE MESENCHYMAL STROMAL CELL-DERIVED EXTRACELLULAR VESICLES: VALIDATION OF A PANEL OF RELIABLE MARKERS

Alessia Sulla[1#], Gabriele Scattini[1#], Gianluca Moretti[2], Luisa Pascucci[1]

[1] Department of Veterinary Medicine – University of Perugia - Italy

[2] Cellavos AB – Sweden

[#] Equal contribution

OBJECTIVE

The use of Extracellar Vescicles (EVs) from Mesenchymal Stromal Cells (MSCs) is gaining interest as a cell-free therapeutic approach in horses. However, research on EVs is hampered by the lack of standardised techniques for isolation and characterisation. In veterinary medicine, this problem is exacerbated by the lack of cross-reacting and specific antibodies, which, even when available, sometimes do not react with EVs from domestic animals as indicated in the data sheet. For this reason, we have characterised EVs from equine MSCs (eqMSCs) to identify a panel of markers that work in this species. In addition, we assessed the quality of the EVs isolation protocol by morphological examination, Nanoparticle Tracking Analysis (NTA) and a pilot study on their functional activity by MTT assay on equine tenocytes.

MATERIALS AND METHODS

EVs were obtained from the conditioned medium of adipose-derived and Wharton's jelly-derived eqMSCs. EVs isolation was performed with two different techniques: by ultracentrifugation at 100.000 g and by ultrafiltration using Merck Amicon™ Ultra Centrifugal Filter 100 kDa. The presence, purity, and morphology of EVs were examined by Transmission Electron Microscopy (TEM). The concentration and size distribution of the isolated EVs were determined using a Malvern Panalytical NanoSight NS300 (NTA). The MTT assay on equine tenocytes was performed to verify the functional activity of different concentration of EVs after isolation. Western blotting analysis was performed for the following EVs markers: CD9, CD63, CD81, TSG101, Syntenin, Alix, Mitofilin, and Calnexin. Cell lysates from human and/or murine MSCs were used as positive controls.

RESULTS

At TEM, EVs were round-shaped and ranged in size from 30 to 300 nm. The MTT assay showed a dose-dependent increase in metabolic activity of equine tenocytes. Western blot showed the expression of specific markers consistent with MISEV guidelines, but not the expression of other markers that were expected to be detected. This result was qualitatively independent by the cell source and purification method.

CONCLUSIONS

We herein provided a panel of antibodies that works with horse species and allows standardisation of EVs formulation and reproducibility of data. We also showed that equine tenocytes increase their metabolic activity when treated with different concentrations of EVs.

TARGETING SENESCENT MESENCHYMAL STROMAL CELLS BY SENOTHERAPY.

Gabriella Teti[1], Valentina Gatta[1], Maria Laura Alfieri[1], Alessandra Ruggeri[1], Mirella Falconi[2]
[1]Department of Biomedical and Neuromotor Sciences, University of Bologna
[2] Department of Medical and Surgical Sciences, University of Bologna

OBJECTIVE

Mesenchymal stromal cells (MSCs) have emerged as promising cell-based therapies in regenerative medicine and in the treatment of degenerative and inflammatory diseases due to their ability to self-renew, modulate immune response and differentiate into multiple cell lineages. However, despite accumulating evidence regarding the wide functional abilities of MSCs, their clinical application is hampered by inconsistencies in therapeutic efficacy. Phenotypic, functional heterogeneity and their natural susceptibility to senescence during aging is thought to limit their success in clinical use.

Cellular senescence is commonly defined as a permanent cell cycle arrest in metabolically active cells. While senescence has protective and reparative roles, it can also contribute to the functional decline of tissues.

Senotherapy is an emerging therapeutic strategy that targets cellular senescence delaying the aging process. Senotherapy includes senolytic agents, which selectively induce senescent cell death, and senomorphic agents, which indirectly suppress senescence by inhibiting senescence-associated secretory phenotypes (SASP) and delay the progression of senescence and tissue dysfunction. A promising senolytic agent is quercetin, a natural flavonoid that inhibits cell proliferation. Being mainly studied for its antioxidant effects in cancer, allergic reactions, inflammation, and cardiovascular disorders its precise anti-MSC senescence mechanism is still not clearly identified. The aim of this study was to evaluate the effect of quercetin as a senolytic molecule in counteracting cellular senescence.

MATERIALS AND METHODS

Human MSCs were induced to senescence by H_2O_2 treatment. Different concentrations of quercetin were tested to evaluate the effects on the expression of senescent cell cycle arrest related markers p53, $p21^{WAF1/CIP1}$. Apoptosis and autophagy markers were also evaluated.

RESULTS

Results showed a strong activation of autophagy and apoptosis in senescent cells compared to control ones.

CONCLUSIONS

The data presented underlined the potential role of quercetin as a senolytic therapeutic agent in selectively targeting senescent cells

MESENCHYMAL STROMAL CELL SECRETOME CAN MITIGATE AGE-RELATED OVARIAN DYSFUNCTIONS.

Maria Assunta Ucci[1], Serena Marcozzi[1], Giulia Salvatore[1], Francesca Gioia Klinger[2], Vincenzo Campanella[3], Antonio Libonati[3], Cosimo Tudisco[2], Massimo De Felici[1], Antonella Camaioni[1]
[1]Department of Biomedicine and Prevention, University of Rome Tor Vergata, Rome, Italy
[2]Saint Camillus International, University of Health Sciences, Rome, Italy
[3]Department of Clinical Sciences and Translational Medicine, University of Rome Tor Vergata, Rome, Italy

OBJECTIVE

The aim of the present study is to determine whether conditioned medium (CM) of human Mesenchymal Stromal Cells (MSCs) derived from Adipose Tissue (ADSCs) and Dental Pulp (DPSCs), generally waste material, is able to mitigate the effects of aging on ovarian dysfunctions in a mouse model and to identify specific components of the secretome to which this effect can be attributed.

MATERIALS AND METHODS

ADSCs and DPSCs were obtained from healthy patients, and CMs were collected after 3 days of MSC culture. The protocol consisted in IV administration of 5 ml/Kg of CMs in aged 129/Sv females (8 months), every other day for three times, with one repetition after four weeks. One month later, some females were sacrificed and ovaries collected to perform follicle count, morphological analysis and gene expression, while other females were mated to follow their reproductive output. The microRNAs present in CM-DP and CM-AD were analyzed and their expression patterns compared to assess the possible role in counteracting age-related ovarian features.

RESULTS

The analysis of histological sections showed an increase in the number of total follicles, mainly primordial and primary follicles, compared to control group. In addition, the treatment increased the pregnancy rate (CTRL=8.33%±5.27; DPSC-CM=25.00%±9.13; ASC-CM=60.67%±10.27), the average number of pups delivered per mating cycle and the duration of the entire reproductive life. We verified the improvement of oestral cyclicity in both treatments, with a significant decrease in the percentage of diestrus (CTRL=82.41%±0.59; DPSC-CM=40.91%±4.54; ASC-CM=31.82%±4.54). Moreover, the transcription levels of the pro-inflammatory genes TNFα, IL6 and IL1β and the number of mast cells in the ovarian stroma were significantly decreased in CM-treated females compared to controls. We then identified possible interactions between murine mRNAs and the miRNAs contained in the CMs, thereby associating the target genes with specific cellular pathways. Interestingly, miRNAs more expressed in both CMs harbored binding sites for genes involved in follicle pool dynamics, apoptosis, follicular atresia and inflammatory processes.

CONCLUSIONS

These data indicate that treatment with MSC-CM may mitigate ovarian aging and improve the functional status of the entire reproductive system, probably acting on the overall inflammatory state of the organism and, locally, on the ovarian reserve and the oocyte development potential.

(YIA) MiRNomes secreted by human Mesenchymal Stromal Cells of different origins: their analysis with particular focus on pathways deregulated during ovarian aging.

Alessia Ventura[1], Maria Assunta Ucci[1], Andrea Galgani[2], Stefano Pirrò[2], Serena Marcozzi[1], Massimo De Felici[1], Vincenzo Campanella[3], Antonio Libonati[3], Giulia Salvatore[1], Mario Picozza[4], Giovanna Borsellino[4], Cosimo Tudisco[5], Francesca Gioia Klinger[5], Antonella Camaioni[1]
[1]Department of Biomedicine and Prevention, University of Rome Tor Vergata, Rome, Italy
[2]CIMETA, University of Rome Tor Vergata, Rome, Italy
[3]Department of Clinical Sciences and Translational Medicine, University of Rome Tor Vergata
[4]Neuroimmunology Unit, Santa Lucia Foundation I.R.C.C.S., Rome, Italy
[5]Saint Camillus International University of Health Sciences, Rome, Italy

OBJECTIVE
The aim of the present study was to identify microRNAs (miRs) present in the conditioned media (CMs) of MSCs isolated from adipose tissue (ADSCs) and dental pulp (DPSCs). We identified those highly (HE) and differentially (DE) expressed among CMs and evaluated their biological effects on pathways deregulated in the aged ovary.

MATERIALS AND METHODS
Following the extraction of ADSCs (n=4) and DPSCs (n=2) from healthy patients with two different methods (enzymatic digestion and mechanical fragmentation), we verified their mesenchymal features by analyzing surface markers by flow cytometry and their ability to undergo trilinear differentiation. To obtain the CMs of interest, MSCs were cultured in FBS-free αMEM medium for 3 and 7 days. We extracted the total RNA from the CMs using a commercial column purification kit and sequenced samples on the Illumina platform. After detecting the HE and DE miRs, the TargetScan web tool identified the binding sites of miRs to the UTR region of gene transcripts, while the Enrichr web tool found the pathways deregulated by them.

RESULTS
Since all cells secrete a multiplicity of miRs, we identified and compared the expression patterns of those present in the CMs of the two cell types under investigation. The PCA analysis for the expression of 1028 identified mature miRs showed that the organ source was the most critical factor responsible for the separation of miRs into two distinct groups, while the extraction method did not substantially affect miRNomes within the tissue type. We then performed a statistical analysis to detect the HE/DE miRNAs among the 3-day CMs that have been used in *in vivo* experiments and have showed the capacity to counteract ovarian aging in 8-10 month-old mice as well as lengthening their reproductive life. Interestingly, miRs with high expression in both CMs, and those most highly expressed in each of them, showed binding sites for genes involved in signaling pathways related to follicle ovarian reserve (FoxO, PI3K-Akt), follicular atresia (apoptosis, P53), and inflammatory processes (IL-2, TNF). Moreover, the study of DE miRs among the CMs of 3 and 7 days revealed miRs enriched or degraded after 7 days of conditioning.

CONCLUSIONS
The analysis of miRs secreted in CMs of MSCs highlighted their fundamental role in counteracting degenerative processes associated with ovarian aging and the consequent possibility of using the as therapeutic candidates for age-associated dysfunction and beyond.

(YIA) A new explorative model to study extracellular vesicles action in the recovery of epithelial barrier integrity in Inflammatory Bowel Diseases

Alice Zaramella[1,2], Agner Henrique Dorigo Hochuli[1,2], Matteo Marin[1,3], Paola Bisaccia[1,2,] Miriam Duci[1,2], Marcin Jurga[4], Michela Pozzobon[1,2]
[1] Stem Cell and Regenerative Medicine Lab, Institute of Paediatric Research Città della Speranza, Padova, Italy
[2] Department of Women and Children Health, University of Padua, Italy.
[3] Department of Biotechnology, University of Verona, Italy.
[4] Exobiologics (NV), Belgium.

OBJECTIVE
Inflammatory bowel disease Inflammatory bowel diseases (IBD) such as ulcerative colitis and Crohn's Disease are chronic inflammatory diseases of unknown etiology. Novel approaches for studying epithelial barrier repair remain essential to investigate IBD and to improve therapeutic strategies. The Mesenchymal Stem cells-derived extracellular Vesicles (MSC-EVs) have shown therapeutic properties, including the potential to repair damaged tissues and modulate inflammation.
We aimed to evaluate the capability of the colorectal cancer cell lines Caco-2 in generating a multicellular layer structure (MSL) as an *in vitro* model that could better mimic IBD in human conditions and to evaluate the protective role of MSC-EVs in epithelial barrier reparation.

MATERIALS AND METHODS
MLS were generated using Caco-2 cells and fibroblasts, seeded in the ultra-low attachment plates in 70:30 proportion. The IBD damage was assessed using 100ng/mL of a pro-inflammatory cytokines cocktail (TNF-alpha, Interleukin-6, and Interleukin-1beta). The $1.0x10^9$ of MSC-EVs was administered. A viability test, immunofluorescence staining for phalloidin, and E-cadherin were performed to characterize the structure. The gene expression for MLS characterization, pro-inflammatory and anti-inflammatory pathways were analyzed by qRT-PCR for *LGR5a+, MUC2, OCCLUDIN, TGF-beta RECEPTOR, TNF-alpha RECEPTOR, and INTERLEUKIN-10* before and after IBD induction and EVs treatment.

RESULTS
The MLS formation started on day 3 and was completed on day 6. The administration of the pro-inflammatory cocktail led to a reduction of *LGR5a+* and *OCCLUDIN* expression and an over-expression of *MUC2* and inflammatory pathways. *OCCLUDIN* and *MUC2* expression was significantly improved after EVs treatment. Moreover, the anti-inflammatory action of EVs was demonstrated by the increase of the *INTERLEUKIN-10* as well as the decrease of *TNF-alpha RECEPTOR* gene expression.

CONCLUSIONS
This is the first study recreating the glandular epithelium-like structure *in vitro* in 6 days. We demonstrated the role of the stromal cells in forming three-dimensional (3D) structures and the role of MSC-EVs in modulating the spheroid environment. These results can open a new window for a new 3D model to study new therapies against IBD.

SELECTED POSTER COMMUNICATIONS
Here we insert the selected poster communications.

POSTER COMMUNICATIONS

Here we insert the poster abstracts
